# Homotropic Cooperativity of Midazolam Metabolism by Cytochrome P450 3A4: Insight from Computational Studies

**DOI:** 10.1021/acs.jcim.1c00266

**Published:** 2021-04-22

**Authors:** Junhao Li, Yue Chen, Yun Tang, Weihua Li, Yaoquan Tu

**Affiliations:** † Department of Theoretical Chemistry and Biology, School of Engineering Sciences in Chemistry, Biotechnology and Health (CBH), KTH Royal Institute of Technology, SE-106 91 Stockholm, Sweden; ‡ Shanghai Key Laboratory of New Drug Design, School of Pharmacy, East China University of Science and Technology, Shanghai 200237, China

## Abstract

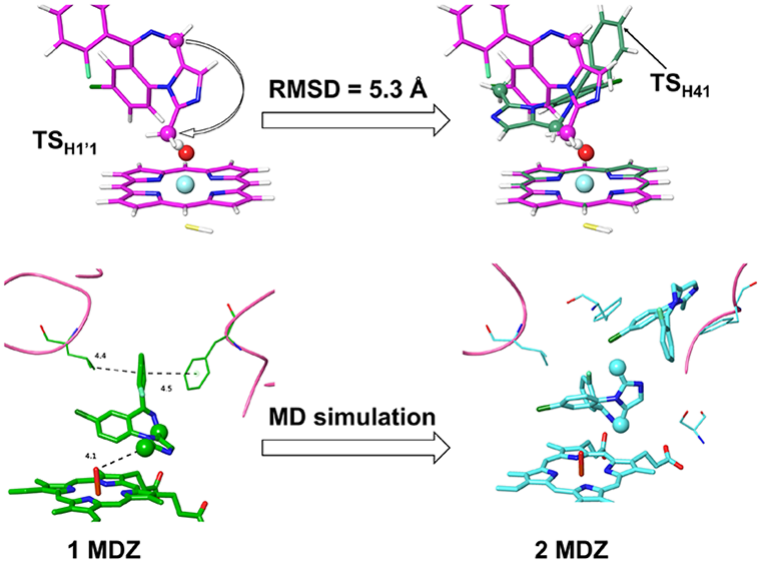

Human cytochrome P450 3A4 (CYP3A4) is responsible for the metabolism of ∼50% clinically used drugs. Midazolam (MDZ) is a commonly used sedative drug and serves as a marker substrate for the CYP3A4 activity assessment. MDZ is metabolized by CYP3A4 to two hydroxylation products, 1′-OH-MDZ and 4-OH-MDZ. It has been reported that the ratio of 1′-OH-MDZ and 4-OH-MDZ is dependent on the MDZ concentration, which reflects the homotropic cooperative behavior in MDZ metabolism by CYP3A4. Here, we used quantum chemistry (QC), molecular docking, conventional molecular dynamics (cMD), and Gaussian accelerated molecular dynamics (GaMD) approaches to investigate the mechanism of the interactions between CYP3A4 and MDZ. QC calculations suggest that C1′ is less reactive for hydroxylation than C4, which is a pro-chirality carbon. However, the 4-OH-MDZ product is likely to be racemic due to the chirality inversion in the rebound step. The MD simulation results indicate that MDZ at the peripheral allosteric site is not stable and the binding modes of the MDZ molecules at the productive site are in line with the experimental observations.

## Introduction

Ligand cooperativity is frequently found in the reactions catalyzed by cytochrome P450 enzymes (P450s).^1−5^ The cooperative binding of ligands can change the kinetics profile of the catalytic reactions, resulting in a hyperbolic or sigmoidal curve on the graph of reaction rate versus substrate concentration.^3^ It has been proposed that the occurrence of P450 cooperativity originates from the simultaneous binding of multiple substrate and/or inhibitor molecules.^6^ In the case that the bound ligands are the same, it is referred to as homotropic cooperativity, and otherwise, it is referred to as heterotropic cooperativity.^3^ In addition to the large and flexible productive site above the heme cofactor, allosteric sites of P450s have also been suggested to be involved in the cooperative ligand binding.^4^ These features make the ligand cooperativity one of the most mysterious phenomena in P450-mediated metabolism.^7−9^

Binding cooperativity has been extensively observed in the reactions catalyzed by CYP3A4, which metabolizes about 50% of the drugs in market.^1,2,10−13^ The cooperative binding behaviors of several canonical CYP3A4 substrates, such as amitriptyline, testosterone, and nifedipine, are known to be sensitive to the experimental conditions and the sources of the enzyme.^14^ Studies have been carried out for unraveling the binding modes of these substrates to CYP3A4.^11,15,16^ The cooperative ligand binding at the productive site of CYP3A4 has also been observed in the available crystal structures (see PDB: 2V0M, 4K9U, and 4D6Z).^17−19^ Some other experimental and computational studies indicate that the cooperative binding may take place at the peripheral allosteric site, which is in the middle of the productive site and membrane interaction area.^20−22^ Also, there are three crystal structures of CYP3A4 with a steroid molecule bound at this site (see PDB: 1W0F, 5A1P, and 5A1R).^23,24^ These efforts provide a vast amount of information for understanding the aberrant catalytic kinetics caused by the cooperative ligand binding.

The cooperative binding of substrates changes not only the kinetics profiles but also the selectivity of the catalytic reactions. One typical example is the metabolism of midazolam (MDZ) by CYP3A4. MDZ is commonly used for sedation in emergency rooms and for treating acute agitation, status epilepticus, and generalized seizure.^25^ MDZ also serves as the *in vitro* marker and clinical index substrate for the activity assessment of CYP3A enzymes. MDZ is metabolized by CYP3A4, which generates two products, 1′-OH-MDZ and 4-OH-MDZ. Under a low concentration of MDZ, the major product is 1′-OH-MDZ, while the proportion of 4-OH-MDZ increases simultaneously with the increase of MDZ concentration.^11^ Some studies have reported that the 1′-OH-MDZ/4-OH-MDZ ratio can also be altered by introducing residue mutations, or adding a heterotropic effector.^20,21,26^ The C4 carbon of MDZ is a prochiral center, but the regioselectivity is still not clear.

Besides the configuration of the hydroxylation products, the mechanism of how MDZ molecules cooperatively bind to CYP3A4 is also elusive. Allosteric modulators for CYP3A4, such as fluconazole, have been identified indirectly in experimental studies,^20^ which suggest that the peripheral allosteric site can be involved in the heterotropic cooperativity. The crystal structure of CYP3A4-MDZ has been solved (PDB: 5TE8) later on,^27^ in which the F′-helix is collapsed and sinks into the productive site, resulting in the binding of additional MDZ molecules and the hydroxylation at C4 being blocked. From this structure, it was deduced that the expansion of the CYP3A4 productive site is necessary for generating the 4-OH-MDZ product.^27^ Computational studies have also been carried out to investigate the dynamics of cooperative binding of MDZ to CYP3A4, but the geometrical results are analyzed based on the biased MD simulations.^28^ It is also not clear if the peripheral allosteric site of CYP3A4 plays a role in the homotropic cooperativity of MDZ metabolism.

In this study, we aim to understand the mechanism of the cooperative dynamics of MDZ hydroxylation by CYP3A4. Quantum chemistry (QC) calculations were first carried out to evaluate the activation barriers for the potential sites of metabolism (SOMs) of MDZ and to derive the corresponding geometries of the intermediates (IMs) and product complexes (PCs). Thereafter, extensive molecular dockings were performed to obtain the initial binding modes of multiple MDZ molecules at the productive and allosteric sites of CYP3A4, followed by long-time molecular dynamics (MD) simulations for the top-1 ranked binding poses. Finally, Gaussian accelerated MD (GaMD) simulations were carried out to further evaluate the energy profiles of MDZ binding to the productive site of CYP3A4.

## Methods

### Quantum Chemistry (QC) Calculations

All the QC calculations were carried out with the Gaussian 09 package (Rev. D.01).^29^ Density functional theory (DFT) with the B3LYP functional^30,31^ was used to calculate the activation barriers for breaking the corresponding C–H bonds of the potential SOMs. In geometry optimization, the 6-31g(d,p) basis set was employed for all the atoms except for the iron, for which the LANL2DZ pseudopotential/basis set^32^ was used. Each initial transition state (TS) structure was located by flexible potential energy surface (PES) scan, followed by the full TS optimization procedure implemented in the Gaussian program, and confirmed by the vibrational frequency calculation. The intrinsic reaction coordinate (IRC) calculation was carried out for the TS structure,^33^ after which the geometries of the two end points were subject to the full optimization for locating the reactant complex (RC) and intermediate (IM) species. The final energy for each species was calculated using the B3LYP functional^34,35^ with the larger basis set 6-311+G(d,p)/LANL2DZ. The polarizable continuum model (PCM) with ε = 4 was used for recovering the solvent effect.^36,37^ Both the doublet and quartet spin states of the trimmed compound I (Cpd I) were considered.^38^ Since the aromatic carbon atoms are closer to the heme iron in the crystal structure (PDB: 5TE8),^27^ the three aromatic carbon atoms on the chlorobenzene ring, as well as C1′ and C4, were included in the QC calculations.

### Molecular Dockings

The initial binding modes of MDZ at the productive site of CYP3A4 were obtained using the CCDC GOLD Suite 5.2.2^39^ program. The protein models of CYP3A4 were selected from 11 crystal structures, including 1TQN,^40^ 2V0M,^17^3NXU,^17^3UA1,^41^4D78,^19^4I4G,^42^4K9T,^18^4K9V,^18^4K9W,^18^5TE8,^27^ and 5VC0,^43^ which were prepared in our previous study.^44^ For the docking of the first MDZ molecule, the oxo atom in Cpd I was adopted as the center of a sphere with radius of 15 Å for defining the binding pocket. For the docking of the second MDZ, the centroid of the first docked MDZ was deemed as the center of the binding pocket. The first docked MDZ was then treated as a residue of the protein and the region within 15 Å of the pocket center was defined as the binding pocket for the second MDZ. A total of 50 poses were generated and ranked using ChemScore with the trained parameters for heme-proteins.^45^

The allosteric site was also considered in our docking study using the 4K9T structure. This protein structure was first superimposed to the 1W0F structure,^23^ in which a progesterone molecule was cocrystallized at the allosteric site. Then the coordinates of the progesterone centroid were used to define the docking center for the third MDZ. The residues within 15 Å of the docking center were used for generating docking poses. Fifty poses were generated for ranking.

### Membrane Embedding

In the CYP3A4 crystal structures, the N-terminal residues attached to a membrane were truncated for facilitation of crystallization. In this study, models of the full-length CYP3A4 in a membrane environment were constructed. For the selected top 1 ranked docking complexes, the missing N-terminal region, which is usually comprised of a transmembrane helix (TMH) and a short linking loop, was modeled using the MODELLER program.^46^ The PSIPRED Web server was used to predict the TMH length.^47^ The orientation of TMH was adopted from the orientation of protein in membrane (OPM) database.^48^ The 1-palmitoyl-2-oleoyl-*sn*-glycero-3-phosphocholine (POPC) lipid molecule was used to construct the mimic biomembrane. By using the PDB format, the pretreated CYP3A4-MDZ complex was submitted to CHARMMGUI for the membrane embedding.^49,50^ In the CHARMMGUI settings, the rectangular box was selected with the length in the Z direction determined by the water thickness, which was 17.5 Å. The lengths in the *X* and *Y* directions were set based on the number of lipid components, in which the upper and lower leaflets have 157 and 143 POPC molecules, respectively. The protonation states of the ionizable residues determined in the docking procedure were retained in CHARMMGUI. The sodium and chloride ions were then added to the system to reach the concentration of 0.15 M by the Mote-Carlo method implemented in CHARMMGUI. This resulted in a membrane embedded system containing 300 POPC lipid molecules, 76 sodium ions, 79 chloride ions, and 23 009 TIP3P water molecules, with the size of 104, 104, and 117 Å, in the *X*, *Y*, and *Z* directions, respectively.

### Molecular Dynamics (MD) Simulations

The membrane-embedded CYP3A4-MDZ complex systems were then subject to conventional molecular dynamics (cMD) simulations using the Amber 18 program.^51^ All the MD simulations were executed by the *pmemd.cuda* module^52,53^ with randomized initial atomic velocities. The PDB files generated with CHARMMGUI were converted to the amber format with the *charmmlipid2amber.py* script implemented in Amber 18. The Amber14-SB,^54^ lipid14,^55^ and the general Amber force field (GAFF)^56^ were applied for the protein, POPC membrane, and the ligand molecules, respectively. The geometry of MDZ was optimized at the B3LYP/6-31G* level using Gaussian 09 before calculating the electrostatic potential (ESP) of the molecule and the restrained ESP (RESP)^57^ derived charges were used as the partial atomic charges. The TIP3P^58^ water model was used. The force field parameters for the Cpd I moiety were adopted from Shahrokh’s work.^59^ For each system, an energy optimization procedure was first carried out with the constraints on the heavy atoms of the protein and ligand. In the procedure, the system was optimized thrice using a gradually decreased force constant for each constraint. Then the temperature of the system was raised from 0 to 300 K by two sequential simulations with the restraints on the protein, ligand, and lipid molecules. In the first simulation, the temperature was raised from 0 to 100 K in 200 ps. In the second simulation, the temperature was slowly raised from 100 to 300 K in 1 ns. After the heating step, the system was equilibrated for 50 ns with the extended nonbonded cutoff scheme (*skinnb* = 5 Å in Amber). Thereafter, an unrestrained production run was conducted for 750 ns or 1.5 μs under the NVT ensemble (*T* = 300 K). The production run was performed in a sequential way to obtain multiple 100 ns trajectories, in which the simulation was restarted every 100 ns with the random velocity option (*ig* = −1 in Amber).

In the MD simulations, all the covalent bonds containing hydrogen atoms were restrained using the SHAKE algorithm^60^ and a time step of 2 fs was used. A cutoff of 10 Å was used for the nonbond interactions and the particle mesh Ewald (PME) method^61,62^ was used to handle the long-range electrostatic interaction. A collision frequency of 1.0 ps^–1^ was adopted to control the temperature when the NVT ensemble was applied. The *cpptraj* module^63^ was used for the analysis of trajectories.

### Gaussian Accelerated Molecular Dynamics (GaMD) Simulations

GaMD simulation is a collective-variable (CV) free enhanced sampling method that adds a harmonic boost potential to a system to smoothen the potential energy surface (PES) of the system.^64^ It is possible to perform energetic reweighting of GaMD simulations based the probability distribution along the reaction coordinate or a selected CV.^65^ For each system, the GaMD simulation was proceeded with a 24 ns cMD simulation to collect the potential statistics for deriving the acceleration parameters. Next, an 8 ns equilibration was performed after adding the boost potential. Finally, the productive GaMD simulation was carried out with 15 continual simulations, each of which was run for 100 ns with randomized initial atomic velocities. The “dual-boost” mode was adopted with the reference energy set to the lower bound, i.e., *E* = *V*_max_.^64^ The potential energies of the system were collected every 400 000 steps (800 ps) to calculate the average and standard deviation. The upper limit of the standard deviation of the boost potential (σ_0_) was adjusted to 6.0 kcal/mol.

## Results and Discussion

### Reactivities of the Potential SOMs of MDZ and the Selectivity of the Products

**Figure 1:**
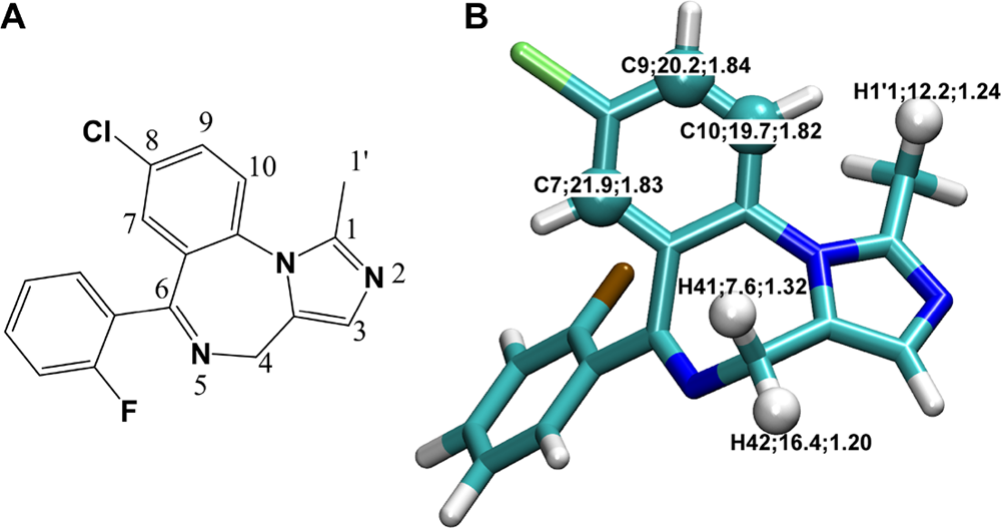
(A) Atom numbering of MDZ. (B) Structure of MDZ. For each potential SOM, the labels separated by the colons represent the site identity, the activation barrier for the *S* = 1/2 state (kcal/mol), and the site–oxo distance (Å) in the corresponding TS structure, respectively.

In this study, we first evaluated the activation barriers for the potential SOMs of MDZ with DFT calculations. These sites are denoted as H1′1, H41 (*pro-R* center), H42 (*pro-S* center), C7, C9, and C10 (Figure 1). For the same site, the predicted activation barriers for the doublet and quartet spin states were found to be similar (Tables S1 and S2). The rankings of the barriers for the potential SOMs are the same for the two spin states, with H41 < H1′1 < H42 < the aromatic sites, i.e, C7, C9, and C10. By including the dispersion corrections to the single point energies, the activation barriers for the aromatic sites reduced significantly (Tables S3 and S4) due to the short distance between the aromatic rings of MDZ and Cpd I.

It has been accepted that the P450 mediated hydroxylation is activated via the hydrogen atom transfer (HAT) mechanism.^66−70^ The experimentally detected hydroxylation sites of MDZ occur at C1′ and C4. The C1′ belongs to a methyl group neighboring the imidazole ring of MDZ, which can stabilize the carbon free radical produced by the HAT process and thereby results in a barrier of only 12.2 kcal/mol for extracting the hydrogen atoms attached to C1′ (see H1′1 in Figure 1). For the C4 attached hydrogen sites (H41 and H42), the activation barriers are 7.6 and 16.4 kcal/mol, respectively, which indicates a high preference for the hydroxylation at the *pro-R* site of C4. The C4 atom is the only sp3 hybrid carbon atom on the benzodiazepine ring of MDZ, in which the delocalized π electrons formed by the HAT process can stabilize the carbon free radical, and vice versus, the formation of the C4 free radical can increase the planarity of the benzodiazepine ring. In addition, the steric hindrance between the chlorobenzene ring and heme impedes H41 to get further close to the oxo moiety, resulting in a large site–oxo distance in ^2^TS_H41_ (1.32 Å for H41 versus 1.20 Å for H42, Figure 1). In this case, the H41–C4 distance is shorter than the H42–C4 distance (1.25 versus 1.33 Å, Table S5), leading to a lower energy of ^2^TS_H41_.

**Figure 2:**
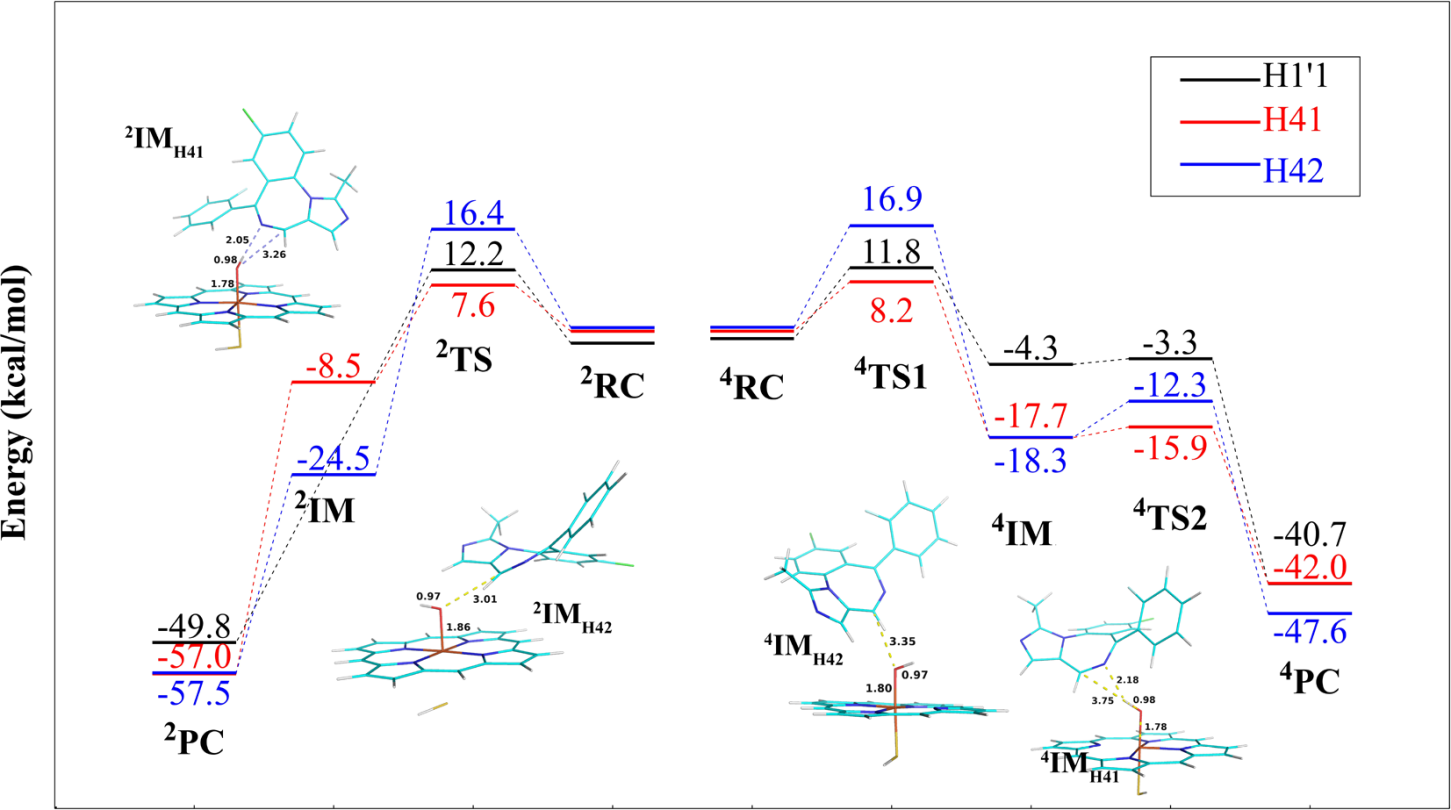
Energy profiles for the hydroxylation at the H1′1 (colored in black), H41 (colored in red), and H42 (colored in blue) sites along the reaction coordinate.

We also calculated the energies for the rebound step for each of the H1′1, H41, and H42 sites (Figure 2 and Table S6). The optimization for the ^2^IM_H1′1_ species, which is derived from the IRC calculation of the ^2^TS_H1′1_ state, resulted in a barrier-free process to the product complex (PC) directly. In the ^2^IM_H41_ species, there is a hydrogen bond between the N5 and the OH atoms and the breaking of this hydrogen bond led to the flipping of MDZ and produces the H42 alcohol. The length of the hydrogen bond between the N5 and the OH atoms in ^4^IM_H41_ is longer than that in ^2^IM_H41_ (2.18 versus 2.05 Å; see Figure 2), which is not able to flip the MDZ molecule and produces the H41 alcohol. We also found that the energies of ^4^PC_H41_ and ^4^PC_H1′1_ are almost the same (Table S5). Therefore, we believe that the C4 hydroxylation product is most likely the racemic 4-OH-MDZ molecule.

It is worthy to note that the H1′1 and H41 sites are located on the same side of the benzodiazepine ring. The root-mean-square deviation (RMSD) values for the MDZ heavy atoms between the H1′1 and H41 species (Figure S1) are 4.4 and 5.3 Å for the RC and TS, respectively. These two sites were also predicted as the joint top-1 ranked sites of metabolism for CYP3A4 by FAME3.^71^ It is likely that they compete each other for accessing to the catalytic center.

### MDZ Binding Modes Predicted by Docking

In the available crystal structure of the CYP3A4-MDZ complex (PDB: 5TE8), the F–F′ helix deeply sinks into the productive site, which disallows the binding of an additional MDZ molecule.^27,28^ Hence, molecular docking was carried out to predict the initial binding modes of the additional MDZ molecule using the 11 crystal structures available from PDB, which also include 5TE8. These structures are socked with various ligands, leading to different volumes of the active site and distinct conformations for the F–F′ loop (Figure S3). The 5TE8 structure has the F–F′ loop sinks deepest into the active site, followed by 1TQN, 3UA1, and 4D78 (Figure S3).

**Figure 3:**
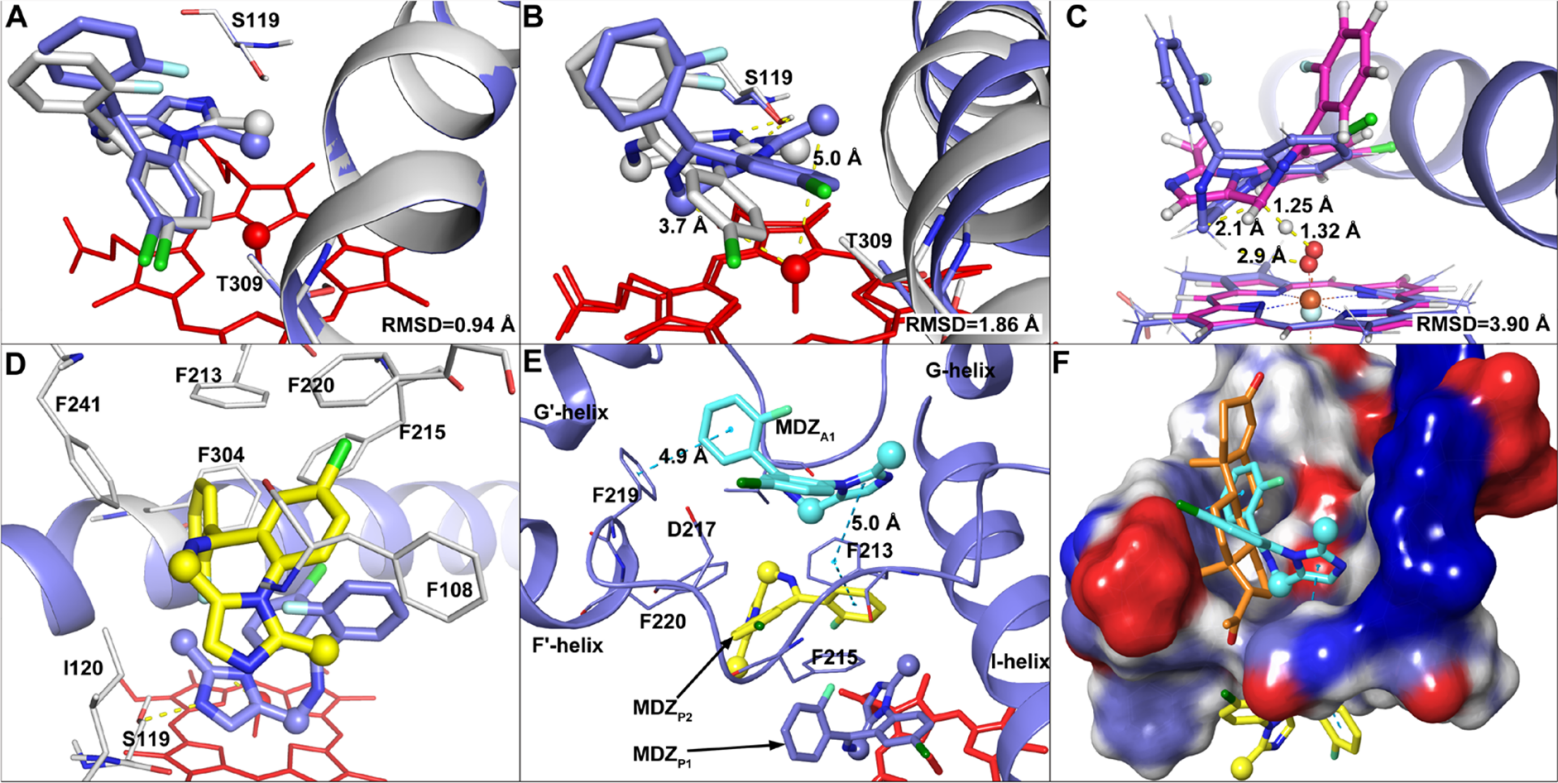
Binding modes of MDZ predicted by molecular docking. (A) Self-docking of MDZ (colored in marine blue) into 5TE8 (colored in white). (B) Comparison of the top-1 ranked poses for the first docked MDZ (denoted as MDZ_P1_) in 4K9T and the MDZ molecule in the crystal structure (PDB: 5TE8, in white). (C) Comparison of the conformations of MDZ in the docked complex (MDZ_P1_, colored in marine blue) and in ^2^TS_H41_ (colored in magenta). (D) Binding mode of the second docked MDZ (denoted as MDZ_P2_, colored in yellow) to 4K9T (colored in marine blue, with the residues interacting with MDZ_P2_ colored in white). (E, F) Conformation of the third docked MDZ molecule (colored in cyan, denoted as “MDZ_A1_”) at the allosteric site. The MDZ_P1_ and MDZ_P2_ molecules are depicted in marine blue and yellow sticks, respectively. The progesterone is colored in orange.

Self-docking of MDZ into 5TE8 was first performed to evaluate the docking protocol, which can reproduce well the experimental binding mode, with the RMSD value of 0.94 Å (Figure 3A). The docking protocol was then applied to the other CYP3A4 crystal structures as well (see Figure S2 and Table S7 for the top-1 ranked poses and scores, respectively). The top-1 ranked pose from the docking using the 4K9T structure has the structure closest to the crystal structure of the CYP3A4-MDZ complex (PDB: 5TE8) with the RMSD of 1.86 Å (Figure 3B). For the first docked MDZ molecule (denoted as MDZ_P1_), the C4-oxo and C1′-oxo distances are 3.7 and 5.0 Å, respectively. Interestingly, the binding pose of MDZ_P1_ is close to that in ^2^TS_H41_, with the RMSD of 3.9 Å, indicating that the binding mode for MDZ_P1_ favors the abstraction of the H41 atom (Figure 3C). We found that a second MDZ molecule (denoted as MDZ_P2_) can also be docked into the productive site of 4K9T with the highest docking score. This molecule interacts with MDZ_P1_, as well as the Phe-cluster on the roof of the CYP3A4 productive site (Figure 3D).

The allosteric site characterized by the progesterone-bound CYP3A4 (PDB: 1W0F) was also considered in this study (Figure 3E, F). The docking experiment indicates that the third MDZ molecule (denoted as MDZ_A1_) lines at the allosteric site deeper than the cocrystallized progesterone (Figure 3F). The MDZ_A1_ molecule mainly forms π–π interactions with Phe219 and Phe213. In a recent study, Phe213 was found to play important roles in the allosteric effect caused by progesterone and carbamazepine.^72^ In our docking experiments, Phe213 was found to interact with the MDZ_P2_ and MDZ_A1_ molecules.

### Conformational Dynamics in cMD Simulations

To investigate the dynamics of the homotropic binding of MDZ to CYP3A4, we performed MD simulations for the docked complexes in aqueous solution. According to the docking results, six systems were considered and subject to the cMD simulations (Table 1).

**Table 1:** Scenarios for the MD Simulations

system	ligand (s)^a^	cMD	cMD2^b^	GaMD
P1	MDZ_P1_	1.5 μs		1.5 μs
P1P2	MDZ_P1_ + MDZ_P2_	1.5 μs		1.5 μs
A1	MDZ_A1_	750 ns	1.0 μs	
A1P1	MDZ_A1_ + MDZ_P1_	750 ns	1.0 μs	
A1P2	MDZ_A1_ + MDZ_P2_	750 ns	1.0 μs	
A1P1P2	MDZ_A1_ + MDZ_P1_ + MDZ_P2_	750 ns	1.0 μs	

a See Figure 3 for the position(s) of the MDZ molecule(s) in CYP3A4 (4K9T).

b MD simulations for the system without a membrane environment and TMH.

**Figure 4:**
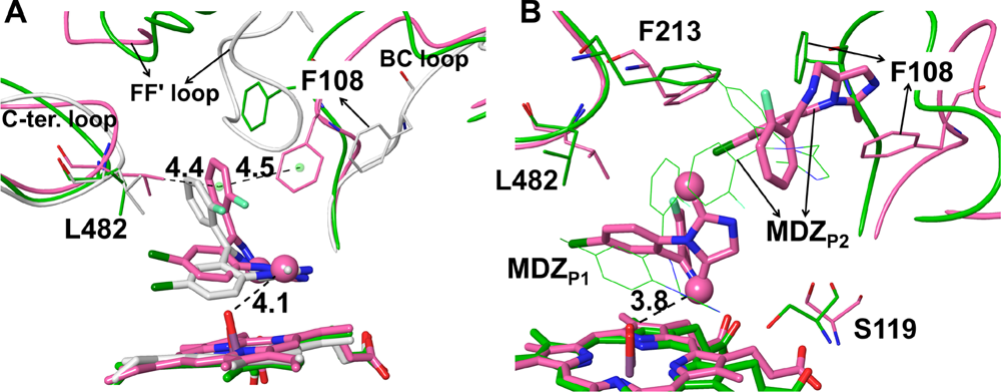
(A) Comparison of a representative snapshot in the major cluster (colored in pink), the initial structure (colored in green) for the P1 system, and the crystal structure of 5TE8 (colored in light gray). (B) Comparison of a representative snapshot in the major cluster (colored in pink) and the initial structure (colored in green) for the P1P2 system.

The representative structures in the major clusters for the P1 and P1P2 systems are presented in Figure 4. The MDZ_P1_ molecule in the major cluster of P1 is well superimposed to that in the 5TE8 structure, with the RMSD of 1.36 Å. In 5TE8, the fluorophenyl ring of MDZ interacts with the collapsed backbones of the F–F′ loop and F′-helix. In our MD simulation, the F–F′ loop did not sink into the productive site and the fluorophenyl ring of MDZ interacted with Phe108 on the BC loop, which shrank the productive site significantly to allow the binding of only one MDZ (Figure 4A). The N2 atom of MDZ_P1_ was found to form a hydrogen bond with the OH group of Ser119 in 55% of the frames (Table S8). For the P1P2 system, the existence of the second MDZ, MDZ_P2_, affected the dynamics of MDZ_P1_ and resulted in the C1′ atom moving away from the Cpd I oxo atom. The MDZ_P2_ forced the B–C loop to move outward and broke the hydrogen bond between S119 and MDZ_P1_. Phe108 in the P1P2 system moved into the productive site and interacts with the imidazole ring of MDZ_P2_. Its neigboring residue, Gly109, was found to form a hydrogen bond with the N2 atom of MDZ_P2_ in 38% of the MD frames. The N2 atom of MDZ_P2_ was also found to form a hydrogen bond with Ser119 in 14% of the frames. In summary, the unbiased MD simulation of P1 can successfully reproduce the binding mode of MDZ_P1_ in 5TE8. The conformational change of MDZ_P1_ in the MD simulation of the P1P2 system is also in line with the experimental observations that the hydroxylation is more favorable at C4 than C1′ at a higher concentration of MDZ.^26^

In the P1 system, although the C4-oxo distance was shorter than the C1′-oxo distance in the initial structure, the C4-oxo distance became longer than C1′-oxo (Figure S4). In contrast, the C1′-oxo distance is much longer than C4-oxo in the P1P2 system (Figure S4). From the RMSD values of the MDZ molecules, we found that MDZ_P1_ in P1P2 was less deviated from the docked pose than that in P1 (Figure S5A). The RMSF analysis indicates that the backbone atoms of the productive site residues are more fluctuating in P1P2 than in P1, indicating that the movement of MDZ_P2_ can induce significant conformational change of the protein, especially for the B–C and F–F′ loops (Figure S5B). The high RMSF values in the B–C and F–F′ loops are also in line with the conformations observed in the major cluster structures of P1P2 (Figure 4B).

**Figure 5:**
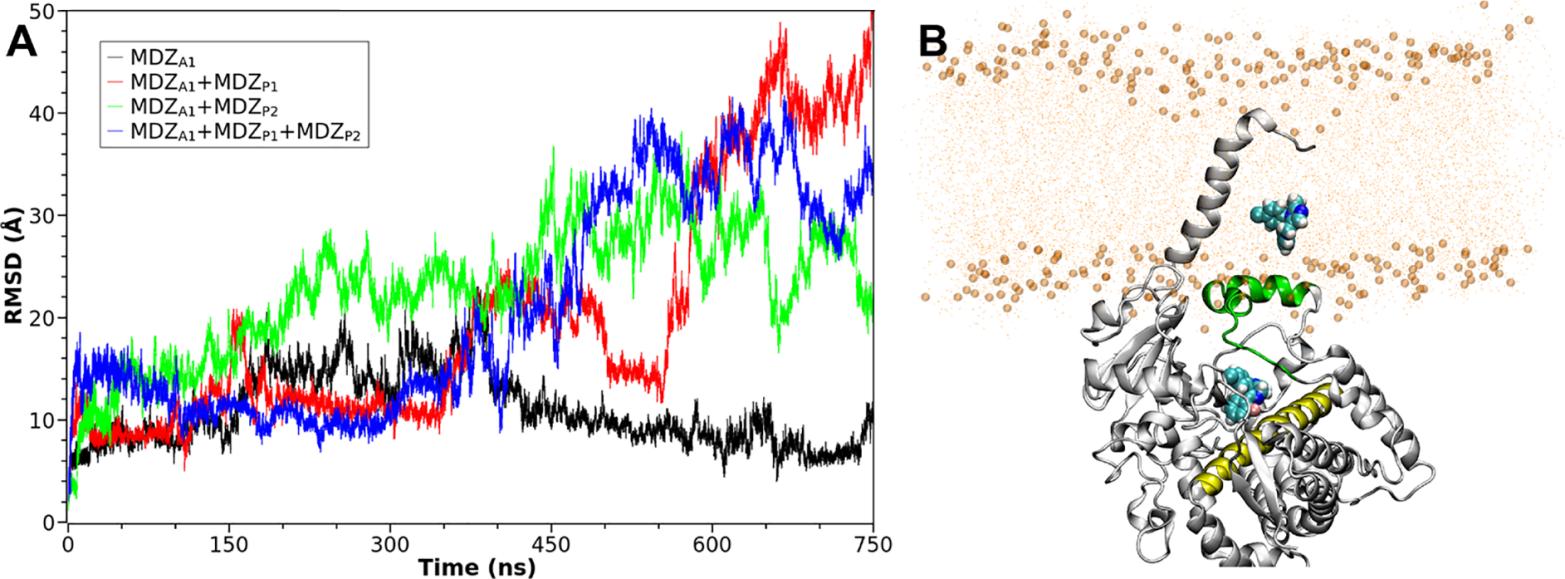
(A) Time evolution of the RMSDs for MDZ_A1_ in the A1 (colored in black), A1P1 (colored in red), A1P2 (colored in green), and A1P1P2 (colored in blue) systems, respectively. (B) Average structure for the A1P2 system, where the I-helix, and F′- and *G*′-helices are colored in yellow and green, respectively, and the MDZ molecules are cyan spheres.

Interestingly, our MD simulations indicate that MDZ_A1_ in the A1, A1P1, A1P2, A1P1P2 systems was unstable (Figure 5). Although MDZ_A1_ was found to fit well to the allosteric site in the molecular dockings, it went deeply into the membrane in these systems, as observed from the MD simulations (Figure 5B). Since the 1W0F structure, in which a steroid molecule binds to the allosteric site, was crystalyzed without a membrane, we also performed MD simulations for the A1, A1P1, A1P2, and A1P1P2 systems without a membrane environment (Figure S6). We found that MDZ_A1_ was still unstable after long-time MD simulations and could stay at the allosteric site for only ∼600 ns, as observed from the simulation of the A1P1P2 system (see Figure S7 for its conformations before and after 600 ns). Additionally, we also conducted four more cMD simulations for the remote allosteric site starting from the 1W0F structure with and without membrane (Table S9). Unlike in the 4K9T systems, the ligands in the 1W0F systems with membrane were found to be more stable in the beginning of the simulations (Figures S8 and S9). Though the effects of the membrane on the dynamics of the ligands are different for 4K9T and 1W0F,^73,74^ both MDZ and progesterone were found to be detaching away from this site during the MD simulations.

By comparing the dynamic behaviors of MDZ at the productive and allosteric site, we conclude that the homotropic MDZ binding occurs most likely at the productive site. The binding of the second MDZ molecule alters the dynamics of the first MDZ molecule, which can favor the hydroxylation at C4.

### Energy Profiles Derived from GaMD Simulations

**Figure 6:**
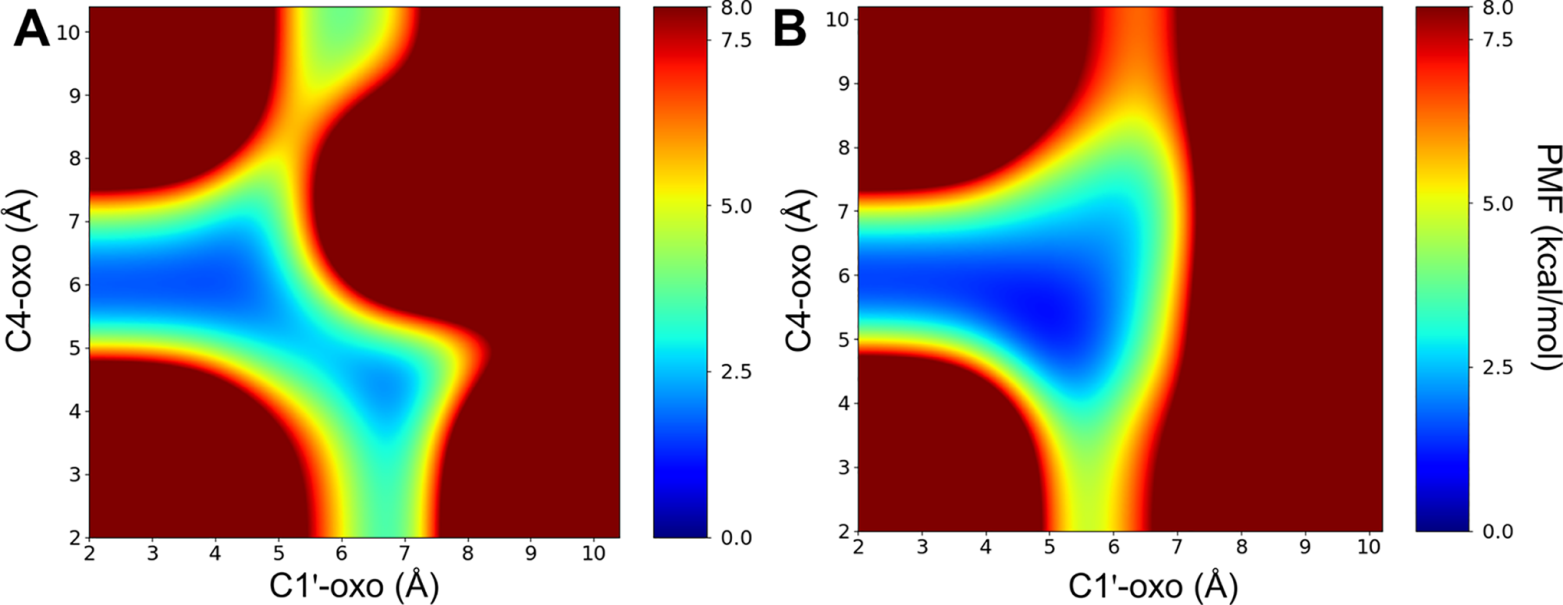
Reweighted PMFs with respect to the selected CVs (the C1′-oxo and C4-oxo distances) for the P1 (A) and P1P2 (B) systems, respectively.

To further investigate the dynamics of the homotropic MDZ binding, we preformed GaMD simulations for the P1 and P1P2 systems (Figure 6). For each simulation, a CV-free GaMD boost potential was added to the system to enhance the conformational sampling of the system.^75^ For the analysis of the result, the C1′-oxo and C4-oxo distances for MDZ_P1_ were selected as CVs for reweighting. Our analysis indicates that there exist two major local minimums on the reweighted potential of mean force (PMF) surface for the P1 system, which reflects that the hydroxylation favors at C1′ and C4 (Figure 6A). In the GaMD simulation, the movement of C4 close to the oxo moiety of Cpd I can be observed (Figure S10A). This is in contrast to the cMD simulation result, where the hydroxylation was found to favor only at C1′. For the P1P2 system, the local minimum was found to span less on the region corresponding to shorter C4-oxo distances. Meanwhile, the local minimum region located at the C1′-oxo and C4-oxo distances ranging from 5 to 6 Å increased significantly (Figure 6B), suggesting that C4 is still a competitive site for hydroxylation with the binding of the second MDZ. (Figure S10B).

## Conclusions

The homotropic cooperativity of CYP3A4 has been observed for many substrates, such as testosterone and aflatoxin B1.^11^ In this work, we focused on the homotropic cooperativity of MDZ metabolism, in which the product distribution is dependent on the substrate concentration and the hydroxylation product configuration.

QC calculations were first conducted for both the doublet and quartet spin states. The abstraction of the H41 atom at C4 was found to have the lowest activation barrier. The difference in the activation energy for the H11′ and H41 sites is only 3–4 kcal/mol. Additionally, by comparing the QC optimized TS structures, it can be deduced that there exists competition for the hydroxylation between C1’ and C4. The QC calculation results also imply that the 4-OH-MDZ is probably in a racemic form. The MD simulations revealed that the binding of MDZ at the peripheral allosteric site is not stable. The simulation results for the P1 and P1P2 systems also indicate that the hydroxylation favors at C1′- and C4, respectively. It was found that the mobility of the MDZ_P1_ molecule in P1P2 is lower than that in P1. The GaMD derived PMF surfaces indicate that there exist two local minimums with respect to the C1′-oxo and C4-oxo distances for MDZ_P1_ in the P1 system and only one expanded local minimum in the P1P2 system, in which the C1′-oxo and C4-oxo distances are almost the same. Our studies thus provide structural and dynamical insights into the cooperative binding of multiple MDZ molecules to CYP3A4 and are useful for understanding the P450 cooperativity in general.

### Data and Software Availability

Other data and scripts can be obtained from the author upon a appropriate request. The software used in this study includes Gaussian (commercial, see https://gaussian.com/glossary/g09/), Schrödinger Suite (commercial, a free trial version can be obtained from https://www.schrodinger.com/freemaestro), CCDC GOLD suite (commercial, see https://www.ccdc.cam.ac.uk/solutions/csd-discovery/components/gold/), Amber (commercial for the GPU unit, see http://ambermd.org/), VMD (open source, see https://www.ks.uiuc.edu/Research/vmd/vmd-1.9.3/), Pymol (open source, see https://github.com/schrodinger/pymol-open-source), and Modeller (open source, see https://salilab.org/modeller/).

## Supporting Information Available

All the QC optimized geometries and energies (PDF)
