# Supplementary material for: Homotropic Cooperativity of Midazolam Metabolism by Cytochrome P450 3A4: Insight from Computational Studies
Source: J Chem Inf Model. 2021 Apr 22;61(5):2418–26. doi: 10.1021/acs.jcim.1c00266 (PMC8278384; doi:10.1021/acs.jcim.1c00266)
Supplement: Supplementary file 1 — ci1c00266_si_001.pdf [file ci1c00266_si_001.pdf]

Supporting Information

Homotropic Cooperativity of Midazolam Metabolism by Cytochrome P450 3A4: Insight from Computational Studies

Junhao Li[1], Yue Chen[2], Yun Tang[2], Weihua Li[2,*], Yaoquan Tu[1,*]

[1] *Department of Theoretical Chemistry and Biology, School of Engineering Sciences in Chemistry, Biotechnology and Health (CBH), KTH Royal Institute of Technology, SE-106 91 Stockholm, Sweden*

[2] *Shanghai Key Laboratory of New Drug Design, School of Pharmacy, East China University of Science and Technology, Shanghai 200237, China*

* Corresponding authors, Tel: +46-8-7909645; Fax: +46-8-55378590; E-mail: yaoquan@kth.se (Y. Tu) or whli@ecust.edu.cn (W. Li)

Table S1. Energies for the activation species in the doublet spin state, calculated using the B3LYP functional

Sites	SP_TS[a] (a.u.)	ZPE_TS[b] (a.u.)	SP_RC[a] (a.u.)	ZPE_RC[b] (a.u.)	EA[c] (kcal/mol)	ZPE_EA[d] (kcal/mol)
H1'1	-3004.40374341825	0.554273	-3004.42890341552	0.560057	15.8	12.2
H41	-3004.40768437949	0.554172	-3004.42560548918	0.559933	11.2	7.6
H42	-3004.39129708656	0.552693	-3004.42458059311	0.559852	20.9	16.4
C7	-3004.39067623393	0.558684	-3004.42670939393	0.559762	22.6	21.9
C9	-3004.39449632619	0.558891	-3004.42798871627	0.560219	21.0	20.2
C10	-3004.39381183182	0.559587	-3004.42555543461	0.559964	19.9	19.7

[a.] SP energy calculated using 6-311+g(d,p)/LANL2DZ/PCM, see Method for details

[b.] ZPE energy calculated using 6-31g(d)/LANL2DZ

[c.] activation barrier

[d.] activation barrier with ZPE correction

Table S2. Energies for the activation species in the quartet spin state, calculated using the B3LYP functional

Sites	SP_TS[a] (a.u.)	ZPE_TS[b] (a.u.)	SP_RC[a] (a.u.)	ZPE_RC[b] (a.u.)	EA[c] (kcal/mol)	ZPE_EA[d] (kcal/mol)
H1'1	-3004.40372807611	0.554821	-3004.42783635510	0.560195	15.1	11.8
H41	-3004.40688878505	0.554263	-3004.42560600985	0.559954	11.7	8.2
H42	-3004.39118787377	0.553528	-3004.42457298354	0.559923	20.9	16.9
C7	-3004.39381641371	0.559427	-3004.42782105195	0.560237	21.3	20.8
C9	-3004.39230725333	0.559330	-3004.42557132908	0.560030	20.9	20.4
C10	-3004.38844393357	0.559281	-3004.42651197371	0.559814	23.9	23.6

[a. b. c. d.] See the footnotes in Table S1.

Table S3. Energies for the activation species in the doublet spin state, calculated using the B3LYP functional with the D3 dispersion energy correction

Sites	SP_TS[a] (a.u.)	ZPE_TS[b] (a.u.)	SP_RC[a] (a.u.)	ZPE_RC[b] (a.u.)	EA[c] (kcal/mol)	ZPE_EA[d] (kcal/mol)
H1'1	-3004.62957927516	0.554273	-3004.64803941841	0.560057	11.6	8.0
H41	-3004.64147149676	0.554172	-3004.65316222064	0.559933	7.3	3.7
H42	-3004.62116659662	0.552693	-3004.64838455692	0.559852	17.1	12.6
C7	-3004.62992791122	0.558684	-3004.65018078071	0.559762	12.7	12.0
C9	-3004.62681629111	0.558891	-3004.65401726175	0.560219	17.1	16.2
C10	-3004.63228381416	0.559587	-3004.65306188923	0.559964	13.0	12.8

a. b. c. d. See the footnotes in Table S1.

Table S4. Energies for the activation species in the quartet spin state calculated using the B3LYP functional with the D3 dispersion energy correction

Sites	SP_TS[a] (a.u.)	ZPE_TS[b] (a.u.)	SP_RC[a] (a.u.)	ZPE_RC[b] (a.u.)	EA[c] (kcal/mol)	ZPE_EA[d] (kcal/mol)
H1'1	-3004.62962044663	0.554821	-3004.65367825866	0.560195	15.1	11.7
H41	-3004.64014424116	0.554263	-3004.65317157754	0.559954	8.2	4.6
H42	-3004.62148446853	0.553528	-3004.64821456828	0.559923	16.8	12.8
C7	-3004.62634040327	0.559427	-3004.65428441045	0.560237	17.5	17.0
C9	-3004.63029038131	0.559330	-3004.65308147173	0.560030	14.3	13.9
C10	-3004.62826720510	0.559281	-3004.65028619789	0.559814	13.8	13.5

a. b. c. d. See the footnotes in Table S1.

Table S5. Energies (in a.u.) for the rebound step species, calculated at the B3LYP/6-311+g(d,p)/LANL2DZ/PCM level

	Sites	SP_IM	ZPE_IM	SP_TS2	ZPE_TS2	SP_PC	ZPE_PC
S	H1'1	\	\	\	\	-3004.5130881178	0.564821
=	H41	-3004.4379849740	0.558830	\	\	-3004.5194298804	0.562886
1/2	H42	-3004.4616402837	0.557889	\	\	-3004.5191334799	0.562846
S	H1'1	-3004.4328448930	0.558394	-3004.4307765599	0.557814	-3004.4946643940	0.562092

=	H41	-3004.4522972712	0.558370	-3004.4477867593	0.556760	-3004.4940421273	0.561533
3/2	H42	-3004.4520298675	0.558183	-3004.4408364632	0.556631	-3004.5007078454	0.560222

Table S6. Geometrical features for the RC and TS species

	Sites	RC		TS		
		Site-OE (Å)[a]	Site-OE-FE (°)[a]	Site-OE (Å)[a]	Site-OE-FE (°)[a]	Site-$C_{abstracted}$ (Å)[a]
doublet	H1'1	2.41	140.1	1.24	119.2	1.29
	H41	2.44	141.2	1.32	124.8	1.25
	H42	2.46	135.9	1.20	122.3	1.33
	C7	3.58	155.3	1.83	142.1	\
	C9	3.84	142.6	1.84	120.4	\
	C10	3.83	121.3	1.82	127.4	\
quartet	H1'1	2.59	133.1	1.24	123.8	1.32
	H41	2.45	143.7	1.29	126.3	1.28
	H42	2.46	135.5	1.23	129.3	1.33
	C7	3.58	157.4	1.81	145.8	\
	C9	3.84	140.6	1.82	132.2	\
	C10	3.86	122.6	1.80	134.5	\

[a]. Geometrical features are illustrated using the following examples:

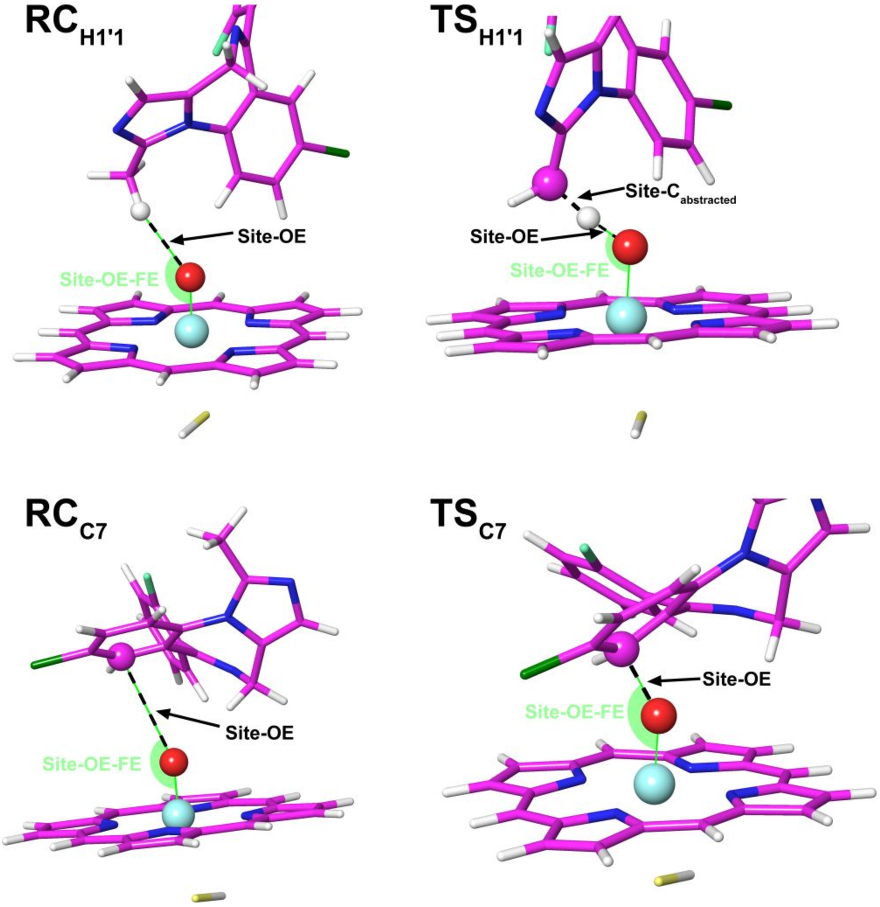

Table S7. Top-1 ranked scores (GOLD ChemScore)

PDB	1st MDZ	2nd MDZ	allosteric MDZ
1TQN	36.42	\	\
2V0M	37.52	\	\
3NXU	38.10	\	\
3UA1	35.97	\	\
4D78	37.83	\	\
4I4G	38.74	\	\
4K9T	39.33	42.63	28.79
4K9V	36.88	\	\
4K9W	38.05	\	\
5TE8	39.70	\	\
5VC0	38.62	\	\

Table S8. Hydrogen bonding analysis for the MD simulated system

Systems	#Acceptor	DonorH	Donor	Frames	Fraction (%)	Avg. Dist.[a]	Avg. Ang.[b]
P1 in cMD	MDZ_{P1}@N2	SER119@HG	SER119@OG	16683	55.6%	2.97	156.4
	MDZ_{P1}@N5	ARG105@HH11	ARG105@NH1	76	0.3%	3.22	134.8
	MDZ_{P1}@N2	PHE108@H	PHE108@N	10	0.0%	3.20	163.8
	MDZ_{P1}@N2	ARG105@HH12	ARG105@NH1	9	0.0%	3.09	147.8
	MDZ_{P1}@N5	ARG105@HH12	ARG105@NH1	9	0.0%	3.16	128.4
	MDZ_{P1}@N2	ARG105@HH21	ARG105@NH2	4	0.0%	3.31	130.9
	MDZ_{P1}@N2	ARG105@HE	ARG105@NE	1	0.0%	3.07	130.4
P1P2 in cMD	MDZ_{P2}@N2	GLY109@H	GLY109@N	11536	38.5%	3.23	142.3
	MDZ_{P2}@N2	SER119@HG	SER119@OG	4250	14.2%	2.90	162.4
	MDZ_{P1}@N5	ARG105@HH12	ARG105@NH1	345	1.2%	3.26	146.9
	MDZ_{P1}@N2	SER119@HG	SER119@OG	239	0.8%	3.04	155.0
	MDZ_{P2}@N2	PHE108@H	PHE108@N	205	0.7%	3.16	159.8
	MDZ_{P2}@N2	ARG105@HH21	ARG105@NH2	165	0.6%	3.05	142.6
	MDZ_{P2}@N2	ARG105@HE	ARG105@NE	83	0.3%	3.20	136.6
	MDZ_{P1}@N5	SER119@HG	SER119@OG	25	0.1%	3.26	162.9
	MDZ_{P2}@N2	ARG105@HH12	ARG105@NH1	13	0.0%	3.18	142.6

a. Average distance between the donor and acceptor atoms (Å)

b. Average angle formed by the acceptor, donor hydrogen, and donor atoms (°)

Table S9. cMD simulations using 1W0F as a starting structure for studying the allosteric site

System	Ligand	membrane embedding	number of atoms	Simulation time
1W0F_STR	progesterone	no	59231	1000 ns
1W0F_MDZ	midazolam	no	59163	1000 ns
1W0F_STR_mem	progesterone	yes	135960	1000 ns
1W0F_MDZ_mem	midazolam	yes	135826	250 ns

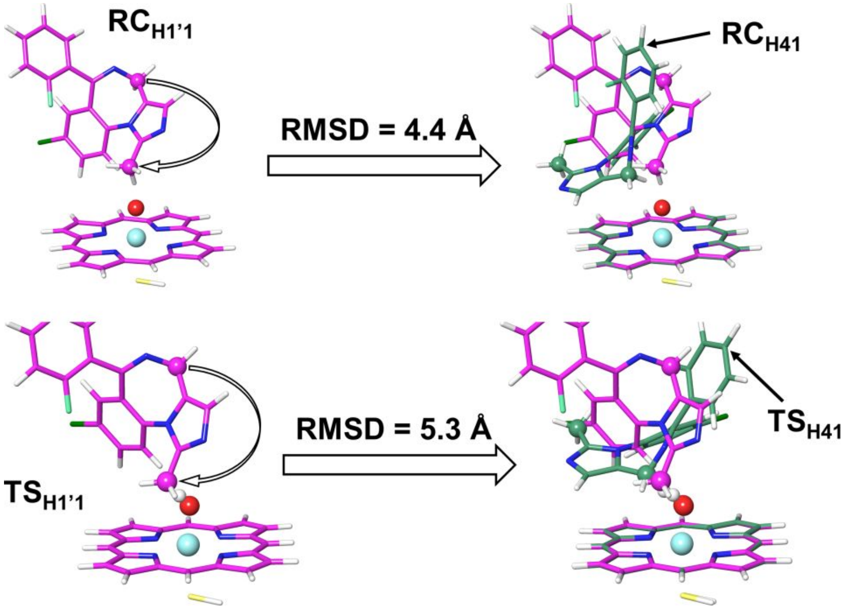

Figure S1. Comparison of the doublet geometries between RCH1'1 and RCH41, and between TSH1'1 and TSH41. The iron, oxo, C4 and C1' atoms are depicted in spheres. The RMSD value is for the MDZ heavy atoms between the H1'1 and H41 species.

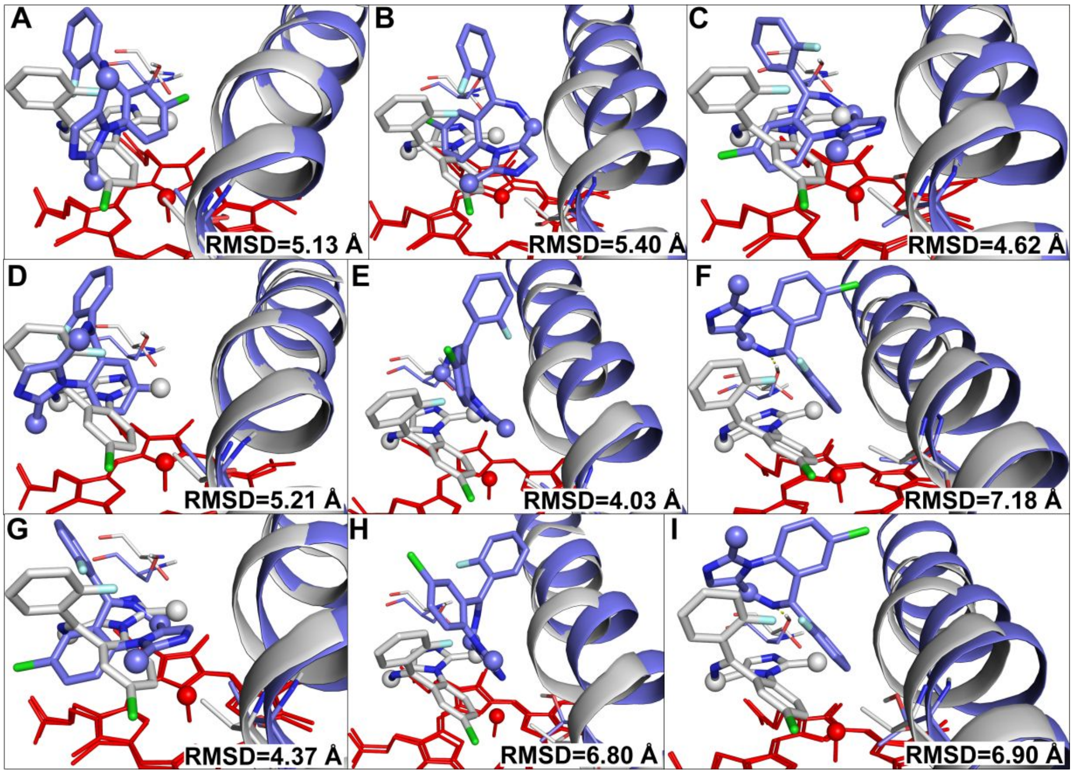

Figure S2. Comparison of the ligand binding pose in 5TE8 (colored in white) and the top-1 ranked poses (colored in marine blue) obtained from the dockings using different crystal structures: (A) 1TQN; (B) 2V0M; (C) 4K9W; (D) 3UA1; (E) 4D78; (F) 4I4G; (G) 4K9V; (H) 3NXU; (I) 5VC0. The root mean square deviation (RMSD) between the docked and 5TE8 midazolam molecules is also shown.

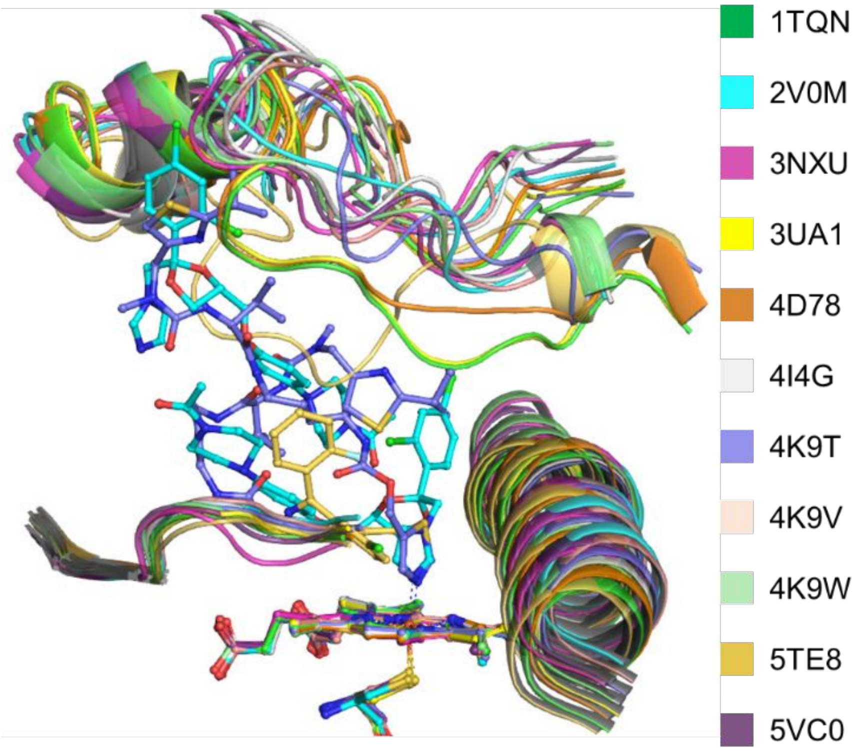

Figure S3. Superposition of the binding pockets of the 11 selected CYP3A4 structures. Shown in the figure are only the F-G cassette, I-helix, and K-L loop. The heme moiety and its ligated Cys442, and the socked ligands in 2V0M, 4K9T, and 5TE8 were shown in sticks.

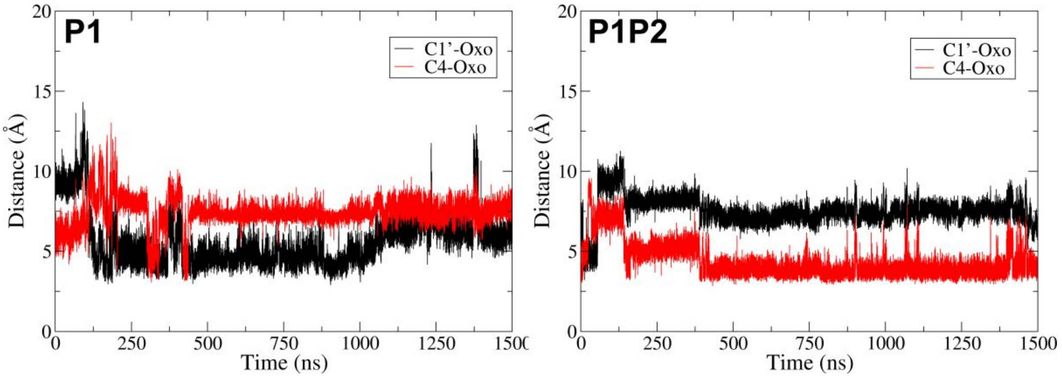

Figure S4. Plots of the time evolution of the C1'-oxo and C4-oxo distances for MDZ_{P1} in the P1 and P1P2 systems

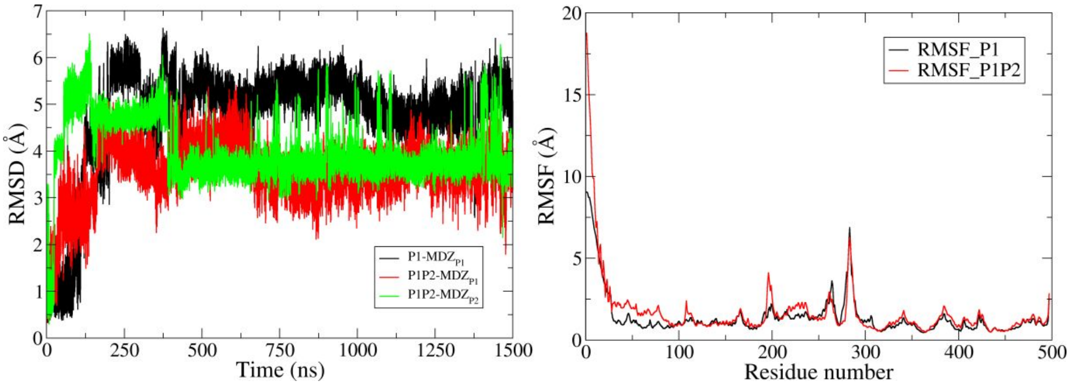

Figure S5. RMSD evolutions of the MDZ molecules (left) and RMSF values for the protein C_α atoms (right) in the P1 and P1P2 systems.

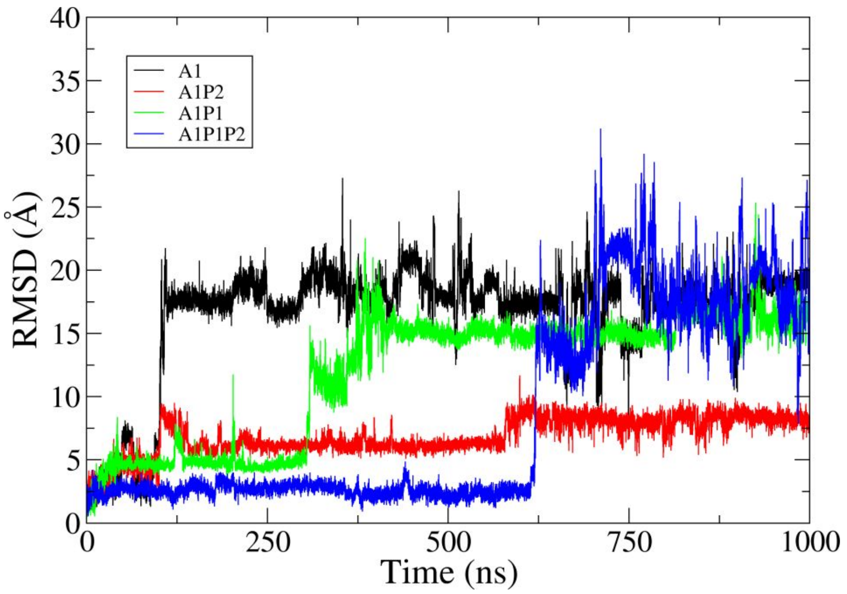

Figure S6. RMSD of MDZ_{A1} in the A1, A1P1, A1P2, and A1P1P2 systems without a membrane environment.

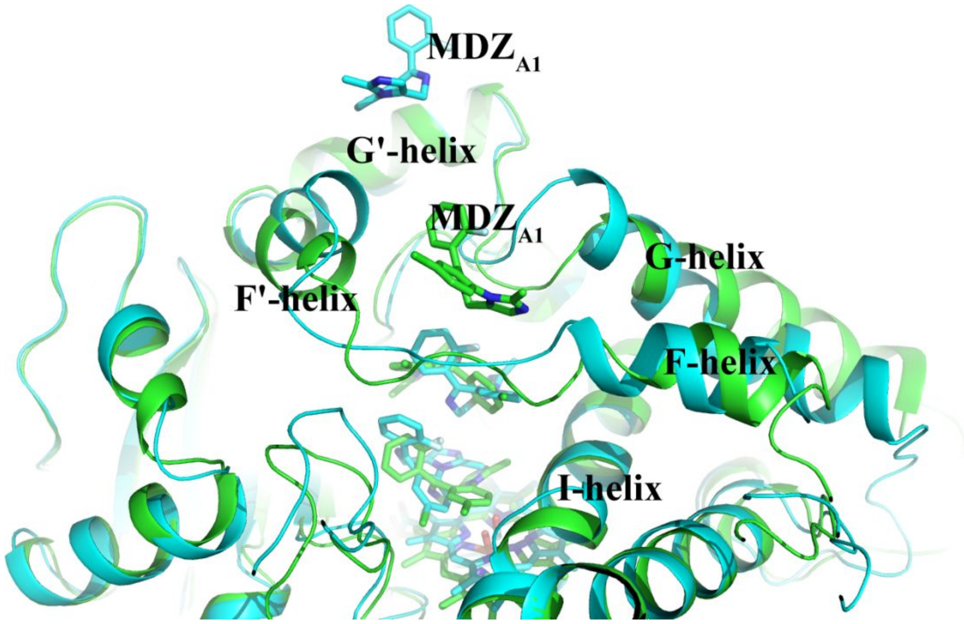

Figure S7. Comparison of the initial structure (colored in green) and the snapshot after 600 ns of the MD simulation (colored in cyan) for the A1P1P2 system without a membrane environment.

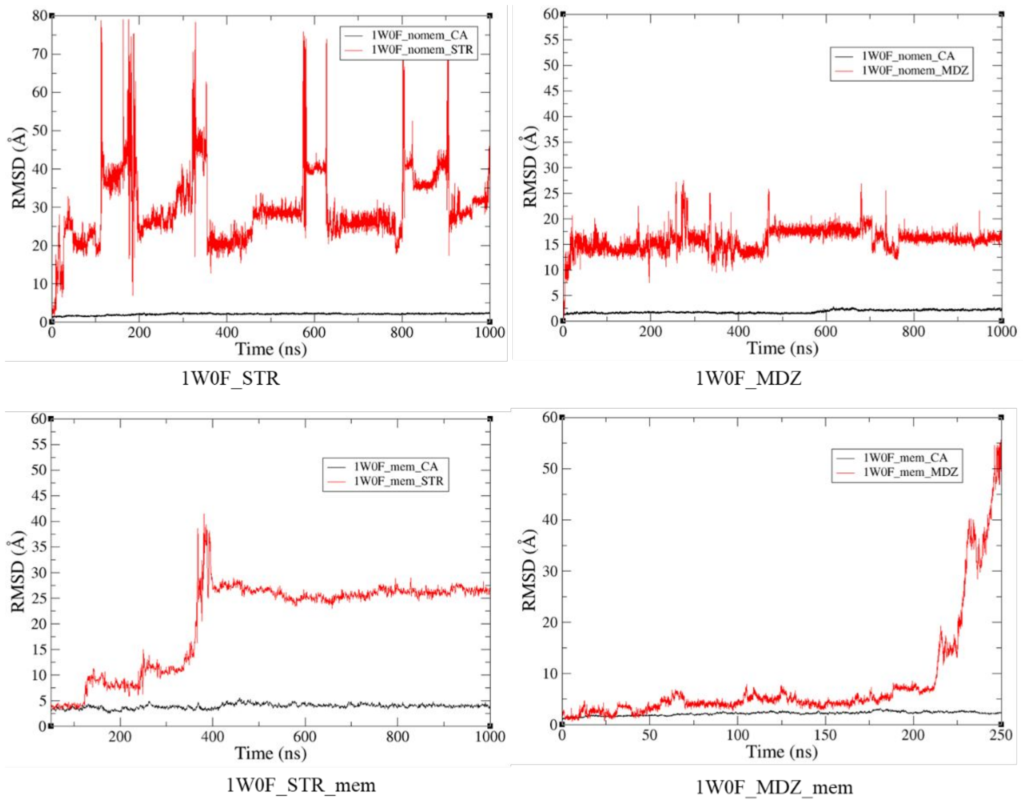

Figure S8. RMSD values of the Cα atoms and ligands (MDZ, and STR) in the four 1W0F systems (see **Table S9**).

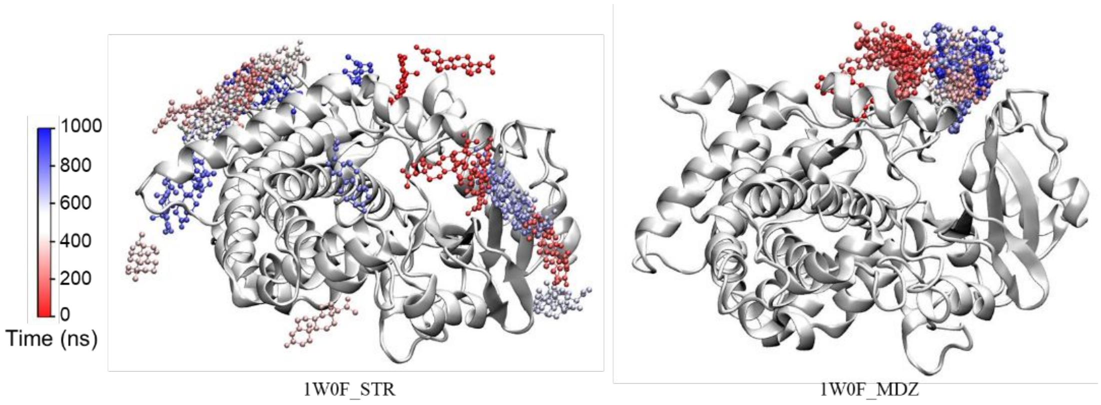

Figure S9. Distributions of STR and MDZ in the 1W0F structure during the simulations without membrane bound.

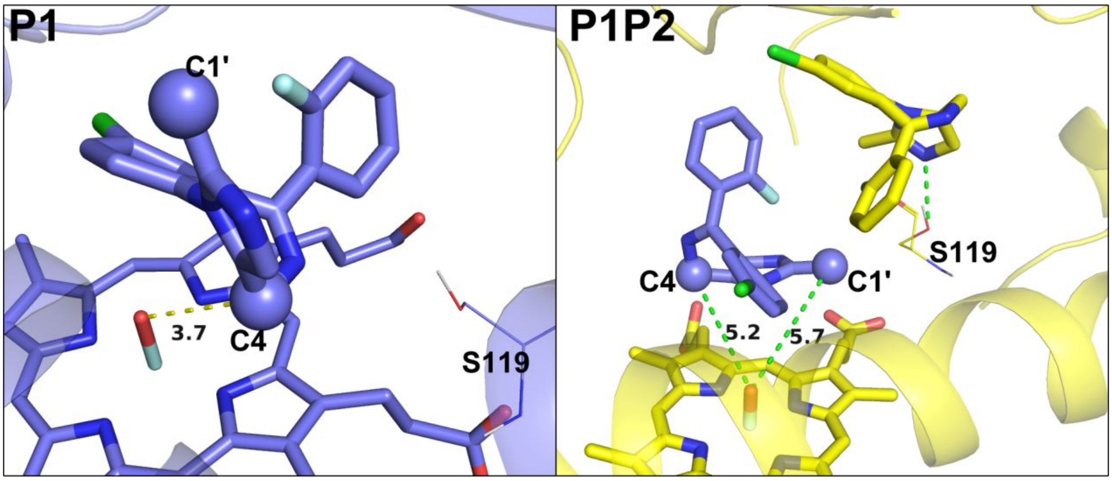

Figure S10. Representative snapshots for the P1 and P1P2 systems in the GaMD simulations. MDZ_{P1} and MDZ_{P2} are colored in marine blue and yellow, respectively.

Coordinates for the optimized species

$^{2}RC_{H1'1}$

H	1.248071	-0.063256	-6.045931
S	2.196296	-0.386744	-5.142079
N	2.138519	-1.126508	-2.197047
C	1.974048	-2.484662	-2.105057
C	3.277790	-0.836552	-1.486130
C	3.034754	-3.066862	-1.318890
C	3.847679	-2.043501	-0.938631
C	3.803753	0.433834	-1.304596
H	4.716245	0.516167	-0.722784
C	3.253157	1.612480	-1.796407
N	2.109670	1.699744	-2.543096
C	1.937357	3.031551	-2.810335
C	3.000216	3.806385	-2.217579
C	3.820663	2.922397	-1.587391
C	0.896999	3.572004	-3.556622
H	0.883235	4.649757	-3.683754
C	-0.125993	2.847651	-4.151885
C	-1.198656	3.432087	-4.919384
C	-1.396687	1.194633	-4.811279
N	-0.261609	1.483469	-4.102528
C	-1.989766	2.403888	-5.329068
C	-1.909177	-0.081859	-5.022596
H	-2.820455	-0.160800	-5.606705
C	-1.355558	-1.258869	-4.546534
C	-1.909825	-2.572787	-4.762715
N	-0.214061	-1.343053	-3.779815
C	-0.060919	-2.679276	-3.482315
C	0.944242	-3.210544	-2.691763
H	0.937121	-4.283641	-2.530385
Fe	0.862535	0.199551	-3.031415
C	-1.110068	-3.451398	-4.101408
O	-0.054294	0.334896	-1.692575
C	-1.213275	-0.697559	3.778673
C	-0.154994	-1.578816	4.052714
H	-0.048855	-1.991579	5.049263
C	0.753813	-1.924406	3.061691
Cl	2.060252	-3.041552	3.439563
C	0.641905	-1.404569	1.773328
H	1.347979	-1.686514	1.000473
C	-0.404250	-0.534378	1.483700
H	-0.508094	-0.160280	0.471045
C	-1.328744	-0.168183	2.471546
N	-2.381327	0.723919	2.145592
C	-2.356581	1.882793	1.378391
C	-1.121581	2.495215	0.799997
H	-1.345801	3.540571	0.579715
H	-0.281621	2.452472	1.501171
H	-0.806252	2.008029	-0.129489
N	-3.563321	2.398481	1.272948
C	-4.404913	1.567448	1.979712
H	-5.464360	1.769402	2.062037
C	-3.709762	0.527598	2.531495
C	-4.049841	-0.591659	3.453075
H	-3.709560	-1.554817	3.039334
H	-5.134744	-0.647371	3.569925
N	-3.454695	-0.364728	4.776874
C	-2.176463	-0.393755	4.887759
C	-1.629109	-0.184738	6.265051
C	-2.334684	-0.672857	7.377853
H	-3.256898	-1.211753	7.190169
C	-1.874427	-0.470383	8.675834
H	-2.434979	-0.867109	9.516523
C	-0.691451	0.241814	8.893110
H	-0.325323	0.404099	9.902447
C	0.022616	0.752450	7.810420
H	0.935176	1.323954	7.940723
C	-0.455709	0.534502	6.523338
F	0.242214	1.064703	5.495080
H	4.749761	-2.084325	-0.343021
H	3.132699	-4.122079	-1.101919
H	4.727360	3.119928	-1.031370
H	3.092332	4.881940	-2.288015
H	-2.800575	-2.776070	-5.342030
H	-1.206773	-4.526282	-4.026714
H	-2.892203	2.441631	-5.924397
H	-1.315532	4.490823	-5.108139

$^{2}TS_{H1'1}$

H	1.861153	2.863566	-43.981344
S	2.760139	2.526066	-43.033879
N	2.513625	1.303258	-40.310225
C	2.448733	-0.059508	-40.456915
C	3.542517	1.542440	-39.435423
C	3.473083	-0.695469	-39.663872
C	4.154045	0.299835	-39.029948
C	3.937111	2.797710	-38.988818
H	4.777301	2.838020	-38.302636
C	3.360801	4.007620	-39.358427

N	2.292942	4.150708	-40.206866
C	2.108769	5.502467	-40.348945
C	3.067176	6.230051	-39.551427
C	3.843580	5.300389	-38.932263
C	1.141317	6.096031	-41.150681
H	1.100938	7.180607	-41.164678
C	0.211578	5.412619	-41.924494
C	-0.821779	6.043622	-42.708381
C	-0.936410	3.800696	-42.861523
N	0.130278	4.048113	-42.036719
C	-1.538646	5.041565	-43.286468
C	-1.378635	2.542477	-43.259591
H	-2.242752	2.508717	-43.915723
C	-0.804224	1.330649	-42.903733
C	-1.266296	0.035732	-43.350475
N	0.288154	1.189633	-42.086077
C	0.518811	-0.160147	-41.992208
C	1.522361	-0.744799	-41.234656
H	1.590160	-1.828029	-41.247533
Fe	1.250346	2.678961	-41.088585
C	-0.444930	-0.888570	-42.785707
O	0.108624	2.444662	-39.820177
C	-0.563523	1.628019	-34.475653
C	0.417147	0.727609	-34.030295
H	0.494838	0.502858	-32.972906
C	1.285756	0.124421	-34.930604
Cl	2.494133	-1.005630	-34.337077
C	1.210022	0.399228	-36.295369
H	1.887952	-0.078448	-36.994108
C	0.241090	1.285440	-36.755579
H	0.160914	1.481274	-37.821078
C	-0.639616	1.907322	-35.859976
N	-1.616538	2.809812	-36.357799
C	-1.492969	3.803174	-37.332454
C	-0.261510	4.160754	-38.003050
H	-0.335377	5.131431	-38.489709
H	0.651773	4.033803	-37.421858
H	-0.068251	3.351147	-38.991677
N	-2.672880	4.377789	-37.557151
C	-3.561219	3.777020	-36.716702
H	-4.602117	4.069802	-36.681406
C	-2.944931	2.801917	-35.961436
C	-3.379928	1.912675	-34.849344
H	-3.122693	0.864592	-35.071895
H	-4.465051	1.972327	-34.738815
N	-2.761466	2.341301	-33.587744
C	-1.489718	2.221033	-33.456373
C	-0.919784	2.640645	-32.138679
C	-1.659136	2.437263	-30.961109
H	-2.625757	1.953971	-31.051363
C	-1.174363	2.843048	-29.721102
H	-1.761735	2.664404	-28.825970
C	0.067803	3.477899	-29.631655
H	0.453064	3.797291	-28.668028
C	0.817351	3.708472	-30.784037
H	1.777155	4.212658	-30.755153
C	0.313845	3.291037	-32.010632
F	1.047734	3.552156	-33.114550
H	4.992443	0.216847	-38.351064
H	3.639080	-1.763458	-39.613235
H	4.676092	5.456333	-38.259034
H	3.127116	7.308594	-39.490877
H	-2.109660	-0.124097	-44.009160
H	-0.473070	-1.965628	-42.884475
H	-2.396926	5.117215	-43.940810
H	-0.970258	7.112293	-42.787412

$^{2}PC_{H1'1}$

H	1.437920	0.434160	-5.588530
S	2.300181	0.448833	-4.550029
N	1.857933	-1.128754	-1.975402
C	1.772205	-2.475371	-2.225999
C	2.877439	-0.975523	-1.069430
C	2.757148	-3.191115	-1.448776
C	3.443075	-2.259506	-0.729530
C	3.319064	0.238105	-0.558620
H	4.138724	0.216750	0.152182
C	2.807003	1.478353	-0.913512
N	1.776594	1.686130	-1.799842
C	1.625077	3.048511	-1.887752
C	2.570660	3.713554	-1.022931
C	3.301890	2.739366	-0.416425
C	0.689274	3.708594	-2.673734
H	0.684847	4.793202	-2.635895
C	-0.244078	3.098066	-3.501185
C	-1.216373	3.812904	-4.292648
C	-1.425743	1.590098	-4.572851
N	-0.391227	1.742498	-3.678265
C	-1.950928	2.877938	-4.954698
C	-1.891681	0.374493	-5.056653
H	-2.718302	0.396403	-5.759284

C	-1.361314	-0.866098	-4.724322
C	-1.821171	-2.124361	-5.258345
N	-0.316606	-1.071121	-3.852598
C	-0.098988	-2.428423	-3.837377
C	0.868008	-3.083655	-3.087114
H	0.920537	-4.163760	-3.180248
Fe	0.798285	0.312333	-2.900269
C	-1.036193	-3.092610	-4.710827
O	-0.749365	0.070756	-1.293574
C	-1.032211	-0.937109	3.695378
C	0.008469	-1.711427	4.231691
H	0.120989	-1.780963	5.307389
C	0.894489	-2.384779	3.400073
Cl	2.176103	-3.352936	4.109879
C	0.780412	-2.302360	2.013129
H	1.476095	-2.823796	1.365653
C	-0.249967	-1.545364	1.464861
H	-0.359611	-1.489220	0.387469
C	-1.153144	-0.861382	2.288899
N	-2.194347	-0.099121	1.694304
C	-2.162095	0.722195	0.576436
C	-0.954454	1.043527	-0.237478
H	-1.088363	2.036743	-0.671914
H	-0.036645	1.029986	0.350179
H	-1.556039	0.042121	-1.830838
N	-3.361614	1.198089	0.303487
C	-4.203869	0.687682	1.259919
H	-5.257402	0.930181	1.279186
C	-3.514490	-0.115173	2.132836
C	-3.868916	-0.849497	3.380395
H	-3.564263	-1.905593	3.304473
H	-4.952571	-0.827050	3.516605
N	-3.242228	-0.211751	4.544168
C	-1.963380	-0.249534	4.649613
C	-1.380667	0.384625	5.872104
C	-2.072102	0.313960	7.093391
H	-3.011363	-0.227900	7.109600
C	-1.574621	0.919235	8.243800
H	-2.123552	0.840590	9.176852
C	-0.369139	1.625288	8.194923
H	0.025477	2.100054	9.088130
C	0.331331	1.727245	6.994042
H	1.260651	2.280694	6.914856
C	-0.183658	1.111346	5.859454
F	0.499590	1.249015	4.701736
H	4.261823	-2.409222	-0.037911
H	2.896708	-4.264131	-1.468818
H	4.107346	2.846221	0.298166
H	2.650318	4.786585	-0.908023
H	-2.636782	-2.234230	-5.960893
H	-1.075656	-4.162027	-4.871536
H	-2.773513	3.028539	-5.641312
H	-1.312225	4.890280	-4.323052

$^{2}RC_{H41}$

H	0.343436	-0.552938	-5.573386
S	0.072560	0.645209	-5.015070
N	1.846483	-0.507330	-2.977676
C	2.411570	-1.710264	-3.337678
C	2.900542	0.358165	-2.784248
C	3.847817	-1.589147	-3.400085
C	4.150184	-0.308522	-3.057765
C	2.786127	1.673813	-2.368457
H	3.704207	2.241048	-2.252507
C	1.591195	2.332421	-2.100701
N	0.342360	1.775903	-2.191091
C	-0.539005	2.766389	-1.832861
C	0.173007	3.980695	-1.516271
C	1.497546	3.711194	-1.683489
C	-1.918308	2.631778	-1.784326
H	-2.491930	3.494061	-1.462274
C	-2.624473	1.474889	-2.097607
C	-4.063512	1.371924	-2.069636
C	-3.115040	-0.568273	-2.695681
N	-2.066498	0.286970	-2.481936
C	-4.369284	0.100124	-2.444449
C	-3.000586	-1.893244	-3.097065
H	-3.920241	-2.455582	-3.224247
C	-1.807568	-2.560965	-3.339429
C	-1.713507	-3.945308	-3.734391
N	-0.557598	-2.006732	-3.238801
C	0.322640	-3.007762	-3.552142
C	1.707239	-2.875462	-3.594237
H	2.282718	-3.756526	-3.859924
Fe	-0.107827	-0.156116	-2.576352
C	-0.387802	-4.224504	-3.863373
O	0.009339	-0.619543	-1.021235
C	0.393818	-0.124608	3.059088
C	1.654581	-0.713592	3.243936
H	1.736643	-1.625428	3.823832
C	2.793961	-0.143691	2.691959

Cl	4.354799	-0.912487	2.938994
C	2.714533	1.026448	1.939005
H	3.605574	1.455305	1.495167
C	1.472016	1.617989	1.743999
H	1.392529	2.500252	1.121030
C	0.312761	1.066266	2.302788
N	-0.933570	1.708356	2.092387
C	-1.248145	3.060529	2.126483
C	-0.297373	4.149812	2.509424
H	-0.888333	5.011014	2.826248
H	0.360585	3.849014	3.330424
H	0.337094	4.468292	1.673642
N	-2.516543	3.254395	1.829847
C	-3.053311	2.005519	1.598697
H	-4.095846	1.872950	1.344290
C	-2.104412	1.031858	1.749990
C	-2.106103	-0.457173	1.702495
H	-1.326652	-0.816404	1.013174
H	-3.070661	-0.802681	1.323143
N	-1.919781	-1.006790	3.053308
C	-0.798535	-0.819949	3.647528
C	-0.662480	-1.427920	5.008648
C	-1.280109	-2.658368	5.288855
H	-1.824350	-3.141029	4.484512
C	-1.203274	-3.238247	6.552061
H	-1.681714	-4.194759	6.738015
C	-0.509183	-2.588767	7.576910
H	-0.444305	-3.034552	8.564940
C	0.100828	-1.359109	7.334150
H	0.634028	-0.818116	8.108360
C	0.012484	-0.802083	6.063543
F	0.590077	0.403720	5.863090
H	5.123628	0.158330	-2.988024
H	4.521245	-2.392067	-3.668913
H	2.342785	4.374516	-1.554994
H	-0.297284	4.908204	-1.218379
H	-2.559892	-4.601499	-3.887660
H	0.081740	-5.157428	-4.145585
H	-5.343774	-0.358237	-2.548097
H	-4.732433	2.177930	-1.799794

$^{2}TS_{H41}$

H	1.523342	1.510915	-43.436911
S	1.256220	2.784889	-43.085351
N	3.070363	1.947262	-40.784086
C	3.607446	0.691241	-40.882036
C	4.129608	2.809394	-40.679904
C	5.050256	0.761329	-40.837148
C	5.374171	2.075690	-40.714795
C	4.034924	4.187230	-40.556744
H	4.963016	4.746618	-40.496898
C	2.851170	4.911230	-40.499129
N	1.586555	4.384735	-40.554255
C	0.724927	5.442912	-40.427076
C	1.464405	6.673887	-40.289285
C	2.784840	6.343920	-40.339494
C	-0.660646	5.355295	-40.441552
H	-1.222198	6.277195	-40.336151
C	-1.381718	4.180139	-40.612673
C	-2.820151	4.115223	-40.700122
C	-1.901297	2.072715	-40.923119
N	-0.840904	2.927334	-40.737396
C	-3.142374	2.807117	-40.894421
C	-1.809868	0.703046	-41.117587
H	-2.738015	0.153806	-41.238276
C	-0.626950	-0.023577	-41.168919
C	-0.556853	-1.455494	-41.332704
N	0.631722	0.502085	-41.051302
C	1.497102	-0.560023	-41.117895
C	2.880994	-0.480869	-41.034067
H	3.438624	-1.409172	-41.104841
Fe	1.104558	2.432113	-40.689791
C	0.762134	-1.788821	-41.297962
O	1.189588	2.167949	-39.028510
C	1.935685	2.868981	-35.805587
C	3.233762	2.346571	-35.699734
H	3.372228	1.333628	-35.341354
C	4.338472	3.106525	-36.060226
Cl	5.947478	2.419863	-35.916445
C	4.184466	4.404045	-36.544312
H	5.048895	4.985195	-36.843996
C	2.903127	4.932358	-36.656550
H	2.770278	5.920411	-37.078653
C	1.779822	4.189184	-36.278589
N	0.490658	4.778707	-36.393086
C	0.112693	6.090314	-36.154511
C	0.961698	7.121423	-35.483572
H	0.295675	7.842401	-35.006083
H	1.618143	6.682230	-34.727533
H	1.589533	7.673479	-36.193061
N	-1.132709	6.305172	-36.543025

C	-1.581600	5.121235	-37.059689
H	-2.588961	5.003621	-37.434364
C	-0.610920	4.142797	-36.970435
C	-0.607989	2.711298	-37.264721
H	0.232864	2.471652	-38.165066
H	-1.552041	2.401819	-37.717786
N	-0.295385	1.864756	-36.153006
C	0.791235	1.966717	-35.473271
C	0.933090	1.017614	-34.329792
C	0.411409	-0.284320	-34.428270
H	-0.060278	-0.567767	-35.362688
C	0.497389	-1.180509	-33.367147
H	0.097293	-2.183879	-33.473590
C	1.101075	-0.788418	-32.168572
H	1.171503	-1.482471	-31.336539
C	1.613669	0.501254	-32.035145
H	2.074693	0.845279	-31.115712
C	1.520011	1.377300	-33.109694
F	1.999967	2.630537	-32.947218
H	6.355741	2.526199	-40.651503
H	5.709811	-0.094220	-40.897262
H	3.646169	6.996693	-40.283202
H	1.014630	7.651992	-40.181512
H	-1.414646	-2.103930	-41.452418
H	1.213700	-2.768273	-41.384321
H	-4.121707	2.364221	-41.018473
H	-3.479223	4.970699	-40.633189

$^{2}IM_{H41}$

H	-1.590666	-2.557693	-4.413592
S	-1.344929	-1.235001	-4.505334
N	1.602954	-1.274539	-3.538879
C	2.260402	-2.460263	-3.729381
C	2.353848	-0.309897	-4.156719
C	3.476310	-2.235665	-4.475438
C	3.534788	-0.902757	-4.739050
C	2.027633	1.034046	-4.251827
H	2.716808	1.681810	-4.783019
C	0.878272	1.611934	-3.731080
N	-0.101720	0.957843	-3.026655
C	-1.031421	1.905923	-2.677700
C	-0.635989	3.192104	-3.195433
C	0.549057	3.010253	-3.843460
C	-2.163342	1.686936	-1.904485
H	-2.808263	2.536551	-1.707738
C	-2.531327	0.463634	-1.365560
C	-3.730745	0.242369	-0.595743
C	-2.597390	-1.680709	-0.890293
N	-1.844072	-0.715944	-1.510557
C	-3.770781	-1.085095	-0.299907
C	-2.314524	-3.038018	-0.868959
H	-2.998453	-3.683877	-0.329220
C	-1.224525	-3.632955	-1.488436
C	-0.924901	-5.041938	-1.427334
N	-0.260964	-2.982521	-2.219041
C	0.654380	-3.934972	-2.591555
C	1.826507	-3.704258	-3.297566
H	2.452890	-4.561002	-3.522059
Fe	-0.125132	-0.998758	-2.540024
C	0.238680	-5.228636	-2.110445
O	0.840613	-0.754583	-1.066445
C	0.450240	1.258971	3.852821
C	0.993502	0.626084	4.990828
H	1.021692	-0.455535	5.027530
C	1.517899	1.355659	6.047840
Cl	2.176372	0.510939	7.438677
C	1.554608	2.747371	6.007222
H	1.994199	3.315482	6.818597
C	1.031290	3.390673	4.889180
H	1.087996	4.469960	4.831836
C	0.451340	2.676066	3.836451
N	-0.115968	3.408092	2.748632
C	-0.685805	4.669258	2.783004
C	-1.204318	5.354440	4.007670
H	-2.065533	5.956228	3.710182
H	-1.510068	4.643249	4.778157
H	-0.468922	6.038014	4.447739
N	-0.797199	5.183800	1.567668
C	-0.276790	4.257792	0.713872
H	-0.262501	4.404459	-0.357838
C	0.134201	3.125659	1.399419
C	0.471254	1.842388	0.874189
H	0.340881	-0.454996	-0.278443
H	0.766314	1.818736	-0.172709
N	0.280713	0.668350	1.437132
C	0.029383	0.397178	2.729129
C	-0.476275	-0.962786	2.997018
C	-0.094614	-2.058361	2.193017
H	0.604074	-1.884785	1.382523
C	-0.583282	-3.339364	2.430361
H	-0.259109	-4.158938	1.797029

C	-1.482248	-3.568080	3.476431
H	-1.864181	-4.566397	3.667570
C	-1.893974	-2.504553	4.280680
H	-2.601966	-2.636205	5.091869
C	-1.393560	-1.235225	4.028595
F	-1.831169	-0.219259	4.807742
H	4.293418	-0.353804	-5.281050
H	4.177270	-3.011140	-4.754639
H	1.156653	3.744193	-4.356108
H	-1.204338	4.104705	-3.071537
H	-1.534879	-5.777376	-0.919423
H	0.783845	-6.148477	-2.276065
H	-4.521773	-1.630857	0.255586
H	-4.442194	1.014416	-0.334332

$^{2}PC_{H41}$

H	-0.913705	-2.729565	-4.461314
S	0.115339	-1.888402	-4.226075
N	1.231450	-1.219274	-1.455711
C	1.743692	-2.332808	-0.836901
C	2.259528	-0.308191	-1.504719
C	3.123416	-2.111180	-0.472637
C	3.442658	-0.853813	-0.885032
C	2.181701	0.954482	-2.078502
H	3.071746	1.574829	-2.052646
C	1.053668	1.471369	-2.702951
N	-0.150460	0.820740	-2.827010
C	-0.967031	1.670064	-3.531413
C	-0.266146	2.893815	-3.841478
C	0.987351	2.771020	-3.326611
C	-2.280335	1.410787	-3.898791
H	-2.804369	2.186222	-4.448292
C	-2.981414	0.245124	-3.621122
C	-4.358062	0.019947	-3.996493
C	-3.520084	-1.748865	-2.886903
N	-2.489692	-0.843177	-2.945785
C	-4.692828	-1.216916	-3.539742
C	-3.451575	-3.003418	-2.294924
H	-4.339951	-3.625696	-2.329925
C	-2.323293	-3.526399	-1.677274
C	-2.237551	-4.847170	-1.106435
N	-1.121340	-2.868707	-1.538984
C	-0.275923	-3.755431	-0.913190
C	1.050773	-3.510167	-0.582855
H	1.595695	-4.310898	-0.092550
Fe	-0.606794	-1.060848	-2.277505
C	-0.967764	-4.990046	-0.635087
O	-1.395161	-0.279207	-0.344085
C	1.111343	1.595764	2.545586
C	2.471838	1.760051	2.848482
H	3.068654	0.892564	3.104742
C	3.058784	3.018528	2.819424
Cl	4.764965	3.187664	3.190258
C	2.308259	4.147852	2.492998
H	2.774809	5.125904	2.461682
C	0.960704	3.997628	2.186155
H	0.379214	4.864333	1.896888
C	0.346366	2.738609	2.214499
N	-1.031258	2.626993	1.903540
C	-2.094851	3.443953	2.278470
C	-1.992073	4.598495	3.223626
H	-2.987789	4.781748	3.630672
H	-1.302258	4.395139	4.048318
H	-1.662361	5.521176	2.731089
N	-3.216512	3.030827	1.732136
C	-2.901841	1.923008	0.973761
H	-3.646769	1.384518	0.407009
C	-1.567030	1.648700	1.064292
C	-0.678316	0.554933	0.562693
H	-1.621611	-1.090590	0.139802
H	0.181550	0.957321	0.011878
N	-0.247803	-0.267437	1.678552
C	0.545577	0.210025	2.567232
C	0.974817	-0.722036	3.650863
C	1.205229	-2.077563	3.359794
H	1.090553	-2.403171	2.331370
C	1.577028	-2.974947	4.357001
H	1.759494	-4.015271	4.106978
C	1.717190	-2.535424	5.676505
H	2.006408	-3.230955	6.458490
C	1.482220	-1.199023	5.996754
H	1.566545	-0.826607	7.011785
C	1.116039	-0.319880	4.985006
F	0.867321	0.964692	5.321392
H	4.386686	-0.332773	-0.793612
H	3.752206	-2.835751	0.028239
H	1.803987	3.480165	-3.361135
H	-0.693414	3.726395	-4.384813
H	-3.050225	-5.561218	-1.081028
H	-0.525084	-5.846427	-0.143367
H	-5.638833	-1.735242	-3.625322

H	-4.972825	0.729458	-4.534627

$^{2}RC_{H42}$

H	0.432993	1.165877	-4.732946
S	1.781868	1.165993	-4.699199
N	3.815721	-0.004208	-2.771688
C	4.060773	-1.297642	-3.153775
C	5.038380	0.620035	-2.753828
C	5.470735	-1.493213	-3.390280
C	6.077640	-0.300626	-3.146052
C	5.253269	1.944030	-2.401086
H	6.275366	2.307927	-2.433530
C	4.269755	2.843850	-2.004217
N	2.934467	2.562319	-1.907383
C	2.335285	3.723438	-1.498180
C	3.318097	4.766178	-1.330432
C	4.523038	4.218643	-1.646875
C	0.972299	3.890030	-1.282577
H	0.636685	4.866741	-0.949503
C	0.006066	2.907400	-1.446512
C	-1.402306	3.100759	-1.201475
C	-0.969639	0.996093	-1.863920
N	0.248526	1.620831	-1.856080
C	-2.009046	1.911048	-1.459370
C	-1.190224	-0.329278	-2.224085
H	-2.208984	-0.697060	-2.166432
C	-0.214501	-1.221568	-2.637462
C	-0.462278	-2.592909	-3.007951
N	1.128966	-0.937531	-2.733708
C	1.733364	-2.111038	-3.125233
C	3.095847	-2.285304	-3.306841
H	3.435888	-3.270540	-3.609504
Fe	2.049954	0.774988	-2.172571
C	0.744324	-3.144508	-3.308152
O	2.232618	0.299479	-0.626115
C	-2.204333	-1.400521	3.429455
C	-2.885902	-2.387375	4.158979
H	-3.391545	-3.186796	3.629734
C	-2.921544	-2.351858	5.547028
Cl	-3.776032	-3.609931	6.425159
C	-2.282318	-1.332073	6.250520
H	-2.303392	-1.315589	7.334288
C	-1.598365	-0.351648	5.540755
H	-1.067836	0.423799	6.079608
C	-1.555700	-0.363179	4.139664
N	-0.853328	0.655891	3.454965
C	-0.800162	2.022485	3.709744
C	-1.615393	2.731697	4.744463
H	-1.673034	3.782943	4.456925
H	-2.629516	2.326912	4.818729
H	-1.165831	2.688652	5.744111
N	0.022128	2.621979	2.876589
C	0.531918	1.638568	2.053704
H	1.240279	1.849167	1.265682
C	0.009284	0.418293	2.379292
C	0.115409	-0.945622	1.791478
H	0.409972	-1.682329	2.556434
H	0.879567	-0.931771	1.012400
N	-1.164263	-1.341729	1.181740
C	-2.187312	-1.517441	1.933615
C	-3.444329	-1.954292	1.246432
C	-3.374524	-2.840339	0.157828
H	-2.394146	-3.203305	-0.131828
C	-4.519502	-3.239176	-0.527699
H	-4.439968	-3.936933	-1.355744
C	-5.770565	-2.748327	-0.142106
H	-6.668009	-3.055871	-0.670305
C	-5.869995	-1.858006	0.925841
H	-6.821321	-1.446298	1.245007
C	-4.712991	-1.477827	1.596932
F	-4.834093	-0.594543	2.612175
H	7.127527	-0.049690	-3.218384
H	5.917933	-2.425976	-3.707149
H	5.498837	4.685781	-1.645918
H	3.096468	5.776691	-1.014272
H	-1.440044	-3.055371	-3.027672
H	0.964099	-4.155177	-3.625615
H	-3.057498	1.655936	-1.384101
H	-1.846730	4.028574	-0.867556

$^{2}TS_{H42}$

H	0.159020	2.856826	-43.408571
S	1.450638	3.146813	-43.147299
N	3.237996	1.920998	-41.085705
C	3.513199	0.618606	-41.399792
C	4.451095	2.546719	-40.972295
C	4.941980	0.413216	-41.489972
C	5.524936	1.613543	-41.230066
C	4.625004	3.884880	-40.646515

H	5.644006	4.256298	-40.597521
C	3.605888	4.789086	-40.359126
N	2.269074	4.501208	-40.368910
C	1.631849	5.660217	-40.021612
C	2.591470	6.713083	-39.785231
C	3.821415	6.170585	-39.998283
C	0.252824	5.812704	-39.913528
H	-0.115127	6.792811	-39.626876
C	-0.695658	4.825715	-40.149534
C	-2.121914	5.037969	-40.080097
C	-1.638552	2.929625	-40.692677
N	-0.420934	3.530782	-40.512353
C	-2.708685	3.858887	-40.420620
C	-1.824466	1.613380	-41.101289
H	-2.846324	1.262001	-41.198151
C	-0.809273	0.709447	-41.383522
C	-1.012560	-0.662408	-41.780316
N	0.532591	0.985989	-41.294342
C	1.181931	-0.186658	-41.602659
C	2.558732	-0.365549	-41.627203
H	2.921364	-1.358476	-41.874939
Fe	1.397534	2.712719	-40.725088
C	0.222784	-1.220226	-41.908214
O	1.631776	2.218998	-39.075819
C	-2.269173	0.929973	-35.479305
C	-2.924770	-0.099966	-34.785803
H	-3.459051	-0.860678	-35.342367
C	-2.891674	-0.162052	-33.398569
Cl	-3.717313	-1.469717	-32.568956
C	-2.200890	0.797703	-32.661802
H	-2.160856	0.737205	-31.580146
C	-1.544172	1.820302	-33.337368
H	-0.974426	2.546848	-32.772189
C	-1.578367	1.913209	-34.735630
N	-0.908679	2.983722	-35.375012
C	-0.767426	4.298217	-34.940605
C	-1.537065	4.921310	-33.817750
H	-1.591142	5.994157	-34.011696
H	-2.552043	4.521239	-33.740906
H	-1.050200	4.790569	-32.843788
N	0.082748	4.956255	-35.697879
C	0.529334	4.065738	-36.647232
H	1.236628	4.342200	-37.413981
C	-0.066001	2.841824	-36.490798
C	-0.033435	1.606839	-37.301567
H	0.614552	0.794355	-36.947166
H	0.712301	1.941979	-38.351109
N	-1.295806	1.129561	-37.738438
C	-2.293976	0.880161	-36.975528
C	-3.539008	0.381216	-37.638909
C	-3.459987	-0.544830	-38.691437
H	-2.477007	-0.892338	-38.990601
C	-4.607157	-1.004671	-39.335011
H	-4.523566	-1.734578	-40.134348
C	-5.864078	-0.532953	-38.945010
H	-6.761641	-0.887024	-39.442931
C	-5.971030	0.396776	-37.911299
H	-6.928240	0.792475	-37.589730
C	-4.813320	0.833656	-37.278219
F	-4.933916	1.745552	-36.289559
H	6.577668	1.862379	-41.208549
H	5.416035	-0.529464	-41.729378
H	4.790889	6.644527	-39.920518
H	2.339906	7.724321	-39.494680
H	-1.979552	-1.121652	-41.937607
H	0.479092	-2.233012	-42.189922
H	-3.762290	3.623022	-40.489095
H	-2.592581	5.974328	-39.811507

$^{2}IM_{H42}$

H	3.440843	1.177773	-4.483997
S	3.570098	0.974012	-3.156429
N	1.562325	-1.027305	-1.848697
C	1.438704	-2.203268	-2.540414
C	1.899299	-1.370232	-0.573261
C	1.713037	-3.325682	-1.670189
C	1.995086	-2.807764	-0.444093
C	2.124217	-0.464993	0.465101
H	2.395287	-0.876234	1.432712
C	2.086437	0.922941	0.347708
N	1.767159	1.600822	-0.793655
C	1.847286	2.932376	-0.481568
C	2.230620	3.099029	0.903634
C	2.377448	1.849049	1.420870
C	1.590309	3.980280	-1.356758
H	1.689770	4.987074	-0.962550
C	1.216891	3.862137	-2.692469
C	0.950765	4.985039	-3.563952
C	0.667389	3.030869	-4.641679
N	1.041844	2.685555	-3.368893
C	0.607699	4.468609	-4.774836

C	0.391826	2.127660	-5.662042
H	0.106818	2.536953	-6.626467
C	0.467779	0.741860	-5.554008
C	0.218046	-0.183081	-6.634420
N	0.810950	0.062262	-4.413021
C	0.793676	-1.265009	-4.745034
C	1.088093	-2.317053	-3.879245
H	1.033919	-3.320554	-4.290659
Fe	1.275815	0.829245	-2.599377
C	0.423974	-1.431736	-6.131842
O	-0.507780	0.785360	-2.071996
C	-0.970497	-0.938406	3.633651
C	-0.361631	-1.626132	4.700066
H	-0.408058	-2.708098	4.726103
C	0.311734	-0.942685	5.702464
Cl	1.057158	-1.837163	7.012009
C	0.418860	0.447197	5.666087
H	0.968581	0.977182	6.435365
C	-0.171553	1.140841	4.614054
H	-0.055358	2.215516	4.561865
C	-0.885466	0.474674	3.611307
N	-1.495723	1.234468	2.570688
C	-1.937245	2.540616	2.625430
C	-2.334489	3.266729	3.870109
H	-3.184430	3.907073	3.624251
H	-2.616682	2.583835	4.674139
H	-1.535934	3.921431	4.238114
N	-2.044347	3.074855	1.412983
C	-1.633780	2.124737	0.534237
H	-1.604107	2.253819	-0.539593
C	-1.307232	0.948890	1.201878
C	-1.078529	-0.338275	0.666716
H	-0.804295	-0.361670	-0.390514
H	-1.041149	0.797011	-2.879434
N	-1.401124	-1.474434	1.267101
C	-1.582681	-1.734490	2.547816
C	-2.298082	-2.988609	2.840621
C	-2.267847	-4.050933	1.909632
H	-1.668985	-3.916142	1.016084
C	-2.966220	-5.230529	2.133152
H	-2.911683	-6.034852	1.406568
C	-3.732841	-5.383151	3.293793
H	-4.280583	-6.302996	3.473658
C	-3.799609	-4.347365	4.225402
H	-4.398999	-4.421667	5.126131
C	-3.091693	-3.177618	3.987493
F	-3.208451	-2.179261	4.889836
H	2.254480	-3.331372	0.466754
H	1.689661	-4.363744	-1.975166
H	2.665485	1.561955	2.423879
H	2.370274	4.053719	1.393774
H	-0.069227	0.101322	-7.638201
H	0.339335	-2.384262	-6.638393
H	0.339685	4.994637	-5.681703
H	1.021934	6.024013	-3.269995

$^{2}PC_{H42}$

H	2.304275	0.305109	-4.689141
S	3.119935	0.514955	-3.634233
N	2.522799	-0.326429	-0.746947
C	2.443296	-1.695787	-0.644655
C	3.505749	0.060369	0.133702
C	3.383642	-2.177884	0.338144
C	4.040292	-1.088327	0.822840
C	3.940406	1.365047	0.323895
H	4.722472	1.533033	1.057222
C	3.467220	2.465794	-0.376766
N	2.496675	2.429306	-1.345413
C	2.357545	3.719632	-1.788772
C	3.260132	4.594109	-1.074959
C	3.947138	3.816124	-0.196765
C	1.471940	4.149096	-2.767427
H	1.478062	5.206070	-3.013833
C	0.571596	3.343004	-3.451603
C	-0.352318	3.824999	-4.449862
C	-0.583225	1.608229	-4.141465
N	0.412780	1.990216	-3.272078
C	-1.070865	2.749856	-4.875531
C	-1.047171	0.309078	-4.301021
H	-1.842460	0.143482	-5.020578
C	-0.550895	-0.797338	-3.623020
C	-1.010516	-2.149854	-3.821057
N	0.455706	-0.767931	-2.686713
C	0.649894	-2.071541	-2.299575
C	1.573761	-2.508675	-1.358606
H	1.618053	-3.575627	-1.164119
Fe	1.540519	0.820486	-2.080973
C	-0.262411	-2.941558	-3.002207
O	-0.080161	1.261400	-0.575155
C	-1.132508	-1.314737	2.658333
C	-0.938784	-2.631345	3.104347

H	-1.325045	-3.455106	2.515570
C	-0.258280	-2.888041	4.287635
Cl	-0.022488	-4.546000	4.809376
C	0.243841	-1.842523	5.061983
H	0.784036	-2.049655	5.978764
C	0.061159	-0.533953	4.629705
H	0.477526	0.281946	5.207545
C	-0.627410	-0.251317	3.442554
N	-0.803877	1.096073	3.042434
C	-1.129234	2.212960	3.808192
C	-1.480979	2.178625	5.261495
H	-2.060549	3.076486	5.482103
H	-2.076624	1.297948	5.520112
H	-0.597945	2.188377	5.911714
N	-1.141258	3.299224	3.068564
C	-0.813557	2.908252	1.787561
H	-0.743926	3.612785	0.972181
C	-0.606551	1.559071	1.740525
C	-0.329347	0.574922	0.648257
H	0.567842	-0.021914	0.860311
H	-0.899145	1.211082	-1.093127
N	-1.491259	-0.273805	0.452675
C	-1.848520	-1.107924	1.360141
C	-3.023873	-1.973409	1.047699
C	-3.207455	-2.468492	-0.254690
H	-2.460773	-2.230414	-1.004782
C	-4.311479	-3.253617	-0.576345
H	-4.425854	-3.634028	-1.586388
C	-5.266115	-3.552540	0.399774
H	-6.129375	-4.163534	0.153944
C	-5.116035	-3.064009	1.697116
H	-5.844164	-3.263634	2.475630
C	-4.004248	-2.286905	1.997538
F	-3.889589	-1.802344	3.253522
H	4.820972	-1.049231	1.571157
H	3.512792	-3.217817	0.608270
H	4.709746	4.109536	0.512534
H	3.339618	5.661065	-1.236808
H	-1.794973	-2.439542	-4.508037
H	-0.307639	-4.015693	-2.877592
H	-1.858479	2.715419	-5.616758
H	-0.429771	4.855977	-4.769376

$^{2}RC_{C7}$

H	-1.147612	0.773510	-4.914409
S	0.136053	0.366255	-4.828958
N	2.171583	0.317517	-2.576875
C	3.159618	-0.628366	-2.695394
C	2.812244	1.529344	-2.562913
C	4.454240	0.005052	-2.770556
C	4.238903	1.344876	-2.682875
C	2.188210	2.766375	-2.462922
H	2.823891	3.645891	-2.456270
C	0.819237	2.970669	-2.395067
N	-0.131470	1.974775	-2.382181
C	-1.345625	2.617265	-2.271082
C	-1.153275	4.046701	-2.245324
C	0.187423	4.264920	-2.318183
C	-2.574070	1.985252	-2.171645
H	-3.452329	2.615855	-2.077853
C	-2.776241	0.608049	-2.175667
C	-4.070709	-0.022749	-2.082662
C	-2.427758	-1.549882	-2.250258
N	-1.790439	-0.336134	-2.281344
C	-3.853315	-1.366173	-2.129822
C	-1.799815	-2.785820	-2.329798
H	-2.431481	-3.667922	-2.298884
C	-0.430533	-2.986983	-2.447527
C	0.195078	-4.283581	-2.547690
N	0.512772	-1.995453	-2.499922
C	1.716774	-2.630663	-2.630105
C	2.952460	-1.998962	-2.726408
H	3.827849	-2.632769	-2.825932
Fe	0.201759	-0.021566	-2.285417
C	1.532349	-4.061653	-2.662562
O	0.328008	-0.035158	-0.664080
C	0.029483	-0.205800	2.847769
C	0.366176	-1.542059	2.583041
H	-0.387740	-2.211606	2.186946
C	1.651979	-2.011309	2.814810
Cl	2.039333	-3.690183	2.466663
C	2.642242	-1.168085	3.315871
H	3.647185	-1.538510	3.483467
C	2.324542	0.160451	3.573677
H	3.095118	0.834273	3.927913
C	1.030708	0.651955	3.356223
N	0.747418	2.012028	3.636753
C	1.147690	2.797363	4.711300
C	1.910437	2.299383	5.897869
H	1.734272	2.994717	6.720376
H	1.588430	1.298727	6.201916

H	2.992623	2.262035	5.723052
N	0.715101	4.032576	4.575825
C	0.014389	4.068562	3.389779
H	-0.461169	4.974794	3.039999
C	0.012131	2.840834	2.786483
C	-0.644855	2.266199	1.579021
H	0.076998	1.752569	0.927032
H	-1.097971	3.074351	1.000077
N	-1.708227	1.330427	1.974182
C	-1.373770	0.236803	2.553375
C	-2.488974	-0.702104	2.892402
C	-3.572331	-0.851614	2.011291
H	-3.549831	-0.290703	1.083534
C	-4.640252	-1.692935	2.313666
H	-5.460569	-1.797922	1.610255
C	-4.653321	-2.400832	3.518713
H	-5.483252	-3.057751	3.761637
C	-3.597710	-2.262493	4.418928
H	-3.579626	-2.784098	5.369725
C	-2.541154	-1.420228	4.092617
F	-1.540758	-1.284738	4.991966
H	4.963405	2.148030	-2.702591
H	5.392735	-0.522832	-2.875194
H	0.717319	5.208076	-2.320595
H	-1.951759	4.773316	-2.174910
H	-0.334651	-5.226702	-2.533084
H	2.331434	-4.784137	-2.761016
H	-4.577158	-2.169349	-2.092479
H	-5.009960	0.507573	-1.998664

$^{2}TS_{C7}$

H	-1.234785	0.165165	-4.396476
S	0.081066	-0.124380	-4.340127
N	2.276383	-0.234733	-2.336937
C	2.990957	-1.392262	-2.495626
C	3.172059	0.794149	-2.494104
C	4.375443	-1.089870	-2.762520
C	4.489346	0.266142	-2.752314
C	2.870934	2.147071	-2.441298
H	3.690732	2.845383	-2.574469
C	1.599632	2.678216	-2.276797
N	0.450859	1.951581	-2.118191
C	-0.572881	2.860387	-2.052590
C	-0.052639	4.203466	-2.163979
C	1.295837	4.090473	-2.297968
C	-1.916204	2.545497	-1.912779
H	-2.624080	3.367704	-1.892585
C	-2.428596	1.260196	-1.792542
C	-3.829587	0.957284	-1.639465
C	-2.590825	-0.921713	-1.611807
N	-1.691702	0.100731	-1.784554
C	-3.930215	-0.395993	-1.526481
C	-2.269770	-2.269717	-1.532322
H	-3.084690	-2.970316	-1.385266
C	-0.993209	-2.793341	-1.669648
C	-0.697754	-4.203958	-1.718485
N	0.154947	-2.061888	-1.861307
C	1.154375	-2.977878	-2.082464
C	2.473776	-2.675733	-2.383158
H	3.154987	-3.505956	-2.537341
Fe	0.316483	-0.062169	-1.904676
C	0.632036	-4.317823	-1.982884
O	0.567482	0.212082	-0.222845
C	-0.061466	0.280104	2.209268
C	0.665515	-0.660686	1.383558
H	0.142771	-1.554986	1.067034
C	2.076914	-0.767436	1.598361
Cl	2.926467	-2.154729	0.980164
C	2.773666	0.220609	2.271410
H	3.854439	0.176035	2.345003
C	2.067020	1.261790	2.871714
H	2.606698	2.064055	3.358652
C	0.656270	1.271374	2.886126
N	-0.012677	2.289573	3.611539
C	0.297935	2.859294	4.841147
C	1.400658	2.405904	5.744872
H	1.152409	2.730061	6.756991
H	1.516743	1.317862	5.737607
H	2.371040	2.846992	5.486602
N	-0.548136	3.821957	5.138655
C	-1.439029	3.893868	4.090163
H	-2.255543	4.603002	4.087974
C	-1.139967	2.963663	3.133697
C	-1.791446	2.493643	1.879105
H	-1.075293	2.473333	1.044768
H	-2.602345	3.176133	1.614133
N	-2.362163	1.158508	2.101845
C	-1.549295	0.174477	2.253055
C	-2.170777	-1.168837	2.484214
C	-3.464248	-1.426768	1.995746
H	-3.946359	-0.639898	1.427586

C	-4.104921	-2.638551	2.233590
H	-5.102959	-2.808369	1.841042
C	-3.465846	-3.633881	2.979528
H	-3.959142	-4.582384	3.169763
C	-2.189795	-3.405287	3.488922
H	-1.666280	-4.144348	4.085576
C	-1.571210	-2.184980	3.239062
F	-0.342891	-1.992449	3.776001
H	5.372373	0.870515	-2.912384
H	5.146089	-1.831202	-2.926838
H	2.031013	4.875864	-2.413113
H	-0.656565	5.100948	-2.143046
H	-1.430302	-4.989317	-1.588000
H	1.220298	-5.216833	-2.110448
H	-4.819929	-0.998232	-1.399420
H	-4.619202	1.696567	-1.618591

$^{2}RC_{C9}$

H	-0.424812	0.370949	-5.317619
S	0.918393	0.489765	-5.268232
N	3.031499	-0.540982	-3.367499
C	3.399215	-1.800215	-3.766015
C	4.187311	0.200134	-3.350109
C	4.820422	-1.854764	-4.009649
C	5.308683	-0.610247	-3.759045
C	4.275471	1.532030	-2.974113
H	5.256503	1.995105	-3.008612
C	3.215543	2.317892	-2.533695
N	1.915619	1.903709	-2.430831
C	1.217637	2.977364	-1.944843
C	2.099801	4.098460	-1.732215
C	3.342901	3.690281	-2.106841
C	-0.149769	3.002792	-1.699514
H	-0.560808	3.911527	-1.275858
C	-1.025414	1.950120	-1.931093
C	-2.446489	2.006858	-1.693013
C	-1.833817	-0.002583	-2.497176
N	-0.672187	0.719432	-2.424274
C	-2.950426	0.793688	-2.051014
C	-1.933710	-1.321590	-2.931638
H	-2.922864	-1.768390	-2.938602
C	-0.875119	-2.115959	-3.339838
C	-0.992758	-3.493232	-3.751455
N	0.443146	-1.716681	-3.382709
C	1.156805	-2.823558	-3.782508
C	2.532620	-2.871794	-3.940154
H	2.965278	-3.816080	-4.254609
Fe	1.200842	0.054044	-2.756908
C	0.265981	-3.932872	-4.019570
O	1.413302	-0.442870	-1.218867
C	-1.782629	-0.451215	3.480372
C	-1.774365	-1.842491	3.669824
H	-2.311254	-2.269941	4.508724
C	-1.086567	-2.673291	2.794721
Cl	-1.110575	-4.411350	3.056261
C	-0.385213	-2.147474	1.710838
H	0.144414	-2.796676	1.022969
C	-0.387678	-0.771762	1.505455
H	0.130266	-0.375454	0.638851
C	-1.070540	0.082123	2.381140
N	-1.050456	1.482494	2.146954
C	0.004875	2.298392	1.764912
C	1.422818	1.844233	1.628919
H	2.057722	2.731624	1.642330
H	1.714659	1.186879	2.454450
H	1.607119	1.304771	0.693439
N	-0.405084	3.540552	1.593011
C	-1.755540	3.545710	1.869636
H	-2.341103	4.454296	1.825693
C	-2.184451	2.292985	2.213787
C	-3.469382	1.718379	2.701161
H	-3.756857	0.844851	2.093855
H	-4.261075	2.465093	2.602607
N	-3.359767	1.347524	4.117829
C	-2.575582	0.383837	4.441265
C	-2.527881	0.030583	5.894231
C	-3.689332	0.136903	6.677924
H	-4.600517	0.457488	6.184878
C	-3.675406	-0.153375	8.039169
H	-4.589579	-0.072564	8.618870
C	-2.484984	-0.549567	8.655578
H	-2.465737	-0.778134	9.716928
C	-1.312635	-0.648494	7.908107
H	-0.367406	-0.936679	8.355074
C	-1.352342	-0.356509	6.549514
F	-0.196264	-0.433059	5.855038
H	6.328346	-0.256887	-3.835218
H	5.354852	-2.736178	-4.338028
H	4.269287	4.248948	-2.096683
H	1.788522	5.058795	-1.344560
H	-1.924995	-4.038717	-3.813566

H	0.581071	-4.913426	-4.350679
H	-3.976540	0.452145	-2.019325
H	-2.967645	2.867864	-1.298057

$^{2}TS_{C9}$

H	-0.142942	-0.187389	-5.233022
S	1.116555	-0.168874	-4.751200
N	2.380262	-1.112990	-2.188609
C	2.649716	-2.454615	-2.302003
C	3.591728	-0.505979	-1.939285
C	4.057812	-2.699612	-2.106158
C	4.643469	-1.490469	-1.888334
C	3.768348	0.856283	-1.744308
H	4.777523	1.207246	-1.553062
C	2.753369	1.808435	-1.769998
N	1.429840	1.539576	-2.007203
C	0.789072	2.753932	-1.973878
C	1.727829	3.808769	-1.677033
C	2.951184	3.221191	-1.555031
C	-0.562598	2.961048	-2.222321
H	-0.926776	3.980822	-2.146493
C	-1.484447	1.982552	-2.573522
C	-2.883546	2.233522	-2.832118
C	-2.384351	0.051837	-3.064060
N	-1.201711	0.652049	-2.727367
C	-3.443165	1.032006	-3.138838
C	-2.556434	-1.310148	-3.295149
H	-3.554964	-1.647581	-3.554834
C	-1.562462	-2.273058	-3.204463
C	-1.763427	-3.688980	-3.416638
N	-0.251235	-2.021685	-2.886421
C	0.374890	-3.241204	-2.872044
C	1.717318	-3.445658	-2.588833
H	2.080820	-4.467808	-2.623832
Fe	0.578935	-0.242119	-2.360229
C	-0.562853	-4.289851	-3.206404
O	0.029110	-0.578961	-0.755947
C	-0.878066	0.118243	2.823860
C	-0.494646	-1.209560	2.531127
H	-0.956330	-2.023819	3.077266
C	0.460523	-1.482518	1.579496
Cl	0.933815	-3.133867	1.270804
C	1.032242	-0.445372	0.775304
H	1.971388	-0.647248	0.274419
C	0.742570	0.893457	1.180055
H	1.226914	1.702606	0.652709
C	-0.219617	1.175118	2.136399
N	-0.530646	2.528045	2.439409
C	0.311267	3.619896	2.596279
C	1.805242	3.554310	2.586470
H	2.180279	4.428098	3.122341
H	2.177621	2.648966	3.075577
H	2.219390	3.581002	1.571496
N	-0.381848	4.720755	2.802399
C	-1.708531	4.351988	2.781730
H	-2.500176	5.071533	2.941076
C	-1.835076	3.007615	2.559590
C	-2.972746	2.046112	2.537059
H	-2.957259	1.445927	1.613115
H	-3.914707	2.599337	2.557435
N	-2.926702	1.174003	3.717975
C	-1.960231	0.332964	3.829231
C	-1.993577	-0.548029	5.039052
C	-3.225397	-0.992382	5.547897
H	-4.124221	-0.695115	5.018927
C	-3.295307	-1.781154	6.692777
H	-4.259842	-2.118653	7.058911
C	-2.123934	-2.137262	7.367265
H	-2.170109	-2.752217	8.261004
C	-0.887597	-1.698635	6.895812
H	0.040605	-1.941388	7.401598
C	-0.843762	-0.914848	5.748664
F	0.365501	-0.481571	5.327164
H	5.687044	-1.269096	-1.708425
H	4.522917	-3.675700	-2.143315
H	3.905981	3.687549	-1.351287
H	1.471141	4.856107	-1.590022
H	-2.707501	-4.143601	-3.686153
H	-0.314614	-5.341054	-3.268401
H	-4.471630	0.811158	-3.392049
H	-3.355895	3.205544	-2.781068

$^{2}RC_{C10}$

H	-0.446019	0.237850	-5.464975
S	0.791333	-0.087017	-5.035608
N	1.854728	-1.462945	-2.554610
C	1.864084	-2.814398	-2.779487
C	3.146608	-1.127018	-2.231105
C	3.193627	-3.345739	-2.595834
C	3.992047	-2.296200	-2.263869

C	3.579963	0.147464	-1.900653
H	4.634371	0.271511	-1.674332
C	2.772747	1.278494	-1.816456
N	1.428835	1.311059	-2.069374
C	1.035827	2.605445	-1.856868
C	2.161678	3.413607	-1.456001
C	3.245772	2.587652	-1.436758
C	-0.254548	3.093096	-2.029083
H	-0.429349	4.136912	-1.792035
C	-1.339263	2.348777	-2.470262
C	-2.662951	2.884313	-2.675831
C	-2.582325	0.691877	-3.171872
N	-1.309410	1.013790	-2.784053
C	-3.435782	1.854542	-3.116034
C	-3.006974	-0.574359	-3.564937
H	-4.049774	-0.683369	-3.845654
C	-2.213715	-1.708719	-3.614897
C	-2.680061	-3.021469	-3.989695
N	-0.876552	-1.753731	-3.287107
C	-0.503018	-3.072451	-3.416668
C	0.763226	-3.572125	-3.160438
H	0.915562	-4.637862	-3.297216
Fe	0.241331	-0.247233	-2.526548
C	-1.622525	-3.866508	-3.861044
O	-0.144360	-0.594810	-0.984966
C	-0.109911	-0.038191	3.158843
C	0.785081	-1.042693	3.559644
H	0.430826	-1.852863	4.186234
C	2.114866	-1.010538	3.161722
Cl	3.205368	-2.288625	3.677263
C	2.594101	0.018759	2.353304
H	3.628810	0.027223	2.030576
C	1.718011	1.017684	1.945325
H	2.069244	1.795659	1.278924
C	0.376342	1.010360	2.345967
N	-0.482364	2.054676	1.919900
C	-0.242692	3.420030	1.843509
C	1.001547	4.097096	2.323765
H	0.761416	5.146889	2.501489
H	1.374360	3.655989	3.253274
H	1.813844	4.058621	1.588163
N	-1.285051	4.051786	1.344296
C	-2.235341	3.085544	1.089262
H	-3.204573	3.335482	0.680421
C	-1.771756	1.844863	1.430370
C	-2.351100	0.472494	1.452308
H	-1.695609	-0.224403	0.908366
H	-3.318947	0.483101	0.945598
N	-2.563940	0.035249	2.839738
C	-1.541929	-0.160670	3.589689
C	-1.824045	-0.630180	4.982728
C	-2.906666	-1.492789	5.223317
H	-3.494990	-1.809564	4.369118
C	-3.219617	-1.923799	6.509430
H	-4.055793	-2.598283	6.665239
C	-2.455860	-1.488725	7.596305
H	-2.693244	-1.820551	8.602623
C	-1.385106	-0.620011	7.391598
H	-0.780214	-0.248515	8.211639
C	-1.090736	-0.206274	6.097496
F	-0.064124	0.657496	5.930852
H	5.053299	-2.295323	-2.053746
H	3.462834	-4.386281	-2.718704
H	4.273943	2.828045	-1.200186
H	2.113394	4.472819	-1.241420
H	-3.691101	-3.249064	-4.300363
H	-1.584357	-4.931593	-4.046916
H	-4.484816	1.862104	-3.380656
H	-2.942405	3.914592	-2.501838

$^{2}TS_{C10}$

H	-0.326849	0.028741	-4.943390
S	0.962909	0.035235	-4.551635
N	2.243444	-1.118069	-2.052191
C	2.274794	-2.492241	-2.104481
C	3.548091	-0.715244	-1.903117
C	3.630859	-2.962596	-1.964811
C	4.421669	-1.861061	-1.844856
C	3.968017	0.605348	-1.811694
H	5.032450	0.788759	-1.707214
C	3.127332	1.708642	-1.862329
N	1.757259	1.654355	-1.987004
C	1.332725	2.962062	-2.040670
C	2.457374	3.855975	-1.915784
C	3.570601	3.078334	-1.807703
C	0.020955	3.384396	-2.208483
H	-0.160913	4.453751	-2.190528
C	-1.074445	2.556882	-2.415078
C	-2.428067	3.026054	-2.601794
C	-2.319957	0.779716	-2.698012
N	-1.031070	1.190852	-2.482153

C	-3.201189	1.921844	-2.781757
C	-2.733820	-0.541857	-2.816069
H	-3.789999	-0.720156	-2.991504
C	-1.905940	-1.651048	-2.717612
C	-2.352315	-3.021904	-2.826806
N	-0.552488	-1.610375	-2.506536
C	-0.132231	-2.910993	-2.470972
C	1.179171	-3.325212	-2.280716
H	1.368161	-4.393884	-2.286779
Fe	0.605115	0.028448	-2.182048
C	-1.251675	-3.804310	-2.671198
O	0.115180	-0.149287	-0.538891
C	-0.604712	-0.335500	2.653725
C	0.123563	-1.531075	2.797531
H	-0.239367	-2.294382	3.475687
C	1.318677	-1.739776	2.104584
Cl	2.210086	-3.228697	2.381107
C	1.810555	-0.796053	1.226958
H	2.738283	-0.956460	0.694099
C	1.031283	0.369006	0.951416
H	1.528522	1.202344	0.471609
C	-0.106042	0.651930	1.790178
N	-0.762276	1.882897	1.634365
C	-0.231516	3.172825	1.571612
C	1.212152	3.506172	1.772324
H	1.271014	4.550488	2.083992
H	1.670003	2.882046	2.546237
H	1.802307	3.400083	0.855178
N	-1.185228	4.059749	1.406013
C	-2.370747	3.353966	1.350540
H	-3.320717	3.852487	1.216192
C	-2.146475	2.014148	1.495242
C	-3.003425	0.801182	1.605055
H	-2.664044	0.032547	0.894138
H	-4.034942	1.060148	1.356051
N	-2.975936	0.290197	2.983207
C	-1.876612	-0.190136	3.437987
C	-1.911424	-0.715480	4.838279
C	-3.071759	-1.344779	5.320044
H	-3.907576	-1.448788	4.636900
C	-3.152025	-1.815206	6.627582
H	-4.057057	-2.306350	6.970934
C	-2.067163	-1.655349	7.494543
H	-2.122047	-2.019786	8.515891
C	-0.908026	-1.020844	7.050796
H	-0.052895	-0.864497	7.699308
C	-0.851041	-0.562568	5.739833
F	0.274577	0.072948	5.342620
H	5.496312	-1.811152	-1.729685
H	3.922726	-4.004302	-1.968016
H	4.602789	3.387824	-1.710521
H	2.387000	4.935555	-1.924892
H	-3.376871	-3.323908	-2.999512
H	-1.182051	-4.883840	-2.690239
H	-4.268128	1.864861	-2.952692
H	-2.725178	4.066069	-2.591319

$^{4}RC_{H1'1}$

H	2.386421	0.487701	-5.436216
S	1.596175	1.394346	-4.824656
N	2.689989	-0.077917	-2.513933
C	3.644279	-1.009397	-2.865744
C	3.317563	0.791015	-1.649523
C	4.902217	-0.697352	-2.233040
C	4.697347	0.411273	-1.472746
C	2.716581	1.870760	-1.023460
H	3.319554	2.440096	-0.326135
C	1.402395	2.278336	-1.218356
N	0.487464	1.644445	-2.018670
C	-0.664296	2.390331	-1.936394
C	-0.462927	3.527539	-1.072005
C	0.816847	3.451686	-0.616685
C	-1.860388	2.084208	-2.566981
H	-2.691290	2.765946	-2.415610
C	-2.091853	0.963840	-3.359386
C	-3.356870	0.654913	-3.980796
C	-1.807366	-0.903163	-4.456669
N	-1.164238	0.005477	-3.659884
C	-3.180559	-0.510458	-4.661458
C	-1.223889	-2.033347	-5.016570
H	-1.856199	-2.667070	-5.630352
C	0.102170	-2.414323	-4.864979
C	0.687367	-3.585127	-5.470923
N	1.043693	-1.732979	-4.135814
C	2.203795	-2.447571	-4.261554
C	3.421010	-2.109961	-3.675663
H	4.262418	-2.769800	-3.861922
Fe	0.725490	-0.120269	-2.964400
C	1.995801	-3.605358	-5.096552
O	0.303127	-1.014990	-1.666602

C	-1.453565	0.199604	3.601358
C	-2.706626	-0.424803	3.494054
H	-3.384860	-0.408739	4.339312
C	-3.083775	-1.060767	2.318303
Cl	-4.666088	-1.819558	2.216821
C	-2.227668	-1.098672	1.218846
H	-2.525501	-1.587512	0.298254
C	-0.984818	-0.480283	1.306497
H	-0.340836	-0.484379	0.433844
C	-0.584891	0.160882	2.486050
N	0.691629	0.779903	2.545498
C	1.915220	0.327440	2.072463
C	2.147841	-1.023788	1.476546
H	3.221803	-1.215788	1.500096
H	1.635033	-1.806975	2.044427
H	1.809598	-1.092296	0.436749
N	2.855081	1.231469	2.271616
C	2.241594	2.299772	2.890209
H	2.796660	3.179269	3.188044
C	0.908337	2.053597	3.072979
C	-0.208638	2.765782	3.753994
H	-1.074673	2.860908	3.079129
H	0.114515	3.774637	4.021996
N	-0.589462	2.061723	4.985172
C	-1.125260	0.899225	4.886749
C	-1.519944	0.243005	6.171988
C	-1.993245	1.024773	7.239350
H	-2.076278	2.094348	7.080490
C	-2.340173	0.452702	8.459930
H	-2.712700	1.079015	9.264449
C	-2.209459	-0.926563	8.645875
H	-2.478254	-1.380695	9.594872
C	-1.727190	-1.726533	7.611295
H	-1.596839	-2.797294	7.724293
C	-1.390294	-1.132552	6.400385
F	-0.898739	-1.926982	5.424675
H	5.398321	0.936058	-0.838138
H	5.810532	-1.271999	-2.356437
H	1.335595	4.114761	0.061627
H	-1.215789	4.272240	-0.850377
H	0.153782	-4.285966	-6.099047
H	2.760530	-4.326722	-5.351989
H	-3.897727	-1.061218	-5.255362
H	-4.249475	1.260414	-3.897247

$^{4}TS1_{H1'1}$

H	2.682495	2.211787	-43.606901
S	2.129986	3.384783	-43.234866
N	3.039444	2.534544	-40.546053
C	3.848841	1.428909	-40.539454
C	3.765520	3.544031	-39.970236
C	5.122265	1.747854	-39.936478
C	5.069155	3.060692	-39.581890
C	3.317703	4.843889	-39.780730
H	3.992987	5.535859	-39.290078
C	2.074985	5.330171	-40.161635
N	1.076864	4.603373	-40.764555
C	0.021685	5.461836	-40.923311
C	0.365900	6.771672	-40.427107
C	1.638623	6.688407	-39.951777
C	-1.215037	5.122563	-41.459145
H	-1.961722	5.907203	-41.526486
C	-1.569841	3.862484	-41.916966
C	-2.850856	3.539045	-42.500922
C	-1.480068	1.753957	-42.510680
N	-0.755491	2.758240	-41.918548
C	-2.794923	2.231856	-42.869760
C	-1.019548	0.470412	-42.758642
H	-1.703460	-0.222574	-43.237999
C	0.246675	-0.002755	-42.434566
C	0.692850	-1.355456	-42.659644
N	1.235946	0.729592	-41.833731
C	2.290709	-0.132201	-41.650144
C	3.502077	0.183872	-41.048383
H	4.241090	-0.607738	-40.975840
Fe	1.102996	2.636484	-41.204421
C	1.960812	-1.436264	-42.170476
O	0.529430	2.173755	-39.653937
C	-1.472895	2.524698	-34.620410
C	-2.670508	1.792893	-34.592938
H	-3.186684	1.648884	-33.650911
C	-3.198763	1.254488	-35.759268
Cl	-4.706274	0.356235	-35.689026
C	-2.551149	1.422418	-36.982492
H	-2.972157	1.006300	-37.890734
C	-1.365189	2.147992	-37.030382
H	-0.874235	2.301466	-37.986881
C	-0.816962	2.693644	-35.861891
N	0.395622	3.430608	-35.938271
C	1.564751	3.148661	-36.651337
C	1.785192	1.964727	-37.447436

H	2.841202	1.804083	-37.653010
H	1.264448	1.066891	-37.113177
H	1.244122	2.125172	-38.637563
N	2.447043	4.139388	-36.494209
C	1.863971	5.048689	-35.666541
H	2.375694	5.949588	-35.354752
C	0.595252	4.645893	-35.302744
C	-0.434813	5.170015	-34.364488
H	-1.411329	5.245426	-34.869226
H	-0.151846	6.172533	-34.035534
N	-0.526327	4.302855	-33.182350
C	-0.976829	3.109389	-33.331790
C	-1.081348	2.282549	-32.090268
C	-1.415974	2.893209	-30.869661
H	-1.611475	3.959885	-30.877489
C	-1.492112	2.159915	-29.689134
H	-1.763009	2.654544	-28.761711
C	-1.221327	0.788528	-29.701303
H	-1.278876	0.209501	-28.784564
C	-0.870590	0.157468	-30.893647
H	-0.637820	-0.900859	-30.938150
C	-0.804479	0.910013	-32.060508
F	-0.434696	0.280237	-33.196713
H	5.828489	3.661635	-39.100071
H	5.937676	1.047604	-39.811879
H	2.241148	7.462478	-39.495624
H	-0.294087	7.628897	-40.443644
H	0.098248	-2.130397	-43.124994
H	2.623610	-2.291320	-42.151840
H	-3.557347	1.631893	-43.348779
H	-3.668761	4.238303	-42.614096

^{4}IM$_{H1'1}$

H	1.694052	0.191077	-5.720338
S	1.420254	1.239848	-4.917935
N	2.828670	0.034125	-2.468065
C	3.763008	-0.921539	-2.780230
C	3.493401	1.012787	-1.770909
C	5.044603	-0.552962	-2.231449
C	4.876545	0.643425	-1.603987
C	2.938886	2.199809	-1.318224
H	3.582026	2.872215	-0.762314
C	1.621729	2.587485	-1.514390
N	0.657052	1.865856	-2.175382
C	-0.504700	2.588562	-2.078373
C	-0.261065	3.814202	-1.361160
C	1.054786	3.812148	-1.009879
C	-1.746974	2.194475	-2.555906
H	-2.580022	2.872946	-2.406438
C	-2.002217	0.997746	-3.205474
C	-3.294538	0.610642	-3.716686
C	-1.741274	-0.950133	-4.187641
N	-1.076817	0.024251	-3.487659
C	-3.133338	-0.596088	-4.324029
C	-1.169950	-2.092910	-4.724682
H	-1.820647	-2.778836	-5.256385
C	0.174004	-2.424361	-4.632181
C	0.749098	-3.634937	-5.161448
N	1.143122	-1.680487	-4.006199
C	2.306766	-2.407843	-4.093609
C	3.528157	-2.061967	-3.534545
H	4.361892	-2.736714	-3.696935
Fe	0.871029	0.048308	-3.013526
C	2.069478	-3.624277	-4.828828
O	0.478477	-0.801922	-1.480745
C	-1.593921	0.109173	3.618252
C	-2.871634	-0.469363	3.560255
H	-3.511585	-0.439335	4.434448
C	-3.322086	-1.076149	2.394847
Cl	-4.931839	-1.778617	2.354633
C	-2.517573	-1.126442	1.256983
H	-2.879438	-1.586882	0.344579
C	-1.249072	-0.556784	1.294525
H	-0.642380	-0.560669	0.393045
C	-0.775918	0.051310	2.465629
N	0.520726	0.625313	2.478310
C	1.728920	0.122712	1.947552
C	1.918457	-1.143672	1.412499
H	2.919793	-1.414549	1.104759
H	1.134912	-1.888220	1.392023
H	1.278018	-0.860870	-0.927872
N	2.709675	1.048460	2.106514
C	2.145616	2.096187	2.743881
H	2.715867	2.976116	3.013776
C	0.799127	1.878341	2.991744
C	-0.257237	2.629029	3.723710
H	-1.147682	2.753614	3.086546
H	0.112082	3.625087	3.978675
N	-0.602529	1.930324	4.969161
C	-1.184520	0.787655	4.890727
C	-1.545975	0.141215	6.189795

C	-1.938231	0.935809	7.280497
H	-1.985712	2.008720	7.129935
C	-2.252638	0.371113	8.513096
H	-2.562860	1.007167	9.336125
C	-2.169780	-1.013450	8.687270
H	-2.413629	-1.461730	9.645703
C	-1.768019	-1.826568	7.628864
H	-1.676574	-2.902333	7.731762
C	-1.462525	-1.240050	6.406095
F	-1.049031	-2.047600	5.405870
H	5.608799	1.237813	-1.074552
H	5.946202	-1.143103	-2.328785
H	1.606815	4.558225	-0.454129
H	-1.013974	4.563208	-1.154237
H	0.195924	-4.390114	-5.703960
H	2.825031	-4.368997	-5.041402
H	-3.873265	-1.201373	-4.830776
H	-4.194660	1.203189	-3.620210

$^{4}TS2_{H1'1}$

H	1.244788	-0.418171	-5.815996
S	0.851398	0.752823	-5.275660
N	2.775546	0.759118	-2.770858
C	3.934043	0.057205	-3.002758
C	3.154466	2.043480	-2.466882
C	5.075355	0.915053	-2.800803
C	4.592941	2.143804	-2.467110
C	2.292487	3.106611	-2.238539
H	2.735081	4.069192	-2.005283
C	0.907759	3.040439	-2.312325
N	0.170296	1.919038	-2.604426
C	-1.149265	2.294040	-2.546365
C	-1.245746	3.696529	-2.226928
C	0.027562	4.157285	-2.078512
C	-2.241626	1.454614	-2.724759
H	-3.229703	1.898153	-2.657833
C	-2.164770	0.093436	-2.985475
C	-3.308253	-0.767305	-3.176227
C	-1.383483	-1.910730	-3.424118
N	-1.008269	-0.627188	-3.123499
C	-2.823966	-2.010267	-3.444440
C	-0.522906	-2.958680	-3.716223
H	-0.966075	-3.924541	-3.934940
C	0.861965	-2.865275	-3.764943
C	1.741459	-3.971256	-4.052412
N	1.598623	-1.731884	-3.531110
C	2.917669	-2.097814	-3.629069
C	4.009189	-1.270731	-3.396975
H	4.997434	-1.698619	-3.527892
Fe	0.878154	0.078877	-2.994981
C	3.015043	-3.495280	-3.970776
O	0.881149	-0.433769	-1.188998
C	-1.192544	0.759025	3.749636
C	-2.429023	1.405861	3.596756
H	-3.018976	1.643992	4.474164
C	-2.900590	1.742131	2.334398
Cl	-4.445230	2.565095	2.181692
C	-2.165465	1.437528	1.189154
H	-2.533223	1.706515	0.205904
C	-0.937142	0.798525	1.318753
H	-0.350215	0.572992	0.431206
C	-0.446544	0.455831	2.586848
N	0.814156	-0.184763	2.689053
C	1.375378	-1.178633	1.859468
C	0.717400	-1.850228	0.837847
H	1.259299	-2.623497	0.313291
H	-0.337388	-1.733787	0.647279
H	1.777407	-0.248492	-0.866395
N	2.655023	-1.414482	2.237271
C	2.894136	-0.610794	3.293697
H	3.847648	-0.606696	3.806077
C	1.786120	0.166668	3.602335
C	1.480312	1.158244	4.669624
H	1.141772	2.106097	4.220328
H	2.386564	1.366888	5.242808
N	0.463404	0.626280	5.585538
C	-0.727115	0.442940	5.139118
C	-1.732099	-0.055892	6.127569
C	-1.662139	0.363574	7.466978
H	-0.879120	1.064376	7.734970
C	-2.562163	-0.103114	8.420519
H	-2.492475	0.245114	9.446181
C	-3.554091	-1.017564	8.054451
H	-4.260011	-1.386261	8.792490
C	-3.638478	-1.465079	6.737036
H	-4.384042	-2.186342	6.420636
C	-2.730898	-0.982014	5.801567
F	-2.816212	-1.452933	4.538668
H	5.145272	3.048833	-2.251412
H	6.105183	0.602740	-2.912740
H	0.357392	5.157901	-1.832212

H	-2.175315	4.241143	-2.126276
H	1.410775	-4.976828	-4.276574
H	3.945145	-4.029675	-4.112250
H	-3.374293	-2.918546	-3.651645
H	-4.338771	-0.443020	-3.116077

${}^{4}PC_{H1'1}$

H	0.821913	-1.084691	-5.526292
S	0.456529	0.186169	-5.256486
N	2.827142	0.773441	-3.030509
C	3.918249	-0.025875	-3.240277
C	3.242053	2.070916	-3.163834
C	5.085849	0.799234	-3.462642
C	4.666739	2.097915	-3.415646
C	2.391351	3.178192	-3.117681
H	2.853284	4.154587	-3.229549
C	1.002377	3.166780	-2.961570
N	0.210440	2.053945	-2.813976
C	-1.081256	2.487204	-2.641608
C	-1.107485	3.933749	-2.691671
C	0.174390	4.351272	-2.888551
C	-2.198178	1.678960	-2.408163
H	-3.149225	2.192551	-2.301356
C	-2.222262	0.284888	-2.317614
C	-3.400635	-0.538119	-2.138991
C	-1.552465	-1.811108	-2.392906
N	-1.121468	-0.515077	-2.439732
C	-2.985626	-1.837186	-2.185131
C	-0.723668	-2.921308	-2.573508
H	-1.192403	-3.898924	-2.511990
C	0.648701	-2.909846	-2.838649
C	1.467745	-4.092281	-2.983917
N	1.444487	-1.792458	-2.956073
C	2.735185	-2.227642	-3.158684
C	3.870197	-1.421666	-3.284102
H	4.810688	-1.939813	-3.445392
Fe	0.804037	0.127995	-2.979548
C	2.749567	-3.673105	-3.180684
O	1.292739	0.258614	-0.668894
C	-1.082524	0.836420	3.755821
C	-2.298614	1.535889	3.704318
H	-2.961595	1.515465	4.561442
C	-2.661026	2.249043	2.568626
Cl	-4.184394	3.122512	2.547052
C	-1.832572	2.279668	1.448008
H	-2.117396	2.834302	0.561659
C	-0.620717	1.598469	1.483807
H	0.038647	1.628549	0.623489
C	-0.238220	0.876989	2.622352
N	1.008659	0.196258	2.625388
C	1.623980	-0.528604	1.613876
C	1.043755	-0.812075	0.268030
H	1.482904	-1.742955	-0.098992
H	-0.041518	-0.919443	0.299108
H	2.251648	0.361936	-0.778155
N	2.815937	-0.948265	1.990356
C	2.994068	-0.497433	3.275307
H	3.894117	-0.712348	3.834715
C	1.898037	0.211451	3.695729
C	1.497023	0.851509	4.980397
H	1.208687	1.901717	4.813311
H	2.347351	0.847384	5.666210
N	0.396721	0.105499	5.602199
C	-0.746810	0.100066	5.019019
C	-1.840603	-0.646238	5.714271
C	-1.897109	-0.655680	7.118312
H	-1.142998	-0.091704	7.656213
C	-2.882105	-1.365142	7.799310
H	-2.909093	-1.347212	8.884242
C	-3.834494	-2.097362	7.084555
H	-4.606137	-2.653172	7.608584
C	-3.794355	-2.120984	5.691376
H	-4.506202	-2.691148	5.104505
C	-2.803274	-1.401903	5.033593
F	-2.768851	-1.464561	3.684273
H	5.257996	2.993865	-3.553906
H	6.085784	0.427817	-3.645672
H	0.542214	5.365746	-2.969967
H	-1.998593	4.538344	-2.583097
H	1.098079	-5.107726	-2.926495
H	3.637131	-4.277296	-3.315485
H	-3.587317	-2.732935	-2.101588
H	-4.408043	-0.164191	-2.009859

${}^{4}RC_{H41}$

H	-0.695991	1.739324	-4.882701
S	-0.334092	0.450791	-5.053857
N	1.962697	-0.515029	-2.878501
C	2.532526	-1.708233	-3.223062
C	3.002247	0.353548	-2.679178

C	3.971531	-1.595816	-3.240211
C	4.264054	-0.310402	-2.903953
C	2.873791	1.688126	-2.316482
H	3.788199	2.261435	-2.199488
C	1.674454	2.347792	-2.079311
N	0.427793	1.787240	-2.189214
C	-0.459536	2.772938	-1.851008
C	0.240699	3.990285	-1.520513
C	1.569754	3.727108	-1.669005
C	-1.845601	2.642233	-1.855422
H	-2.427706	3.501175	-1.539665
C	-2.540016	1.501289	-2.220997
C	-3.978037	1.391657	-2.242485
C	-3.012823	-0.557875	-2.823062
N	-1.964665	0.306599	-2.595609
C	-4.270584	0.117410	-2.616010
C	-2.885731	-1.891531	-3.174994
H	-3.799960	-2.460505	-3.309965
C	-1.683771	-2.556010	-3.385671
C	-1.576562	-3.939546	-3.782788
N	-0.439116	-1.998205	-3.255320
C	0.453794	-2.998705	-3.554991
C	1.833671	-2.869580	-3.539137
H	2.418492	-3.746226	-3.798963
Fe	0.001194	-0.146050	-2.586099
C	-0.249014	-4.213779	-3.890251
O	0.044692	-0.586993	-1.018684
C	0.330057	-0.114227	3.069823
C	1.597928	-0.674593	3.291459
H	1.685254	-1.576423	3.886066
C	2.737751	-0.088964	2.757108
Cl	4.307859	-0.821977	3.049915
C	2.651687	1.068910	1.986071
H	3.543627	1.509741	1.555974
C	1.402259	1.632154	1.754931
H	1.319448	2.504181	1.118157
C	0.242063	1.064234	2.295215
N	-1.012170	1.676735	2.047151
C	-1.355170	3.022136	2.053621
C	-0.437891	4.135749	2.448289
H	-1.055006	4.988981	2.735462
H	0.201073	3.859519	3.292633
H	0.215106	4.455523	1.627455
N	-2.619097	3.185407	1.721203
C	-3.123959	1.922351	1.494424
H	-4.156482	1.764703	1.214703
C	-2.159632	0.971127	1.684614
C	-2.129111	-0.518193	1.659983
H	-1.325620	-0.870359	0.995171
H	-3.076681	-0.889998	1.263039
N	-1.964184	-1.044010	3.022988
C	-0.861407	-0.825516	3.640594
C	-0.745400	-1.410435	5.013555
C	-1.344547	-2.649011	5.298052
H	-1.859871	-3.154584	4.488753
C	-1.285772	-3.208464	6.571397
H	-1.749068	-4.171733	6.760761
C	-0.629223	-2.529803	7.602060
H	-0.578637	-2.959522	8.598009
C	-0.038589	-1.291599	7.354834
H	0.465210	-0.728405	8.132892
C	-0.108191	-0.755354	6.074171
F	0.449487	0.458975	5.868809
H	5.233936	0.160149	-2.812825
H	4.650516	-2.401878	-3.484255
H	2.409939	4.394047	-1.526657
H	-0.238425	4.916052	-1.231271
H	-2.418156	-4.596656	-3.956777
H	0.228124	-5.143534	-4.170065
H	-5.241831	-0.342124	-2.742301
H	-4.657278	2.196387	-1.995407

$^{4}TS1_{H41}$

H	0.964185	3.845439	-43.199550
S	1.041633	2.503482	-43.088524
N	3.187274	1.911486	-40.749067
C	3.737222	0.660279	-40.848798
C	4.243213	2.785522	-40.678831
C	5.179614	0.743736	-40.820651
C	5.493358	2.061617	-40.716254
C	4.142293	4.165481	-40.604681
H	5.067435	4.731075	-40.557947
C	2.951688	4.884881	-40.576561
N	1.697904	4.341048	-40.638421
C	0.818904	5.390993	-40.528856
C	1.543139	6.633286	-40.412090
C	2.868448	6.319360	-40.446200
C	-0.567142	5.292641	-40.531819
H	-1.130344	6.215518	-40.441666
C	-1.290113	4.111605	-40.648538
C	-2.731645	4.030508	-40.688363

C	-1.796351	1.988120	-40.843645
N	-0.743269	2.859587	-40.740907
C	-3.045919	2.711365	-40.808433
C	-1.683956	0.609939	-40.969996
H	-2.604724	0.038883	-41.032783
C	-0.492773	-0.099464	-41.036793
C	-0.411808	-1.533997	-41.167839
N	0.769604	0.439382	-40.974928
C	1.639298	-0.616474	-41.049035
C	3.023882	-0.520842	-40.990274
H	3.591748	-1.442677	-41.063252
Fe	1.247758	2.370885	-40.696655
C	0.910528	-1.854882	-41.177239
O	1.221297	2.253916	-38.999302
C	1.846086	2.882252	-35.816693
C	3.144131	2.363437	-35.694073
H	3.279702	1.343633	-35.354569
C	4.252891	3.135029	-36.015124
Cl	5.862056	2.451622	-35.850564
C	4.103942	4.441375	-36.476086
H	4.971960	5.031983	-36.745484
C	2.822701	4.966385	-36.604223
H	2.694232	5.963149	-37.006965
C	1.694720	4.211624	-36.264536
N	0.406444	4.798576	-36.393366
C	0.017561	6.102815	-36.129407
C	0.850030	7.121040	-35.418293
H	0.171913	7.833185	-34.944610
H	1.486146	6.667457	-34.653234
H	1.497341	7.686304	-36.099406
N	-1.222079	6.319806	-36.531683
C	-1.657227	5.143705	-37.080902
H	-2.657852	5.030537	-37.474636
C	-0.682856	4.170271	-37.001943
C	-0.668984	2.745001	-37.330893
H	0.221366	2.524639	-38.230587
H	-1.599505	2.442620	-37.814611
N	-0.371717	1.872161	-36.238987
C	0.699429	1.965197	-35.532732
C	0.823995	0.987279	-34.411312
C	0.314693	-0.315362	-34.554262
H	-0.135012	-0.577469	-35.505602
C	0.384322	-1.238286	-33.514993
H	-0.005954	-2.241295	-33.656119
C	0.958861	-0.873503	-32.293707
H	1.016430	-1.588382	-31.478460
C	1.458596	0.415854	-32.116217
H	1.897106	0.739359	-31.178477
C	1.381463	1.318827	-33.169704
F	1.848895	2.570653	-32.964149
H	6.471635	2.521476	-40.671623
H	5.845841	-0.106585	-40.880889
H	3.720959	6.983658	-40.390852
H	1.081238	7.607498	-40.320769
H	-1.266639	-2.193232	-41.240693
H	1.367653	-2.832117	-41.258621
H	-4.024369	2.254489	-40.876290
H	-3.398125	4.881341	-40.637058

$^{4}IM_{H41}$

H	0.536866	-0.005829	-5.053715
S	-0.329279	-1.012151	-4.817831
N	1.522608	-2.357669	-2.726500
C	1.494252	-3.720433	-2.859941
C	2.841669	-1.990065	-2.767046
C	2.839748	-4.232477	-2.970555
C	3.674867	-3.160646	-2.910041
C	3.321692	-0.690627	-2.717263
H	4.396534	-0.549956	-2.757640
C	2.529967	0.445830	-2.629986
N	1.158730	0.468088	-2.576713
C	0.796445	1.787767	-2.457512
C	1.972180	2.621877	-2.468022
C	3.045506	1.790719	-2.571313
C	-0.498439	2.265239	-2.315888
H	-0.632526	3.340026	-2.255848
C	-1.634149	1.471677	-2.242159
C	-2.978810	1.980385	-2.139230
C	-2.976528	-0.270871	-2.235833
N	-1.654414	0.098730	-2.275375
C	-3.810528	0.900815	-2.132141
C	-3.458036	-1.568782	-2.330504
H	-4.531635	-1.711241	-2.268813
C	-2.672800	-2.695140	-2.525016
C	-3.189210	-4.038487	-2.613946
N	-1.305981	-2.711389	-2.649885
C	-0.944356	-4.026476	-2.793722
C	0.353046	-4.506339	-2.898552
H	0.483567	-5.577379	-3.010124
Fe	-0.062540	-1.134909	-2.516807

C	-2.119228	-4.862427	-2.782592
O	0.122026	-1.304356	-0.754521
C	0.286218	1.923096	3.134370
C	1.648205	2.241774	3.311950
H	2.390213	1.462998	3.185393
C	2.053425	3.532330	3.620826
Cl	3.762677	3.874800	3.833687
C	1.126147	4.565746	3.738516
H	1.448480	5.578428	3.950570
C	-0.222628	4.270584	3.560023
H	-0.948157	5.071899	3.618537
C	-0.655115	2.967600	3.294790
N	-2.056557	2.717542	3.179933
C	-3.091723	3.374204	3.821488
C	-2.954722	4.213556	5.052216
H	-3.873158	4.102550	5.632130
H	-2.102957	3.908270	5.664139
H	-2.843501	5.279599	4.822041
N	-4.258228	3.108355	3.253807
C	-3.990239	2.276147	2.207885
H	-4.774194	1.877337	1.577966
C	-2.638718	1.984617	2.138775
C	-1.972378	0.986614	1.357027
H	-0.422839	-0.708354	-0.205196
H	-2.505089	0.665336	0.464731
N	-0.899997	0.303297	1.669557
C	-0.043458	0.554104	2.688576
C	0.722643	-0.594503	3.190115
C	1.014394	-1.712669	2.374564
H	0.716197	-1.694043	1.332663
C	1.701469	-2.813503	2.874810
H	1.922318	-3.646057	2.213950
C	2.111463	-2.849122	4.211123
H	2.646459	-3.708926	4.602877
C	1.825625	-1.770405	5.048583
H	2.108089	-1.762562	6.095837
C	1.142566	-0.678768	4.533852
F	0.851009	0.332499	5.386037
H	4.755338	-3.141886	-2.963638
H	3.090564	-5.278937	-3.083096
H	4.096469	2.046192	-2.599393
H	1.959190	3.701079	-2.392157
H	-4.237486	-4.297973	-2.548103
H	-2.105970	-5.939622	-2.882611
H	-4.891307	0.882309	-2.082188
H	-3.235964	3.030136	-2.089452

$^{4}TS2_{H41}$

H	1.136577	-2.128548	-4.593582
S	-0.096774	-1.612935	-4.416287
N	-0.398752	-2.800954	-1.483522
C	-1.681277	-3.235079	-1.278831
C	0.420211	-3.867195	-1.223419
C	-1.672389	-4.614702	-0.851840
C	-0.369117	-5.006674	-0.816322
C	1.802503	-3.875753	-1.356621
H	2.323568	-4.795635	-1.112302
C	2.570906	-2.801528	-1.786559
N	2.094905	-1.566416	-2.149054
C	3.191836	-0.798252	-2.447979
C	4.393955	-1.581623	-2.301327
C	4.009314	-2.821984	-1.890602
C	3.170393	0.543411	-2.808177
H	4.121075	1.015803	-3.032189
C	2.027865	1.324810	-2.910060
C	2.014627	2.700752	-3.343068
C	-0.077765	1.961774	-2.952795
N	0.746058	0.904106	-2.652493
C	0.711552	3.094749	-3.369648
C	-1.463936	1.944898	-2.906259
H	-1.983878	2.860201	-3.168720
C	-2.238977	0.843397	-2.563420
C	-3.679137	0.852208	-2.492395
N	-1.763658	-0.395252	-2.217540
C	-2.857554	-1.164048	-1.910230
C	-2.830416	-2.481782	-1.471992
H	-3.782214	-2.959775	-1.266318
Fe	0.168234	-0.962130	-2.112693
C	-4.062484	-0.391482	-2.086112
O	0.313423	-0.459898	-0.284699
C	-0.648212	1.780575	2.285636
C	-0.544356	3.133866	1.911329
H	0.355410	3.684419	2.157602
C	-1.566207	3.763204	1.214538
Cl	-1.397340	5.452584	0.768106
C	-2.713816	3.064323	0.844251
H	-3.494672	3.550311	0.271156
C	-2.829138	1.724802	1.202981
H	-3.693629	1.163078	0.873818
C	-1.828808	1.082557	1.938508
N	-2.019188	-0.273127	2.329074

C	-3.205303	-0.910401	2.646601
C	-4.484839	-0.222832	3.002558
H	-5.021425	-0.862155	3.706335
H	-4.311835	0.753211	3.462404
H	-5.136102	-0.076372	2.133071
N	-3.055823	-2.225147	2.665407
C	-1.755701	-2.467070	2.327410
H	-1.353638	-3.469489	2.275812
C	-1.070821	-1.284003	2.129319
C	0.327358	-1.038273	1.956893
H	0.653158	0.448788	-0.271637
H	0.926762	-1.873803	1.615891
N	0.986532	-0.048173	2.552333
C	0.527068	1.137888	2.913557
C	1.383303	1.888331	3.851820
C	2.771641	1.635916	3.904815
H	3.173622	0.907622	3.209853
C	3.597925	2.296961	4.805131
H	4.663581	2.090400	4.809889
C	3.059343	3.228844	5.699123
H	3.699666	3.748586	6.405124
C	1.689293	3.488309	5.687977
H	1.230722	4.188376	6.377863
C	0.880811	2.819944	4.778493
F	-0.447178	3.068229	4.821727
H	0.037201	-5.974861	-0.554385
H	-2.557752	-5.192587	-0.621919
H	4.629301	-3.680196	-1.666935
H	5.394467	-1.211822	-2.483272
H	-4.301079	1.705318	-2.730548
H	-5.063431	-0.769116	-1.922538
H	0.303955	4.055966	-3.653577
H	2.896518	3.271730	-3.602064

$^{4}PC_{H41}$

H	0.972970	-3.545759	-4.204543
S	1.135361	-2.258271	-4.573405
N	-0.131770	-3.021376	-1.554315
C	-1.198891	-3.892093	-1.621282
C	0.795041	-3.631995	-0.733722
C	-0.931224	-5.071040	-0.843304
C	0.305357	-4.909977	-0.293037
C	2.014855	-3.093413	-0.351654
H	2.647599	-3.692290	0.294498
C	2.480153	-1.843855	-0.728076
N	1.800195	-0.947391	-1.529307
C	2.655612	0.125440	-1.691161
C	3.868725	-0.086308	-0.949399
C	3.760245	-1.307241	-0.352594
C	2.395338	1.243415	-2.467429
H	3.150352	2.021584	-2.499434
C	1.236869	1.441336	-3.203495
C	0.962768	2.628458	-3.968504
C	-0.754385	1.180710	-4.090276
N	0.180481	0.560503	-3.291384
C	-0.272786	2.466843	-4.518263
C	-1.998237	0.667044	-4.422798
H	-2.636295	1.268924	-5.060994
C	-2.488998	-0.553480	-3.989426
C	-3.789457	-1.070343	-4.334227
N	-1.816384	-1.444099	-3.185783
C	-2.662934	-2.517263	-3.032308
C	-2.379737	-3.666307	-2.312597
H	-3.139494	-4.439716	-2.276637
Fe	0.107573	-1.322336	-2.598894
C	-3.897642	-2.289105	-3.740038
O	-0.532540	0.232742	0.211125
C	-0.337213	2.144224	2.484318
C	0.279179	3.390319	2.300269
H	1.351058	3.479428	2.431829
C	-0.465137	4.508221	1.944299
Cl	0.345239	6.048895	1.707731
C	-1.843896	4.419765	1.766926
H	-2.418357	5.291746	1.475806
C	-2.466898	3.189208	1.947729
H	-3.532077	3.101742	1.773700
C	-1.736386	2.053269	2.313307
N	-2.410825	0.820608	2.495258
C	-3.633518	0.574522	3.108185
C	-4.461539	1.600755	3.814658
H	-5.104948	1.074775	4.521985
H	-3.845979	2.322138	4.359993
H	-5.111233	2.162817	3.133130
N	-3.950358	-0.701345	3.036100
C	-2.923448	-1.314988	2.357626
H	-2.923232	-2.379379	2.165930
C	-1.958052	-0.409483	2.013822
C	-0.614409	-0.555975	1.391583
H	0.318277	0.031653	-0.212680
H	-0.472147	-1.613663	1.144504
N	0.432428	-0.219709	2.370895

C	0.530969	0.974060	2.834119
C	1.675920	1.213633	3.768474
C	2.920443	0.611654	3.519382
H	3.017595	0.006913	2.624017
C	3.995687	0.787315	4.386484
H	4.950807	0.320915	4.165874
C	3.843888	1.564427	5.538288
H	4.677464	1.704462	6.219895
C	2.616124	2.162138	5.819749
H	2.457326	2.758640	6.711515
C	1.559545	1.977785	4.935871
F	0.372437	2.545897	5.241772
H	0.844195	-5.581557	0.362026
H	-1.614452	-5.902659	-0.733094
H	4.478533	-1.816065	0.276551
H	4.692862	0.613371	-0.907185
H	-4.512065	-0.553505	-4.951579
H	-4.727759	-2.982367	-3.766632
H	-0.823814	3.149725	-5.151054
H	1.635059	3.471504	-4.056234

$^{4}RC_{H42}$

H	1.442348	2.604509	-4.520403
S	1.453314	1.261256	-4.646953
N	3.796082	-0.137185	-2.776834
C	4.008167	-1.436016	-3.146585
C	5.029497	0.455857	-2.763722
C	5.412976	-1.673419	-3.375004
C	6.049689	-0.494795	-3.134484
C	5.275372	1.788622	-2.455587
H	6.307533	2.123306	-2.485257
C	4.312424	2.728053	-2.114493
N	2.964129	2.486838	-2.035214
C	2.391987	3.675664	-1.671761
C	3.400084	4.696908	-1.522815
C	4.595019	4.107146	-1.799356
C	1.028231	3.884171	-1.482355
H	0.713016	4.880580	-1.190265
C	0.041968	2.922797	-1.624623
C	-1.364652	3.153413	-1.404594
C	-0.973715	0.999623	-1.930124
N	0.262572	1.604731	-1.963419
C	-1.992216	1.962236	-1.589613
C	-1.213067	-0.341338	-2.177099
H	-2.235640	-0.691088	-2.087034
C	-0.250579	-1.272505	-2.547273
C	-0.532839	-2.650334	-2.867724
N	1.088618	-1.016677	-2.677977
C	1.662097	-2.203242	-3.068147
C	3.015818	-2.400646	-3.292395
H	3.329638	-3.393795	-3.598027
Fe	2.045922	0.698768	-2.221128
C	0.654241	-3.227255	-3.197537
O	2.249374	0.318481	-0.649558
C	-2.140263	-1.293591	3.500184
C	-2.789678	-2.260044	4.284311
H	-3.270717	-3.102422	3.800751
C	-2.824780	-2.150138	5.668464
Cl	-3.638436	-3.384570	6.616157
C	-2.217021	-1.074116	6.313660
H	-2.237401	-0.999288	7.394980
C	-1.564857	-0.112987	5.549658
H	-1.058232	0.706827	6.044024
C	-1.523496	-0.199515	4.151158
N	-0.853747	0.802060	3.410114
C	-0.842006	2.181402	3.590545
C	-1.677979	2.920483	4.587116
H	-1.768339	3.952224	4.242928
H	-2.679036	2.489250	4.685680
H	-1.226619	2.945800	5.586561
N	-0.038899	2.759663	2.724293
C	0.499825	1.749039	1.954561
H	1.201511	1.938121	1.155183
C	0.014809	0.532865	2.346857
C	0.162233	-0.857156	1.833761
H	0.480342	-1.541626	2.636874
H	0.925013	-0.862265	1.053187
N	-1.105007	-1.324915	1.249801
C	-2.121444	-1.491085	2.012786
C	-3.364871	-2.003785	1.353963
C	-3.268094	-2.943354	0.313329
H	-2.276951	-3.288284	0.038793
C	-4.400741	-3.415352	-0.345618
H	-4.299907	-4.152961	-1.135948
C	-5.666514	-2.946120	0.018231
H	-6.554407	-3.310443	-0.489605
C	-5.793041	-2.004093	1.037858
H	-6.756732	-1.607302	1.338267
C	-4.647823	-1.551094	1.683401
F	-4.795985	-0.619395	2.650793
H	7.106165	-0.271438	-3.200987

H	5.837089	-2.620832	-3.679964
H	5.582775	4.548494	-1.795567
H	3.201111	5.722846	-1.243255
H	-1.518760	-3.094803	-2.846804
H	0.848939	-4.247233	-3.501341
H	-3.045888	1.733856	-1.502373
H	-1.793732	4.107331	-1.129212

$^{4}TS1_{H42}$

H	1.007534	4.521303	-42.935533
S	1.051031	3.181592	-43.087065
N	3.248313	1.855374	-41.162773
C	3.540369	0.559227	-41.506611
C	4.449888	2.511904	-41.101472
C	4.968214	0.391267	-41.647711
C	5.532054	1.603253	-41.400721
C	4.614975	3.855808	-40.801228
H	5.627611	4.245778	-40.790148
C	3.587357	4.736954	-40.491034
N	2.253779	4.419470	-40.456334
C	1.599946	5.562930	-40.060048
C	2.549454	6.628532	-39.850470
C	3.781199	6.117977	-40.123379
C	0.228306	5.695012	-39.888545
H	-0.140247	6.662769	-39.564702
C	-0.710746	4.696111	-40.114781
C	-2.139939	4.869268	-39.996378
C	-1.623596	2.781318	-40.660352
N	-0.419963	3.419861	-40.513835
C	-2.706452	3.680756	-40.338278
C	-1.788131	1.461594	-41.054965
H	-2.802833	1.083696	-41.117115
C	-0.761087	0.579685	-41.365475
C	-0.960753	-0.797027	-41.747100
N	0.579835	0.866355	-41.328263
C	1.229830	-0.291223	-41.659756
C	2.610250	-0.443408	-41.733735
H	2.989481	-1.422281	-42.009169
Fe	1.441515	2.617256	-40.790578
C	0.275636	-1.339341	-41.926711
O	1.555180	2.159242	-39.141642
C	-2.334155	1.025597	-35.408962
C	-3.029169	0.049016	-34.677498
H	-3.573587	-0.724584	-35.205528
C	-3.020953	0.054105	-33.288466
Cl	-3.897657	-1.187499	-32.411584
C	-2.313656	1.027962	-32.587203
H	-2.291302	1.018873	-31.503404
C	-1.617446	1.997535	-33.300618
H	-1.034947	2.733399	-32.761253
C	-1.628601	2.024373	-34.701884
N	-0.919682	3.046688	-35.378996
C	-0.742090	4.370215	-34.988396
C	-1.517858	5.063248	-33.911698
H	-1.542846	6.126635	-34.157169
H	-2.542928	4.690166	-33.835874
H	-1.051812	4.968763	-32.923524
N	0.150992	4.970887	-35.745230
C	0.588040	4.032198	-36.648955
H	1.317030	4.260477	-37.411744
C	-0.054664	2.833531	-36.465836
C	-0.040077	1.585664	-37.254571
H	0.643210	0.790249	-36.928657
H	0.657335	1.909476	-38.340927
N	-1.296666	1.068852	-37.645603
C	-2.321460	0.888400	-36.899408
C	-3.551543	0.362352	-37.569355
C	-3.449398	-0.611718	-38.575978
H	-2.460314	-0.974825	-38.833423
C	-4.581089	-1.096110	-39.228217
H	-4.479665	-1.862059	-39.990838
C	-5.845592	-0.601192	-38.895179
H	-6.731207	-0.973917	-39.400770
C	-5.975176	0.376597	-37.909665
H	-6.938351	0.791728	-37.633646
C	-4.832632	0.837673	-37.266197
F	-4.975498	1.796254	-36.325557
H	6.578536	1.877568	-41.416760
H	5.454384	-0.538568	-41.911934
H	4.742250	6.612668	-40.075923
H	2.288660	7.629274	-39.532658
H	-1.927701	-1.268749	-41.862114
H	0.534851	-2.349173	-42.216264
H	-3.755223	3.417626	-40.374250
H	-2.625410	5.787795	-39.694402

$^{4}IM_{H42}$

H	3.900778	1.756465	-4.340423
S	3.316348	0.554548	-4.520933
N	2.871875	-0.075112	-1.495897

C	3.035009	-1.412925	-1.239472
C	3.699544	0.589298	-0.626822
C	3.972942	-1.595890	-0.158686
C	4.383553	-0.356108	0.221463
C	3.889070	1.961195	-0.577813
H	4.570814	2.348583	0.171295
C	3.263968	2.877328	-1.411372
N	2.372648	2.574702	-2.411611
C	1.952409	3.776217	-2.931755
C	2.627604	4.861062	-2.265611
C	3.437863	4.304789	-1.323374
C	0.996912	3.945151	-3.923317
H	0.780276	4.959199	-4.241799
C	0.297933	2.916511	-4.537642
C	-0.652689	3.098650	-5.605500
C	-0.379302	0.912107	-5.139619
N	0.428858	1.579233	-4.250911
C	-1.072147	1.857741	-5.977792
C	-0.491860	-0.465699	-5.250608
H	-1.178912	-0.854598	-5.994427
C	0.214588	-1.385196	-4.489362
C	0.057494	-2.813757	-4.594020
N	1.133050	-1.087855	-3.511496
C	1.537688	-2.287033	-2.981071
C	2.422511	-2.449466	-1.925120
H	2.649295	-3.462853	-1.612075
Fe	1.690830	0.741623	-2.892916
C	0.876491	-3.371903	-3.660536
O	0.375072	0.832753	-1.664846
C	-1.791600	-1.386904	3.900778
C	-2.111312	-2.442253	4.780096
H	-2.977431	-3.057460	4.569916
C	-1.326144	-2.723109	5.888405
Cl	-1.773165	-4.049053	6.952242
C	-0.173055	-1.987930	6.155060
H	0.454763	-2.229206	7.004783
C	0.163858	-0.948299	5.292292
H	1.075742	-0.392890	5.470723
C	-0.639412	-0.616974	4.196419
N	-0.266908	0.496266	3.383635
C	0.408630	1.639952	3.773279
C	0.526922	2.138024	5.179506
H	0.544418	3.229279	5.143591
H	-0.309135	1.811861	5.802410
H	1.456174	1.814917	5.663614
N	0.882094	2.302909	2.729780
C	0.533279	1.578118	1.625988
H	0.769692	1.889167	0.617769
C	-0.202449	0.461293	1.983070
C	-0.962468	-0.416955	1.144352
H	-0.656599	-0.433947	0.098965
H	-0.410502	1.219260	-2.083701
N	-2.067613	-1.043899	1.455005
C	-2.603257	-1.230305	2.678640
C	-4.044207	-1.520412	2.681450
C	-4.674014	-2.045527	1.529006
H	-4.044654	-2.256544	0.672354
C	-6.041185	-2.289510	1.488227
H	-6.485730	-2.707012	0.589788
C	-6.842243	-2.004234	2.599365
H	-7.911063	-2.192999	2.573830
C	-6.259914	-1.465332	3.746757
H	-6.845777	-1.209389	4.623121
C	-4.892594	-1.228858	3.767905
F	-4.374664	-0.667572	4.885579
H	5.087591	-0.085369	0.996940
H	4.269761	-2.557326	0.238815
H	4.088219	4.799966	-0.614824
H	2.476114	5.908124	-2.491934
H	-0.606390	-3.307650	-5.291017
H	1.024129	-4.418957	-3.431908
H	-1.776456	1.588495	-6.753672
H	-0.941743	4.058619	-6.012280

$^{4}TS2_{H42}$

H	2.596692	1.925231	-4.727078
S	1.887249	0.780482	-4.801061
N	2.820527	-0.285687	-1.913052
C	2.998064	-1.643847	-1.952675
C	4.006616	0.250419	-1.489173
C	4.333520	-1.978318	-1.514337
C	4.959278	-0.803716	-1.227611
C	4.272356	1.606903	-1.350773
H	5.258755	1.891085	-0.999000
C	3.375269	2.631074	-1.629247
N	2.091561	2.474447	-2.092941
C	1.550230	3.733507	-2.174816
C	2.528778	4.714699	-1.776451
C	3.658664	4.032477	-1.438929
C	0.249735	4.036474	-2.559507
H	-0.029347	5.084496	-2.591040

C	-0.716616	3.105380	-2.915519
C	-2.044136	3.436534	-3.374456
C	-1.714916	1.207242	-3.403550
N	-0.546886	1.743789	-2.920222
C	-2.661745	2.261348	-3.677243
C	-1.952580	-0.141531	-3.627139
H	-2.930995	-0.420216	-4.004457
C	-1.035392	-1.164090	-3.411650
C	-1.318082	-2.565535	-3.605968
N	0.247674	-1.006308	-2.958266
C	0.779384	-2.263729	-2.838741
C	2.053313	-2.569864	-2.374032
H	2.334962	-3.617553	-2.348264
Fe	1.155854	0.734701	-2.476843
C	-0.193321	-3.247708	-3.248725
O	0.542492	0.580122	-0.623789
C	-1.226497	-1.251095	3.627274
C	-1.715372	-2.264469	4.471003
H	-2.270176	-3.089938	4.042065
C	-1.475539	-2.234112	5.837727
Cl	-2.102653	-3.516375	6.856045
C	-0.722896	-1.207175	6.404686
H	-0.512684	-1.200499	7.467964
C	-0.226691	-0.202633	5.579162
H	0.388962	0.575826	6.010789
C	-0.486601	-0.192574	4.203829
N	0.009542	0.884002	3.412869
C	0.204575	2.194014	3.821745
C	-0.443054	2.836219	5.008421
H	-0.614340	3.885884	4.761132
H	-1.396292	2.367215	5.262828
H	0.195792	2.814637	5.899021
N	0.982493	2.851603	2.979851
C	1.333307	1.964417	2.003707
H	1.923501	2.250507	1.145574
C	0.743578	0.735149	2.218226
C	0.650314	-0.423870	1.362288
H	1.524297	-0.745675	0.813495
H	-0.349500	0.958949	-0.580855
N	-0.409194	-1.219015	1.321507
C	-1.390796	-1.419119	2.164898
C	-2.613907	-2.019945	1.594748
C	-2.541453	-2.753764	0.390356
H	-1.565446	-2.887324	-0.061959
C	-3.677748	-3.290225	-0.201579
H	-3.584445	-3.856566	-1.122519
C	-4.932856	-3.099169	0.386321
H	-5.824493	-3.516651	-0.071261
C	-5.043117	-2.360661	1.563809
H	-6.000334	-2.171684	2.037292
C	-3.896148	-1.833356	2.141898
F	-4.046031	-1.087020	3.258368
H	5.972285	-0.646740	-0.880831
H	4.727336	-2.984363	-1.451899
H	4.601783	4.426017	-1.083148
H	2.354899	5.782426	-1.754055
H	-2.257861	-2.958530	-3.971557
H	-0.020601	-4.316032	-3.259627
H	-3.660758	2.105428	-4.062785
H	-2.431920	4.443109	-3.460220

$^{4}PC_{H42}$

H	2.844498	1.187669	-4.648365
S	2.220925	0.024602	-4.367228
N	2.833113	0.024511	-1.123471
C	3.051382	-1.253941	-0.693450
C	3.938228	0.758039	-0.798001
C	4.335172	-1.327962	-0.027275
C	4.884194	-0.080288	-0.090746
C	4.130113	2.094607	-1.155003
H	5.053696	2.561752	-0.826469
C	3.263327	2.898083	-1.899966
N	2.048528	2.520670	-2.419850
C	1.496235	3.626780	-3.020428
C	2.410716	4.740800	-2.894555
C	3.496499	4.293027	-2.204516
C	0.234101	3.701057	-3.616108
H	-0.034609	4.657666	-4.054190
C	-0.709358	2.675522	-3.712252
C	-1.978228	2.742159	-4.405123
C	-1.613761	0.670965	-3.580208
N	-0.535014	1.419528	-3.198238
C	-2.538274	1.499732	-4.323951
C	-1.749158	-0.697135	-3.330190
H	-2.654839	-1.173836	-3.693800
C	-0.828055	-1.530216	-2.687946
C	-1.030664	-2.941822	-2.444709
N	0.384234	-1.151055	-2.161900
C	0.952416	-2.267982	-1.594644
C	2.171551	-2.317256	-0.912674
H	2.465472	-3.287018	-0.522318

Fe	1.263940	0.665115	-2.367026
C	0.064104	-3.395229	-1.771690
O	0.186931	1.399436	-0.384883
C	-0.950334	-1.356356	2.656677
C	-1.079278	-2.738307	2.864723
H	-1.806742	-3.297824	2.288343
C	-0.284881	-3.393287	3.796940
Cl	-0.461918	-5.124171	4.022817
C	0.655474	-2.691615	4.550903
H	1.279689	-3.210496	5.269485
C	0.796130	-1.323120	4.350716
H	1.548372	-0.776855	4.906373
C	0.002044	-0.640808	3.419380
N	0.168682	0.755380	3.249294
C	0.357001	1.749960	4.205719
C	0.312692	1.531027	5.684572
H	0.091144	2.490827	6.154087
H	-0.457767	0.808603	5.970724
H	1.268867	1.178642	6.089901
N	0.524524	2.921612	3.634373
C	0.453258	2.710709	2.273357
H	0.564062	3.514523	1.560928
C	0.230002	1.389959	2.007486
C	-0.026140	0.594084	0.766308
H	0.662150	-0.259757	0.694145
H	-0.689380	1.610034	-0.746853
N	-1.409433	0.148295	0.759591
C	-1.816458	-0.715214	1.617114
C	-3.241343	-1.146953	1.508062
C	-3.839410	-1.280744	0.243349
H	-3.226187	-1.090810	-0.631016
C	-5.174927	-1.651504	0.113468
H	-5.612478	-1.758040	-0.874128
C	-5.949121	-1.887935	1.253037
H	-6.991420	-2.176898	1.157952
C	-5.386220	-1.749547	2.520928
H	-5.960711	-1.909561	3.426723
C	-4.050300	-1.382253	2.626942
F	-3.533044	-1.227737	3.865446
H	5.843813	0.246414	0.288537
H	4.757700	-2.221307	0.414239
H	4.376157	4.848736	-1.907138
H	2.223400	5.736496	-3.274801
H	-1.905574	-3.500355	-2.751129
H	0.263368	-4.397961	-1.416638
H	-3.479872	1.165075	-4.739798
H	-2.372791	3.620194	-4.899915

$^{4}RC_{C7}$

H	-0.927810	-0.892542	-4.988219
S	0.158393	-0.104331	-4.847937
N	2.125908	0.373685	-2.585321
C	3.125535	-0.557892	-2.686394
C	2.749783	1.597704	-2.605260
C	4.411442	0.091400	-2.777229
C	4.177573	1.430250	-2.732739
C	2.109707	2.822901	-2.503172
H	2.730443	3.712733	-2.532932
C	0.738361	3.007729	-2.353343
N	-0.191262	2.006402	-2.301517
C	-1.403402	2.626441	-2.155040
C	-1.234547	4.059135	-2.112100
C	0.099978	4.296659	-2.237047
C	-2.630362	1.979817	-2.072128
H	-3.512839	2.600493	-1.954624
C	-2.821281	0.605906	-2.124968
C	-4.108333	-0.040832	-2.040696
C	-2.452093	-1.544573	-2.273598
N	-1.826627	-0.327462	-2.271199
C	-3.878391	-1.379798	-2.132745
C	-1.812877	-2.774327	-2.408210
H	-2.436227	-3.662737	-2.395247
C	-0.448666	-2.958038	-2.556610
C	0.196781	-4.241865	-2.681846
N	0.489820	-1.948307	-2.580360
C	1.710901	-2.575628	-2.688126
C	2.938150	-1.934291	-2.716685
H	3.823970	-2.556527	-2.792327
Fe	0.161818	0.020362	-2.286163
C	1.533311	-4.005222	-2.759019
O	0.293045	-0.049172	-0.665107
C	0.059934	-0.208173	2.859418
C	0.363798	-1.550045	2.583432
H	-0.403711	-2.195055	2.173020
C	1.634418	-2.055652	2.822541
Cl	1.980827	-3.740465	2.460009
C	2.641607	-1.244354	3.342300
H	3.634645	-1.643120	3.515723
C	2.356670	0.089414	3.611165
H	3.141253	0.738783	3.980020
C	1.078481	0.617401	3.386499

N	0.828481	1.981490	3.678616
C	1.239255	2.745110	4.764835
C	1.977322	2.215146	5.953163
H	1.811458	2.906367	6.781259
H	1.626372	1.220209	6.243646
H	3.059803	2.151291	5.788108
N	0.840656	3.992600	4.638164
C	0.152284	4.059099	3.446204
H	-0.295887	4.981082	3.101480
C	0.123261	2.838033	2.830260
C	-0.537821	2.293266	1.611239
H	0.175795	1.767006	0.960444
H	-0.963698	3.118615	1.036060
N	-1.629125	1.382340	1.987617
C	-1.328580	0.274239	2.557751
C	-2.470494	-0.638957	2.876950
C	-3.547657	-0.753357	1.983240
H	-3.501079	-0.184899	1.061073
C	-4.639175	-1.570723	2.266371
H	-5.453651	-1.649324	1.552836
C	-4.682631	-2.289007	3.464513
H	-5.531052	-2.927396	3.692539
C	-3.633561	-2.185231	4.377024
H	-3.638542	-2.715911	5.322947
C	-2.552999	-1.366673	4.069623
F	-1.558746	-1.264609	4.980188
H	4.890758	2.242410	-2.779132
H	5.356765	-0.426307	-2.870309
H	0.618748	5.245839	-2.253009
H	-2.040938	4.772481	-2.006011
H	-0.321892	-5.191135	-2.698234
H	2.340009	-4.719722	-2.853598
H	-4.593738	-2.191200	-2.113860
H	-5.052009	0.476965	-1.931745

$^4TS_{C7}$

H	-1.268205	-0.572250	-4.296158
S	0.045604	-0.268437	-4.294773
N	2.267482	-0.168422	-2.358248
C	2.969222	-1.338675	-2.512523
C	3.176692	0.846421	-2.507665
C	4.357596	-1.050816	-2.776006
C	4.488416	0.303699	-2.763879
C	2.884740	2.202736	-2.448500
H	3.707712	2.897580	-2.580141
C	1.615128	2.737460	-2.285500
N	0.463131	2.012584	-2.125151
C	-0.561542	2.925557	-2.095962
C	-0.037163	4.265364	-2.221786
C	1.312999	4.149230	-2.331454
C	-1.907472	2.619503	-1.970610
H	-2.611154	3.445557	-1.977205
C	-2.426850	1.339114	-1.816362
C	-3.827047	1.044441	-1.638979
C	-2.588302	-0.834212	-1.556381
N	-1.691107	0.183756	-1.775363
C	-3.926563	-0.303413	-1.473299
C	-2.272544	-2.182057	-1.448556
H	-3.089102	-2.870981	-1.260318
C	-1.003863	-2.723138	-1.611254
C	-0.713501	-4.137475	-1.635991
N	0.136359	-2.002688	-1.857801
C	1.129745	-2.921192	-2.074121
C	2.447626	-2.618440	-2.388404
H	3.129298	-3.449764	-2.535313
Fe	0.311501	0.027840	-1.931740
C	0.608846	-4.259790	-1.932590
O	0.565880	0.177465	-0.232061
C	-0.062069	0.240191	2.187394
C	0.660641	-0.696006	1.345077
H	0.133761	-1.590830	1.036149
C	2.072476	-0.818296	1.575682
Cl	2.913163	-2.219008	0.972944
C	2.772917	0.165078	2.248881
H	3.852832	0.110930	2.329470
C	2.072319	1.214329	2.844702
H	2.616048	2.015359	3.328852
C	0.660695	1.227816	2.862089
N	-0.002342	2.246289	3.592831
C	0.317761	2.815759	4.820200
C	1.424553	2.359966	5.717817
H	1.182493	2.684898	6.731201
H	1.538285	1.271724	5.710032
H	2.394457	2.798869	5.454157
N	-0.523882	3.780659	5.123003
C	-1.421488	3.854559	4.080369
H	-2.236311	4.565638	4.083420
C	-1.131168	2.923295	3.122346
C	-1.793390	2.453674	1.873213
H	-1.084013	2.432706	1.033039
H	-2.605913	3.136654	1.614579

N	-2.362898	1.119363	2.102357
C	-1.548842	0.134640	2.244852
C	-2.170826	-1.207413	2.484869
C	-3.478564	-1.454410	2.029079
H	-3.970145	-0.659429	1.480611
C	-4.120992	-2.663462	2.275140
H	-5.130387	-2.823715	1.908388
C	-3.469061	-3.668363	2.996995
H	-3.963305	-4.615086	3.193530
C	-2.178957	-3.450987	3.474388
H	-1.644611	-4.197340	4.052070
C	-1.558957	-2.232660	3.217204
F	-0.316088	-2.053310	3.724209
H	5.379598	0.897201	-2.919414
H	5.120425	-1.800927	-2.937003
H	2.050493	4.932087	-2.449147
H	-0.640197	5.163660	-2.226276
H	-1.442728	-4.918332	-1.465685
H	1.192963	-5.162726	-2.051117
H	-4.815237	-0.899385	-1.313458
H	-4.616136	1.784538	-1.636323

$^{4}\mathrm{RC}_{\mathrm{C9}}$

H	0.924841	-0.301352	-5.811310
S	0.856004	0.895776	-5.192763
N	2.881263	-0.460303	-3.531608
C	3.262926	-1.696340	-4.006936
C	4.025012	0.306235	-3.522762
C	4.669125	-1.695336	-4.327327
C	5.140642	-0.455746	-4.027293
C	4.105530	1.614780	-3.075718
H	5.078053	2.094979	-3.112359
C	3.040669	2.367165	-2.595650
N	1.749540	1.922131	-2.475771
C	1.038014	2.974234	-1.951068
C	1.901831	4.110539	-1.743179
C	3.145396	3.734056	-2.145326
C	-0.314639	2.955278	-1.647727
H	-0.735851	3.849671	-1.203991
C	-1.166760	1.871101	-1.837433
C	-2.578067	1.885719	-1.537669
C	-1.939003	-0.099854	-2.378137
N	-0.797520	0.656159	-2.344855
C	-3.060048	0.659478	-1.880024
C	-2.016913	-1.414796	-2.820575
H	-2.991679	-1.891511	-2.792815
C	-0.950017	-2.176157	-3.278402
C	-1.052843	-3.550962	-3.701957
N	0.345676	-1.738362	-3.387871
C	1.061949	-2.803672	-3.863142
C	2.424565	-2.789590	-4.147804
H	2.863371	-3.708147	-4.524319
Fe	1.071551	0.051117	-2.798173
C	0.200028	-3.943052	-4.061924
O	1.378190	-0.453191	-1.276518
C	-1.702049	-0.621902	3.502614
C	-1.666640	-2.016865	3.659074
H	-2.172209	-2.472076	4.502810
C	-0.991799	-2.816145	2.745422
Cl	-0.981339	-4.559971	2.966664
C	-0.330374	-2.254219	1.654621
H	0.189165	-2.878761	0.936861
C	-0.360179	-0.874386	1.481672
H	0.126820	-0.450392	0.610341
C	-1.030709	-0.051823	2.396089
N	-1.039451	1.353663	2.194049
C	-0.009245	2.195285	1.798993
C	1.410980	1.768269	1.609380
H	2.031109	2.666051	1.622363
H	1.739425	1.098762	2.411072
H	1.575135	1.251665	0.657367
N	-0.443572	3.434171	1.668802
C	-1.784719	3.411075	1.986516
H	-2.385494	4.310734	1.981340
C	-2.183309	2.143994	2.314061
C	-3.444374	1.537463	2.824316
H	-3.735647	0.673967	2.204579
H	-4.250068	2.273374	2.766319
N	-3.287956	1.135383	4.227940
C	-2.479642	0.177399	4.505583
C	-2.384208	-0.208994	5.947755
C	-3.523978	-0.140055	6.766670
H	-4.453948	0.177138	6.307683
C	-3.466087	-0.461928	8.119628
H	-4.364236	-0.409655	8.726993
C	-2.252257	-0.852994	8.692088
H	-2.198690	-1.106056	9.746682
C	-1.100660	-0.915194	7.909238
H	-0.138484	-1.198314	8.321970
C	-1.184272	-0.592094	6.559709
F	-0.047742	-0.633502	5.830838

H	6.148013	-0.074724	-4.129169
H	5.208757	-2.543869	-4.726396
H	4.059613	4.312457	-2.143648
H	1.578919	5.058091	-1.334156
H	-1.969739	-4.125032	-3.714804
H	0.525853	-4.905401	-4.433629
H	-4.073253	0.287001	-1.808113
H	-3.106939	2.729817	-1.117439

$^{4}TS_{C9}$

H	1.353256	-1.063447	-5.043950
S	1.083196	0.209991	-4.689341
N	2.262730	-1.058626	-2.274272
C	2.583447	-2.385375	-2.383551
C	3.444254	-0.398549	-2.020489
C	4.000820	-2.573992	-2.188746
C	4.534893	-1.341499	-1.967945
C	3.571494	0.967671	-1.814162
H	4.566684	1.350073	-1.611262
C	2.528717	1.886803	-1.839951
N	1.209855	1.589770	-2.078833
C	0.525866	2.771512	-1.974772
C	1.433299	3.846582	-1.655401
C	2.678517	3.297780	-1.576993
C	-0.843162	2.925275	-2.166750
H	-1.252183	3.923667	-2.050913
C	-1.722207	1.914072	-2.523651
C	-3.128193	2.116601	-2.787576
C	-2.543106	-0.032694	-3.106319
N	-1.390673	0.596328	-2.718071
C	-3.635977	0.910467	-3.155701
C	-2.658988	-1.385002	-3.396319
H	-3.636174	-1.752207	-3.692415
C	-1.628492	-2.309838	-3.305582
C	-1.779740	-3.725091	-3.545892
N	-0.334914	-2.025078	-2.949597
C	0.333607	-3.226400	-2.950278
C	1.681771	-3.402851	-2.676590
H	2.070722	-4.414773	-2.724456
Fe	0.399109	-0.244534	-2.429514
C	-0.565677	-4.293884	-3.318837
O	0.029163	-0.503516	-0.771816
C	-0.800267	0.144143	2.852035
C	-0.458482	-1.184620	2.515473
H	-0.926565	-2.000750	3.053367
C	0.465446	-1.459843	1.534532
Cl	0.894077	-3.115376	1.180689
C	1.044774	-0.417478	0.735482
H	1.976661	-0.629394	0.225242
C	0.807032	0.920931	1.190687
H	1.305784	1.730370	0.676710
C	-0.120929	1.201467	2.175737
N	-0.373722	2.552755	2.534729
C	0.512155	3.608036	2.704541
C	2.001747	3.490947	2.646417
H	2.423479	4.332097	3.199465
H	2.356281	2.556484	3.091886
H	2.384838	3.538812	1.620108
N	-0.133821	4.724185	2.971192
C	-1.473161	4.403769	2.977351
H	-2.233162	5.144832	3.184855
C	-1.655283	3.073760	2.711575
C	-2.829340	2.157301	2.686557
H	-2.861772	1.591657	1.741389
H	-3.748077	2.745031	2.752521
N	-2.787325	1.240675	3.832845
C	-1.849957	0.360952	3.888037
C	-1.891307	-0.563266	5.065453
C	-3.128083	-0.987151	5.579544
H	-4.025548	-0.642726	5.077548
C	-3.204507	-1.814543	6.696477
H	-4.173237	-2.134775	7.067043
C	-2.034277	-2.231295	7.337271
H	-2.085412	-2.876583	8.209094
C	-0.792468	-1.814548	6.860331
H	0.135664	-2.104343	7.340863
C	-0.742372	-0.991336	5.741367
F	0.473143	-0.581382	5.314543
H	5.567847	-1.077580	-1.783858
H	4.505287	-3.530428	-2.224643
H	3.619241	3.789289	-1.366723
H	1.141899	4.879350	-1.518235
H	-2.704158	-4.202660	-3.842313
H	-0.284155	-5.335941	-3.392691
H	-4.650855	0.661894	-3.436271
H	-3.637689	3.067580	-2.705943

$^{4}RC_{C10}$

H	0.837354	-1.039046	-5.397573
S	1.023567	0.199291	-4.895219

N	1.876768	-1.442554	-2.604743
C	1.923528	-2.793695	-2.868684
C	3.164963	-1.081435	-2.274802
C	3.272589	-3.284238	-2.726540
C	4.040818	-2.224161	-2.359678
C	3.564807	0.188100	-1.894064
H	4.613720	0.328630	-1.653031
C	2.735964	1.301488	-1.811286
N	1.391870	1.308035	-2.076914
C	0.971940	2.598946	-1.865445
C	2.082259	3.427216	-1.462005
C	3.179399	2.620778	-1.429044
C	-0.328447	3.055009	-2.020270
H	-0.520273	4.098195	-1.793869
C	-1.409939	2.279004	-2.424811
C	-2.748479	2.786508	-2.609073
C	-2.631664	0.590264	-3.077191
N	-1.359427	0.944315	-2.715419
C	-3.509044	1.735204	-3.018361
C	-3.034198	-0.686730	-3.448078
H	-4.078985	-0.822225	-3.709382
C	-2.215768	-1.807166	-3.498667
C	-2.666033	-3.129636	-3.859317
N	-0.874634	-1.820674	-3.213059
C	-0.469684	-3.118025	-3.376853
C	0.833577	-3.577044	-3.211698
H	1.013571	-4.634232	-3.378976
Fe	0.226438	-0.282918	-2.509737
C	-1.580473	-3.946432	-3.779458
O	-0.109513	-0.625938	-0.953296
C	-0.091938	-0.011486	3.158034
C	0.756696	-1.060782	3.544517
H	0.362764	-1.869302	4.149142
C	2.090761	-1.075244	3.160068
Cl	3.121942	-2.408463	3.657319
C	2.620558	-0.049401	2.379333
H	3.658084	-0.077029	2.066869
C	1.790645	0.993730	1.985663
H	2.179742	1.771065	1.339789
C	0.445824	1.033551	2.373495
N	-0.363972	2.122465	1.963036
C	-0.067451	3.478433	1.921755
C	1.201291	4.091362	2.423482
H	1.003458	5.145041	2.628167
H	1.552623	3.611007	3.341784
H	2.013591	4.039620	1.688666
N	-1.079155	4.164758	1.431916
C	-2.066488	3.245348	1.147084
H	-3.022277	3.545201	0.740004
C	-1.657017	1.978240	1.460146
C	-2.292536	0.630839	1.443199
H	-1.660746	-0.080319	0.889468
H	-3.253497	0.693796	0.927256
N	-2.537616	0.170547	2.817783
C	-1.532250	-0.084581	3.572397
C	-1.847870	-0.574354	4.951191
C	-2.966922	-1.397624	5.160900
H	-3.558359	-1.670561	4.293816
C	-3.310748	-1.845033	6.433458
H	-4.175079	-2.488428	6.565254
C	-2.541749	-1.466420	7.537632
H	-2.803112	-1.811375	8.533537
C	-1.434654	-0.637432	7.363791
H	-0.824071	-0.309777	8.198176
C	-1.109996	-0.206356	6.082664
F	-0.047586	0.618555	5.946198
H	5.103320	-2.199867	-2.157481
H	3.573047	-4.311011	-2.887474
H	4.201222	2.880742	-1.185728
H	2.012662	4.485371	-1.248349
H	-3.681825	-3.381416	-4.133587
H	-1.518217	-5.008531	-3.975730
H	-4.562923	1.717674	-3.262293
H	-3.044567	3.813840	-2.445337

$^{4}TS_{C10}$

H	1.158402	-1.148740	-4.782033
S	1.063250	0.175612	-4.543263
N	2.160448	-1.061784	-2.066436
C	2.267675	-2.427161	-2.041334
C	3.434238	-0.572588	-1.905075
C	3.644382	-2.814805	-1.849434
C	4.368979	-1.664110	-1.771028
C	3.779607	0.771268	-1.848340
H	4.830704	1.010800	-1.723371
C	2.889266	1.833940	-1.932068
N	1.525553	1.723188	-2.070023
C	1.035174	3.000509	-2.067450
C	2.112897	3.948053	-1.921822
C	3.264669	3.223769	-1.843943
C	-0.305389	3.347088	-2.207646

H	-0.554396	4.401456	-2.157094
C	-1.341857	2.456651	-2.435476
C	-2.713172	2.849232	-2.664776
C	-2.468265	0.618230	-2.833583
N	-1.219543	1.091977	-2.533915
C	-3.410069	1.710594	-2.920658
C	-2.796831	-0.718857	-3.011465
H	-3.828854	-0.954512	-3.249787
C	-1.913757	-1.779598	-2.871683
C	-2.285827	-3.169472	-2.982413
N	-0.579178	-1.669591	-2.571197
C	-0.101266	-2.955528	-2.478553
C	1.212195	-3.314995	-2.215647
H	1.439339	-4.375085	-2.171560
Fe	0.436727	0.012836	-2.235081
C	-1.165113	-3.897919	-2.730557
O	0.114532	-0.062889	-0.542638
C	-0.588570	-0.317007	2.667291
C	-0.000766	-1.594157	2.773873
H	-0.440282	-2.331358	3.434709
C	1.165653	-1.905171	2.065448
Cl	1.905509	-3.482233	2.314411
C	1.754268	-1.001193	1.209086
H	2.667512	-1.243498	0.682062
C	1.089633	0.242469	0.944488
H	1.685505	1.033659	0.507898
C	0.005148	0.629471	1.823464
N	-0.520412	1.930763	1.704113
C	0.139436	3.155890	1.671518
C	1.611311	3.328777	1.868804
H	1.783929	4.347079	2.221909
H	2.006755	2.627237	2.610120
H	2.178095	3.201248	0.939399
N	-0.715283	4.143513	1.521643
C	-1.967022	3.566735	1.452010
H	-2.860700	4.164416	1.335578
C	-1.882650	2.207151	1.565556
C	-2.860181	1.087648	1.660937
H	-2.606554	0.300090	0.935151
H	-3.861103	1.455666	1.424409
N	-2.876663	0.552355	3.030375
C	-1.830693	-0.049519	3.466855
C	-1.910471	-0.591495	4.858996
C	-3.128241	-1.097776	5.343812
H	-3.976734	-1.097182	4.668398
C	-3.247670	-1.579188	6.644399

H	-4.198077	-1.973722	6.989897
C	-2.143710	-1.554672	7.501544
H	-2.228644	-1.928554	8.517448
C	-0.926151	-1.043728	7.054843
H	-0.052815	-0.993541	7.695992
C	-0.831243	-0.571663	5.750853
F	0.353293	-0.056230	5.352373
H	5.436105	-1.547677	-1.635370
H	3.994016	-3.836966	-1.791581
H	4.279978	3.581616	-1.735260
H	1.986963	5.021924	-1.888375
H	-3.281852	-3.522158	-3.215055
H	-1.048302	-4.973476	-2.716690
H	-4.462024	1.598803	-3.147661
H	-3.071097	3.869783	-2.639262
